# Nickel-Catalyzed Intermolecular Cyclization of 2-Bromobenzamide: A General Strategy for Synthesizing 6(5H)-Phenanthridinone Derivatives

**DOI:** 10.3390/molecules31071176

**Published:** 2026-04-02

**Authors:** Xinsheng Xiao, Xueli Zhu, Yan Shu, Bowen Zhang, Changhui Zhao, Asad Nawaz, Zunhua Li

**Affiliations:** 1 College of Chemistry and Bioengineering, Hunan University of Science and Engineering, Yongzhou 425199, China; shengguoxiao@163.com (X.X.); 18256291388@163.com (X.Z.); 15897318158@139.com (Y.S.); zchui112@163.com (C.Z.); 007298@yzu.edu.cn (A.N.); 2 School of Chemical Engineering and Technology, Tianjin University, Tianjin 300072, China; bowen_667@tju.edu.cn

**Keywords:** nickel catalysis, phenanthridinone, intermolecular cyclization, 2-bromobenzanilide

## Abstract

6(5H)-phenanthridinone derivatives, as an important class of alkaloids, have broad application value in drug development and functional material synthesis. In this study, a nickel-catalyzed synthetic strategy was developed, using 2-bromobenzamide compounds as starting materials. Through an intermolecular cyclization reaction, a series of 6(5H)-phenanthridinone derivatives bearing amide substituents was efficiently constructed. The optimal reaction system was identified: Ni(acac)_2_/Zn as the catalyst, PCy_3_ as the ligand, toluene as the solvent, Cs_2_CO_3_ as the base, under an argon atmosphere at 150 °C for 12 h. The target products were obtained in yields up to 88%. Further substrate scope exploration demonstrated the excellent generality of this method, successfully synthesizing 21 derivatives with various substitution patterns, achieving yields ranging from 51% to 92%, and showing good compatibility with multiple functional groups such as alkyl, aryl, and heterocyclic moieties. Importantly, the reaction remained stable during gram-scale experiments, successfully yielding the desired compound at 85%. This work not only provides an approach for the precise construction of the 6(5H)-phenanthridinone framework but also opens an efficient pathway for the controlled synthesis of amide-substituted derivatives.

## 1. Introduction

6(5H)-Phenanthridinones are fused heterocyclic structures widely present in natural alkaloids and pharmaceutical molecules [1,2,3], exhibiting significant biological activities including antitumor, antiviral, and acetylcholinesterase inhibitory effects [4,5]. Some derivatives have been applied in treating myasthenia gravis, myopathies, and neurological disorders [6,7]. This scaffold is commonly found in Amaryllidaceae alkaloids from plants such as Daffodils and Clivia. For instance, hippadine from Lycoris radiata reversibly inhibits male rat fertility [5]. Kalbretorine from Tacca exhibits anticancer activity [8], and hydrogenated derivatives like lycorine show potent antitumor effects via acetylcholinesterase inhibition, disruption of DNA/protein synthesis, and impairment of mitochondrial function [9]. The broad application prospects of 6(5H)-phenanthridinones have driven interest in developing efficient, versatile, and economical synthetic methods [10,11,12].

**Scheme 1:**
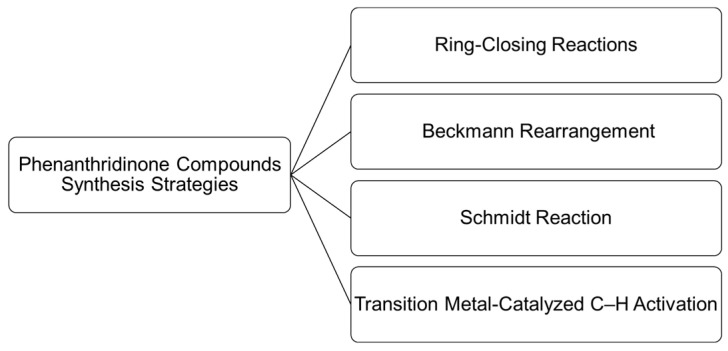
Phenanthridinone compounds synthesis strategies.

Current phenanthridinone synthesis relies on four classic strategies: ring-closing reactions, Beckmann rearrangement, Schmidt reaction, and transition metal-catalyzed C–H activation (Scheme 1). The ring-closing approach proceeds in two steps: Suzuki cross-coupling of dialkyl (2-fluorophenyl)boronate with 2-halobenzonitrile yields a 2-fluorobiphenyl-2-nitrile intermediate, followed by KOH-induced anionic cyclization [13]. Beckmann rearrangement converts ketoximes to amides under acidic conditions; for example, 9-fluorenone oxime rearranges to phenanthridinone at 175–180 °C with trans configuration retention [14]. The intramolecular Schmidt reaction employs azides: 9-fluorenones undergo ring formation in NaN_3_/H_2_SO_4_ to afford phenanthridinone [15]. Palladium-catalyzed C–H activation offers high atom economy, typically using Pd(OAc)_2_/norbornene cooperative catalysis in CH_3_CN/DMF with K_2_CO_3_ for one-step cyclization [16].

Moreover, transition metals are crucial in phenanthridinone synthesis. Intramolecular cyclization typically requires halogens (Br, Cl, I) on the phenyl rings for activation, giving satisfactory yields. Alternatively, direct C–H bond oxidative activation can provide moderate yields without halogens. In 1996, Harayama et al. first reported Pd(OAc)_2_/PPh_3_-catalyzed cyclization of N-methyl-N-phenyl-o-(X-substituted)benzamide (X = Br/I), achieving up to 98% yield [17]. The following year, they improved yield stability by replacing PPh_3_ with dppp/P(OPh)_3_ and using diisopropylethylamine as base [18]. In 2005, they confirmed that when X is on the amide phenyl ring, palladium catalysis remains highly efficient; oxygen at the carbonyl phenyl meta-position induced configurational changes, affording up to 99% yield [19]. Campeau et al. employed an imidazolium salt/Pd(IV) system with K_2_CO_3_ in DMA at 130 °C for 18 h, achieving 97% yield—more environmentally friendly but costly [20]. In 2010, Yeung et al. developed a green approach using Pd(OAc)_2_/Na_2_S_2_O_8_ in TFA/DCE at 70 °C via double C–H activation, obtaining moderate yields [21]. Boonya-Udtayan et al. utilized Cu(I) thiophene-2-carboxylate under microwave conditions for rapid synthesis, achieving up to 96% yield [22].

Transition metal-catalyzed intermolecular reactions provide diverse strategies for quinolinone synthesis. For instance, two identical 2-bromobenzamide molecules undergo palladium-catalyzed coupling under Pd(OAc)_2_/L with Cs_2_CO_3_ in 1,4-dioxane for 24 h, tolerating both electron-donating and -withdrawing substituents [23]. For non-halogenated substrates such as iodobenzene and benzamide derivatives, coupling proceeds at 120 °C via C–H/C–I/N–H activation using Pd(OAc)_2_/Ag_2_O/AcOH [24]. Non-iodinated benzene substrates react at room temperature with an N-methoxybenzamide/benzene/TFA/Pd(OAc)_2_/K_2_S_2_O_8_ system [25]. Other approaches include one-pot Suzuki coupling/amidation between methyl 2-chlorobenzoate and 2-aminophenylboronic acid catalyzed by Pd/phosphine ligands [26], as well as borylation of 2-aminobiphenyl followed by Pd/CO carbonylation. These methods enhance the flexibility and practicality of quinolinone synthesis.

Despite various reported strategies for phenanthridinone synthesis, most suffer from limitations, including expensive materials, precious metal catalysts, or narrow substrate scope. This study develops an efficient nickel-catalyzed approach using readily available 2-bromobenzamides via intramolecular cyclization to construct amide-substituted 6(5H)-phenanthridinones. Key parameters were systematically optimized for high yields. Substrate scope was extensively explored, followed by gram-scale validation. This work provides a general and economical strategy for the precise construction of the phenanthridinone framework.

## 2. Results and Discussion

### 2.1. Optimization Results of Reaction Conditions

**Scheme 2:**
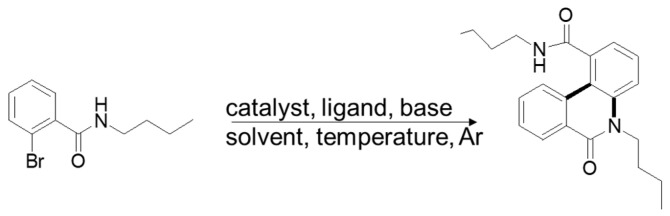
Synthesis of 6(5H)-phenanthridinone derivatives (N,5-dibutyl-6-oxo-5,6-dihydrophenanthridine-4-carboxamide) by using 2-bromobenzoyl butylamine.

As shown in Scheme 2, using 2-bromobenzoyl butylamine as the model substrate, an intermolecular cyclization reaction was carried out under a nickel catalyst in an argon atmosphere. The effects of various reaction conditions, including the catalyst, ligand, solvent, base, and temperature, on the reaction were investigated.

#### 2.1.1. Results of Catalyst Screening

To optimize the catalytic system, a systematic screening was conducted under standardized conditions. Triphenylphosphine (PPh_3_, 10 mol%) served as the ligand to stabilize the nickel center via its electron-donating and steric properties, which can prevent the metal from decomposing or becoming inactive. Toluene was chosen as the solvent for its thermal stability and low polarity, while Na_2_CO_3_ (2.0 eq.) provided mild basicity to facilitate deprotonation without decomposition. Reactions were performed at 0.1 M substrate concentration under argon at 150 °C for 12 h. This study evaluated commercially available metal catalysts at 5 mol% loading with Zn powder (1.5 eq.) as reducing agent. Products were purified by column chromatography and characterized by ^1^H, ^13^C, ^19^F NMR and HRMS. Isolated yields were determined using the internal standard method.

Table 1 summarizes the catalyst screening results. CuI failed to obtain the products (entry 1), likely due to disproportionation or formation of inactive complexes under the conditions. Palladium catalysts (PdCl_2_, Pd(OAc)_2_) afforded the product in low yields of 24% and 35%, respectively (entries 2–3), underscoring the trade-off between cost and efficiency. In contrast, nickel-based systems with Zn additive proved more effective. NiCl_2_·6H_2_O, Ni(COD)_2_, NiBr_2_, and Ni(acac)_2_ all facilitated the cyclization, delivering isolated yields ranging from 31% to 64% (entries 4–7). Among these, Ni(acac)_2_/Zn performed best (64% yield), representing a 22–33% improvement over other nickel catalysts (entry 7). This superior result is attributed to the high solubility and thermal stability of Ni(acac)_2_, along with its synergistic effect with the PPh_3_ ligand, which promotes efficient nickel reduction and substrate insertion [27,28]. Moreover, nickel offers significant cost advantages over palladium, and the use of Zn effectively suppresses catalyst oxidation and deposition, enhancing system reliability.

Furthermore, no target product was detected in the absence of a catalyst (entry 8), underscoring the essential role of nickel in lowering the activation energy and stabilizing key intermediates. Nickel species facilitate bond cleavage (e.g., C–Br) through coordination or oxidative addition, enabling otherwise inaccessible reaction pathways. Without catalytic mediation, the kinetic barrier remains insurmountable under identical conditions, resulting in negligible conversion. This control experiment confirms the thermodynamic and kinetic necessity of nickel participation for successful cyclization.

This screening established Ni(acac)_2_/Zn as an efficient catalytic system, revealing that the ligand environment of nickel critically governs reaction kinetics, while Zn serves an indispensable reducing role [29,30]. This finding underpins the development of low-loading, high-turnover nickel catalysis and supports its economic viability for industrial scale-up [31]. Table 1 provides quantitative performance data, offering mechanistic insights and guiding process design.

#### 2.1.2. Results of Ligand Screening

We further investigated the effect of the ligand on the reaction operating in toluene with 0.1 M substrate, 5 mol% of Ni(acac)_2_ with 1.5 equivalents of zinc powder, 2 equivalents of Na_2_CO_3_ at 150 °C for 12 h under inter conditions. As shown in Table 2, all selected ligands facilitated the target transformation with varying yields. The monodentate phosphine PPh_3_ afforded a 64% yield (entry 1), while the bidentate ligand dppp slightly improved it to 68% (entry 2). Chiral BINOL and bulky BINAP delivered 62% and 65% yields, respectively (entries 3–4). Notably, the electron-rich and sterically bulky ligand PCy_3_ exhibited outstanding performance, achieving a record 78% yield—approximately 14 percentage points higher than PPh_3_ (entry 5). This is attributed to PCy_3_’s strong electron-donating ability, which enhances Ni(0) nucleophilicity, and its steric bulk, which effectively suppresses side reactions [32,33]. Moreover, a control experiment confirmed the indispensable role of the ligand, as its omission led to a sharply diminished yield of only 27% (entry 6).

In-depth analysis reveals that ligand properties critically govern catalytic kinetics and stability: strong σ-donors like PCy_3_ accelerate low-valent nickel formation, shortening the induction period [34]. Moderate steric hindrance balances substrate binding and product release, and heteroatom coordination modes (O^N vs. P^P) modulate the metal’s microenvironment polarity [28]. Although ligands such as BINAP and PCy_3_ are relatively costly, their yield enhancements provide essential parameters for process economic evaluation. These experiments establish PCy_3_ as the optimal ligand and offer empirical insights into ligand-metal cooperative mechanisms, guiding the development of efficient ligand libraries in nickel catalysis.

#### 2.1.3. Results of Solvent Screening

To enhance economic and environmental efficiency, this study systematically investigated solvent effects on reaction performance using the optimal Ni(acac)_2_/Zn-PCy_3_ system. Representative aprotic solvents—toluene, 1,2-dichloroethane (DCE), acetonitrile (CH_3_CN), DMSO, and DMF—were selected. By comparing dielectric constants (ε), hydrogen-donating abilities (α), and Lewis basicity, solvent–substrate interaction mechanisms were elucidated.

The experimental data are summarized in Table 3. Solvent type plays a decisive role in reaction outcome. Strongly polar aprotic solvents DMSO and DMF completely inhibited the reaction (entries 4–5), likely due to over-stabilization of Ni(II) precursors hindering low-valent nickel formation, and their electron-withdrawing nature suppressing oxidative addition. Moderately polar solvents showed varied performance: 1,2-DCE afforded a 47% yield (entry 2), while acetonitrile delivered only 25% (entry 3), possibly due to cyano coordination interfering with the catalytic cycle. Remarkably, the nonpolar aromatic solvent toluene achieved the highest yield of 78% (entry 1)—31% higher than 1,2-DCE. This outstanding performance is attributed to toluene’s dual advantages: its low polarity promotes hydrophobic substrate enrichment, increasing collision frequency; and its high boiling point (110.6 °C) ensures thermal stability and minimizes volatilization losses.

Further analysis reveals that solvent effects regulate reaction kinetics through multiple dimensions: dielectric shielding reduces electrostatic repulsion between charged intermediates, accelerating cyclization [35,36]; secondary interactions between aromatic π systems and nickel d orbitals stabilize transition states [37,38]; and solvent viscosity influences mass transfer, with toluene’s moderate fluidity balancing diffusion control and surface reactions. Although 1,2-DCE offers industrial cost advantages, its toxicity limits practical use. These experiments establish toluene as the optimal solvent and guide the development of green alternatives [39]. Future research will explore supercritical fluids or ionic liquid media for sustainable chemical transformations.

#### 2.1.4. Results of Bases Screening

To elucidate the critical role of base additives in the nickel-catalyzed system, this study systematically investigated six typical inorganic/organic bases—potassium acetate (KOAc), potassium tert-butoxide (KOtBu), sodium hydroxide (NaOH), triethylamine (NEt_3_), potassium carbonate (K_2_CO_3_), and cesium carbonate (Cs_2_CO_3_)—based on the previously optimized Ni(acac)_2_/Zn-PCy_3_-toluene system.

The experimental data are summarized in Table 4. Base nature significantly influences reaction progress. Weak organic base NEt_3_ completely inhibited activity (entry 4), likely due to poor nucleophilicity and steric hindrance impeding deprotonation. Strong base KOtBu afforded a moderate 80% yield (entry 2), suggesting excessive activation may promote side reactions. Oxygen-containing salts showed differentiated performance: KOAc achieved 82% yield (entry 1) via carboxylate chelation; carbonates exhibited a progressive trend, with K_2_CO_3_ and Cs_2_CO_3_ delivering 85% and 88% yields, respectively (entries 5–6). Cs_2_CO_3_’s superior performance (3% improvement) is attributed to cesium’s large ionic radius weakening lattice energy, its low charge density releasing more free OH^−^, and its soft acid character matching the nickel center’s d orbitals to form stable ion-pair intermediates. Notably, NaOH, despite the strongest theoretical basicity, gave only 69% yield (entry 3) due to hygroscopicity and corrosiveness, demonstrating that simply pursuing high pH is not optimal.

In-depth mechanistic analysis reveals that the base plays a dual role: acting as a proton shuttle to facilitate α-C–H bond cleavage and stabilizing the negatively charged transition state via electrostatic interactions [40]. Cs_2_CO_3_ offers key advantages: (1) moderate basicity balances deprotonation rate with intermediate stability; (2) unique solvation behavior of Cs^+^ enhances solid–liquid mass transfer; and (3) maintains ionic mobility even at lower temperatures [41]. NEt_3_ fails due to structural limitations—its tertiary amine group cannot effectively abstract the weakly acidic α-C–H proton and tends to compete for nickel coordination [42]. These experiments establish Cs_2_CO_3_ as the optimal base and provide empirical insights into base-metal cooperative mechanisms, guiding development of novel composite base systems.

#### 2.1.5. Results of Temperature Screening

To investigate the influence of temperature, six different temperatures, varying from 90 to 190 °C, were applied to the synthesis. The experimental data are summarized in Table 5. The results demonstrate that temperature exerts a significant influence on the reaction progress. When the temperature was elevated from 90 to 150 °C (entries 1–4), the yields exhibited a substantial increase, rising from 47% to 78%. According to the Arrhenius equation, an increase in temperature significantly enhances the reaction rate constant, thereby accelerating the overall reaction rate. For most organic synthesis reactions, within the temperature range where the catalyst maintains its activity, elevating the temperature generally facilitates the activation of reactant molecules. This process involves reducing the activation energy of the reaction and increasing the effective collision frequency, both of which contribute to an enhancement in the conversion rate. Moreover, nickel-based catalysts require activation within a specific temperature window, typically ranging from 80 to 120 °C. If the temperature falls below this range, the formation rate of C-C bonds becomes sluggish, resulting in a low conversion rate. However, when the temperature was further increased from 150 to 190 °C (entries 4–6), a notable decline in yields was observed, decreasing from 78% to 61%. This phenomenon can be attributed to the fact that high temperatures may trigger a greater number of side reactions, such as cracking and isomerization. These competing side reactions consume reactants and reduce the selectivity towards the target product, ultimately leading to a decrease in overall yield. Furthermore, the catalyst deactivation is another reason.

### 2.2. Expansion of Reaction Substrates

**Scheme 3:**
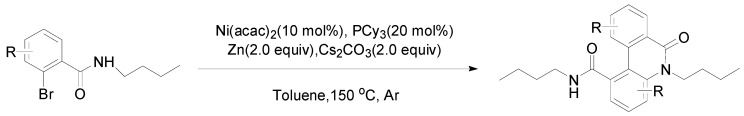
Expansion of substrates.

After establishing the optimal reaction conditions, we evaluated the substrate scope of this nickel-catalyzed system (Scheme 3). Under standard conditions (Ni(acac)_2_/Zn, PCy_3_, Cs_2_CO_3_, toluene, Ar, 150 °C, 12 h), various 2-bromobenzamide derivatives underwent cyclization to afford phenanthridinones in moderate to excellent yields (51–92%, Table 6). The reaction showed broad compatibility with nitrogen substituents. For linear alkyl groups (**2a**–**2d**), yields gradually decreased with increasing chain length, likely due to steric effects [43]. Branched alkyl substrates (**2e**, **2g**) gave significantly lower yields than their linear counterparts, highlighting steric hindrance as a critical factor. Cycloalkyl (**2f**) and hydroxyalkyl (**2h**) substrates reacted smoothly in moderate yields, demonstrating tolerance toward polar functional groups [44,45].

Notably, N-aryl-substituted substrates exhibited excellent reactivity [46]. Phenyl (**2i**) and various alkylphenyl derivatives (**2j**–**2m**) were efficiently converted in good yields. Within the alkylphenyl series, longer alkyl chains (**2l**, **2m**) generally afforded higher yields, likely due to enhanced electron-donating effects or improved solubility. However, branched alkyl chains (**2k**) led to a slight yield decrease, further confirming steric hindrance as a sensitive factor in this reaction. It should be noted that for product **2l**, due to the presence of a chiral carbon in the starting material, the resulting product contains two chiral centers and exists as diastereomers. Because the properties of diastereomers are very similar, they could not be separated by column chromatography, and the product was obtained as a mixture. The similar properties of the mixture led to closely overlapping NMR signals, resulting in peak broadening. In summary, this nickel-catalyzed system efficiently accommodates diverse 2-bromobenzamides for phenanthridinone synthesis. Steric and electronic effects significantly influence yields, providing mechanistic insights and guiding substrate design.

Having established the optimal conditions and evaluated N-substituent effects, we further investigated the influence of substituents on the benzene ring. As shown in Scheme 3, using 2-bromo-N-butylbenzamide as the model substrate, we systematically explored how various substituents (halogens, nitro, alkyl, alkoxy) at different positions (4-, 5-, and 6-positions) affect reaction activity to assess the method’s generality and functional group tolerance. The results, including positional effects on yields, are summarized in Table 7.

The experimental results demonstrate good adaptability of this catalytic system toward phenyl-ring-substituted substrates. Halogenated substrates (fluorinated **2n**, **2r**; chlorinated **2t**) afforded good to excellent yields, with chlorine generally outperforming fluorine—suggesting that electronic effects (inductive vs. conjugative) significantly influence efficiency. Substituent position also played a critical role: 4-fluoro substitution (**2n**) gave higher yields than 6-fluoro (**2r**), indicating pronounced steric hindrance at the 6-position, which is closer to the catalytic site.

Nitro-substituted (**2o**), alkyl-substituted (**2p**, **2q**, **2u**), and alkoxy-substituted (**2s**) substrates all reacted smoothly in good yields. Among methyl-substituted analogs, 4- and 5-methyl (**2p**, **2u**) performed similarly, while 6-methyl (**2q**) showed reduced yield—further confirming steric sensitivity at the 6-position. Notably, electron-donating methoxy (**2s**) afforded significantly higher yield than electron-withdrawing nitro (**2o**), suggesting an electron-rich phenyl ring favors cyclization, possibly via electrophilic activation or electron transfer pathways. Furthermore, the structure of a representative product (**2p**) was unequivocally confirmed by full 2D NMR (HSQC and HMBC) analysis; the detailed assignments are provided in the Supplementary Materials.

In summary, this nickel-catalyzed system tolerates diverse functional groups (halogens, nitro, alkyl, alkoxy) on the phenyl ring. Yields are governed jointly by electronic and positional effects: electron-donating groups promote the reaction, while substitution at the sterically hindered 6-position consistently reduces yield. These insights guide the design of complex phenanthridinone derivatives.

### 2.3. Gram-Scale Amplification Experimental Results

**Scheme 4:**
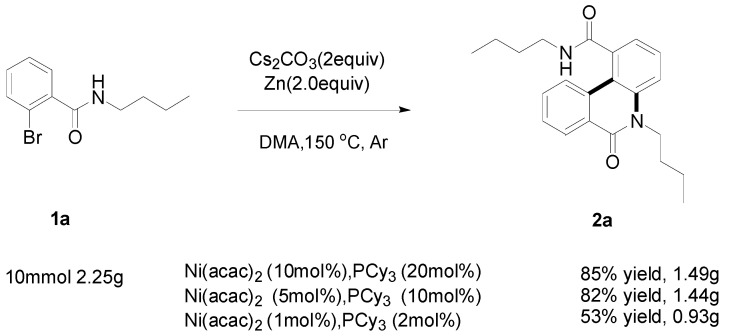
Gram-scale amplification experiment.

To evaluate the application potential of this reaction system, gram-scale amplification experiments were systematically conducted (Scheme 4). Using substrate 1a (10 mmol, 2.55 g) under standard conditions, the target product **2a** was obtained in 85% isolated yield (1.49 g), consistent with milligram-scale results and validating reaction stability during scale-up.

When catalyst and ligand loadings were halved to 50% of the original conditions, reaction efficiency remained stable with comparable yield, indicating high tolerance and untapped catalytic potential. However, further reduction to 1 mol% catalyst caused a significant yield drop to 53%, revealing a nonlinear relationship between catalyst concentration and efficiency and emphasizing the need for precise dosage control.

From an engineering perspective, this system offers three advantages: scalability validated by gram-scale yield, robustness to moderate catalyst loading adjustments, and optimization potential despite the 53% yield floor. These features support industrial applications, particularly in pharmaceutical intermediates and functional materials. Future work may focus on catalyst modification, mechanistic studies, and process optimization to advance green chemistry goals.

## 3. Materials and Methods

### 3.1. Materials

As shown in the Supplementary Table S1, the commercial reagents used in the experiment were purchased from suppliers such as Bide Medicine and Adamas, with purity grades all at analytical reagent (AR) level or higher.

### 3.2. Methods

#### 3.2.1. Preparation of Raw Materials

**Scheme 5:**
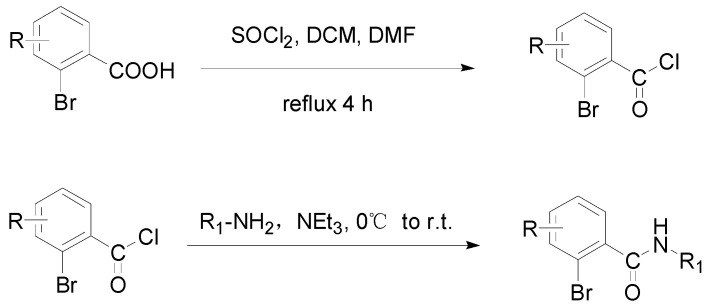
Preparation of 2-bromobenzamide derivatives.

As shown in Scheme 5, 2-bromobenzamide derivatives were synthesized from 2-bromobenzoic acid. 2-Bromobenzoic acid (5.0 mmol) was dissolved in DCM (20 mL), then SOCl_2_ (4 mL) and DMF (0.25 mmol) were added. The mixture was stirred at 40 °C for 4 h, and the solvent was removed to yield 2-bromobenzoyl chloride. The residue was redissolved in DCM (20 mL) and cooled to 0 °C. Triethylamine (1.5 mL) and the corresponding amine (10.0 mmol, 2.0 eq.) were added dropwise. The mixture was stirred overnight at room temperature, then quenched with saturated NaHCO_3_ (10 mL) and extracted with DCM (3 × 15 mL). The combined organic layers were dried over Na_2_SO_4_, concentrated, and purified by silica gel column chromatography (petroleum ether/ethyl acetate = 5:1, *v*/*v*, 1 h) to afford the pure 2-bromobenzamide derivatives.

#### 3.2.2. Optimization of Reaction Conditions

As shown in Scheme 2, 2-bromobenzoyl butylamine was used as the model substrate, under an argon atmosphere, an intermolecular cyclization reaction catalyzed by nickel was carried out. By changing one variable at a time, the effects of catalyst, ligand, solvent, base, and temperature on the reaction were systematically investigated. (1) Catalyst screening: Used PPh_3_ as the ligand, toluene as the solvent, Na_2_CO_3_ as the base, and at 150 °C, the effects of different catalysts were examined. (2) Ligand screening: Used Ni(acac)_2_ as the catalyst, toluene as the solvent, Na_2_CO_3_ as the base, and at 150 °C, the effects of different ligands were studied. (3) Solvent screening: Used Ni(acac)_2_ as the catalyst, PCy_3_ as the ligand, Na_2_CO_3_ as the base, and at 150 °C, the effects of different solvents were evaluated. (4) Base screening: Used Ni(acac)_2_ as the catalyst, PCy_3_ as the ligand, toluene as the solvent, and at 150 °C, the effects of different bases were investigated. (5) Temperature screening: Used Ni(acac)_2_ as the catalyst, PCy_3_ as the ligand, toluene as the solvent, and Na_2_CO_3_ as the base, the effects of different temperatures were investigated.

A dry reaction tube was charged with a magnetic stir bar, metal catalyst (10 mol%), zinc powder (2.0 eq.), ligand (20 mol%), base (2.0 eq.), solvent (1.0 mL), and 2-bromobenzoyl butylamine (0.20 mmol). The tube was evacuated and backfilled with argon, then placed in a 150 °C oil bath for 12 h. After cooling to room temperature, saturated brine was added, and the mixture was extracted with ethyl acetate (20 mL × 3). The combined organic layers were dried over anhydrous Na_2_SO_4_, concentrated under reduced pressure, and purified by silica gel column chromatography (petroleum ether/ethyl acetate = 5:1, *v*/*v*, 1.5 h) to afford the product, whose yield was calculated.

#### 3.2.3. Experiment on Expanding the Scope of Reaction Substrates

**Scheme 6:**
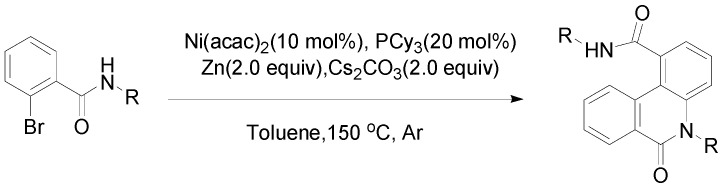
Expansion of Reaction Substrates.

As shown in Scheme 6, the optimal conditions were established as: Ni(acac)_2_ (10 mol%), PCy_3_ (20 mol%), Cs_2_CO_3_ (2.0 eq.), toluene (1.0 mL), under argon at 150 °C for 12 h. The substrate scope was then explored using various 2-bromobenzamides (0.20 mmol). In a typical procedure, a dry reaction tube was charged with a magnetic stir bar, Ni(acac)_2_ (10 mol%), Zn powder (2.0 eq.), PCy_3_ (20 mol%), Cs_2_CO_3_ (2.0 eq.), toluene (1.0 mL), and the corresponding 2-bromobenzamide (0.20 mmol). The tube was evacuated and backfilled with argon, then heated in a 150 °C oil bath for 12 h. After cooling to room temperature, saturated brine was added, and the mixture was extracted with ethyl acetate (20 mL × 3). The combined organic layers were dried over anhydrous Na_2_SO_4_, concentrated under reduced pressure, and purified by silica gel column chromatography to afford the target compound, whose yield was calculated.

#### 3.2.4. Separation and Characterization of Samples

^1^H NMR, ^13^C NMR, and ^19^F NMR data were collected on a Bruker Avance III HD 400 MHz NMR spectrometer (Bruker Corporation, Berlin, Germany). For ^1^H NMR, TMS (δ 0.00) or CDCl_3_ (δ 7.26) was used as the internal standard, while CDCl_3_ (δ 77.00) served as the internal standard for ^13^C NMR. Infrared spectra were recorded using a Bio-Rad FTS-185 spectrometer. HRMS were recorded using a GC-MSQ P2010 gas chromatograph-mass spectrometer. Melting points were measured with a WRS-2 digital melting point apparatus. Column chromatography was performed using 300–400 mesh silica gel. The Supplementary Materials comprehensively present the ^1^H, ^13^C, and ^19^F NMR spectra, along with 16 distinct sets of HRMS and IR data.

## 4. Conclusions

This study developed an efficient nickel-catalyzed intermolecular cyclization strategy for constructing 6(5H)-phenanthridinones from 2-bromobenzamide derivatives. The optimal conditions employed Ni(acac)_2_ (10 mol%), PCy_3_ (20 mol%), Cs_2_CO_3_ (2.0 eq.) in toluene at 150 °C under argon for 12 h, affording the model product in 88% yield. Substrate scope studies demonstrated excellent generality. Nitrogen substituents (13 examples), including alkyl, hydroxyalkyl, benzyl, and aryl groups, were well tolerated (51–92% yields). Phenyl ring substituents (8 examples) such as halogens, nitro, alkyl, and alkoxy groups also performed well (64–86% yields). Electronic effects (electron-donating groups favored) and steric hindrance (6-substitution reduced yields) significantly influenced efficiency. Gram-scale synthesis (10 mmol) afforded 85% yield (1.49 g). Reducing catalyst loading to 5 mol% maintained 85% yield, while 1 mol% dropped the yield to 53%. The key innovation is the first use of inexpensive nickel catalysts to replace precious metals (Pd, Rh) for phenanthridinone synthesis. This method features mild conditions, broad substrate scope, excellent functional group tolerance, operational simplicity, and scalability—offering a green, economical route to bioactive natural products and pharmaceuticals containing the phenanthridinone core.

## Figures and Tables

**Table 1:** Catalyst Screening.

Entry	1	2	3	4	5	6	7	8
**Catalyst (5 mol%)**	CuI	PdCl_2_	Pd(OAc)_2_	NiCl_2_·6H_2_O/Zn	Ni(COD)_2_/Zn	NiBr_2_/Zn	Ni(acac)_2_/Zn	/
**Yield (%)**	NR	24	35	31	42	32	46	NR

Note: The reaction was carried out in toluene with a 0.1 M substrate concentration, 5 mol% catalyst, 1.5 equivalents of zinc powder, 10 mol% PPh_3_, and 2 equivalents of Na_2_CO_3_ at 150 °C for 12 h under inert conditions.

**Table 2:** Ligand Screening.

Entry	1	2	3	4	5	6
**Ligand (10 mol%)**	PPh_3_	dppp	BINOL	BINAP	PCy_3_	/
**Yield (%)**	64	68	62	65	78	27

Note: The reaction was carried out in toluene with a 0.1 M substrate concentration, 5 mol% Ni(acac)_2_, 1.5 equivalents of zinc powder, 10 mol% ligand, and 2 equivalents of Na_2_CO_3_ at 150 °C for 12 h under inert conditions.

**Table 3:** Solvent Screening.

Entry	1	2	3	4	5
**Solvent**	Toluene	1,2-DCE	CH_3_CN	DMSO	DMF
**Yield (%)**	78	47	25	NR	NR

Note: The reaction was carried out in a solvent with 0.1 M substrate, 5 mol% Ni(acac)_2_, 1.5 equivalents of zinc powder, 10 mol% PCy_3_, and 2 equivalents of Na_2_CO_3_ at 150 °C for 12 h under inert conditions.

**Table 4:** Bases Screening.

Entry	1	2	3	4	5	6
**Bases (2 eq.)**	KOAc	KOtBu	NaOH	NEt_3_	K_2_CO_3_	Cs_2_CO_3_
**Yield (%)**	82	80	69	NR	85	88

Note: The reaction was carried out in toluene with a 0.1 M substrate concentration, 5 mol% Ni(acac)_2_, 1.5 equivalents of zinc powder, 10 mol% PCy_3_, and 2 equivalents of base at 150 °C for 12 h under inert conditions.

**Table 5:** Temperature Screening.

Entry	1	2	3	4	5	6
**Temperature (°C)**	90	110	130	150	170	190
**Yield (%)**	47	63	72	78	70	61

Note: The reaction was carried out in toluene with a 0.1 M substrate concentration, 5 mol% Ni(acac)_2_, 1.5 equivalents of zinc powder, 10 mol% PCy_3_, and 2 equivalents of Na_2_CO_3_ at various temperatures for 12 h under inert conditions.

**Table 6:** Summary of Phenanthridinone Derivatives Synthesized via Substrate Scope Expansion.

Entry	Substrate	Product	Yield (%)
1	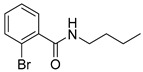	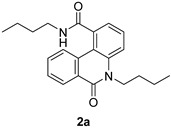	88
2	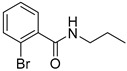	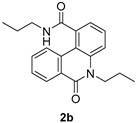	89
3	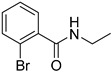	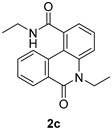	92
4	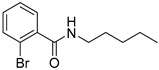	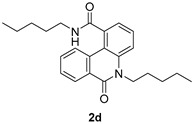	75
5	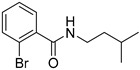	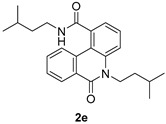	79
6	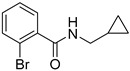	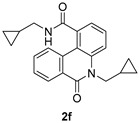	58
7	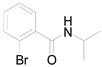	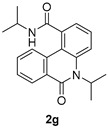	69
8	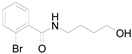	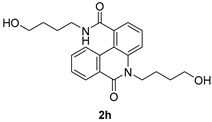	68
9	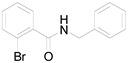	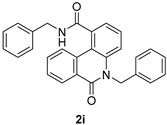	73
10	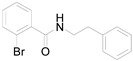	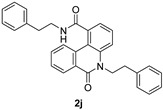	84
11	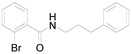	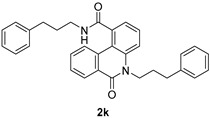	89
12	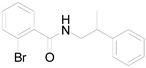	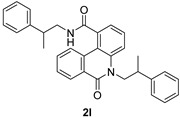	80
13	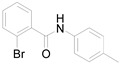	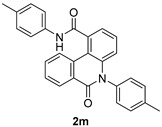	51

**Table 7:** Effect of Substituent Position on the Yields of Phenanthridinone Derivatives.

Entry	Substrate	Product	Yield(%)
14	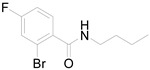	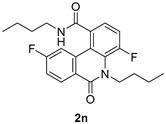	78
15	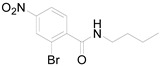	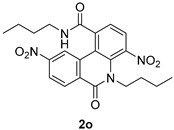	65
16	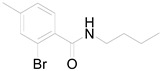	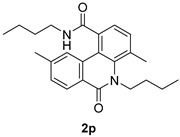	84
17	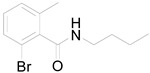	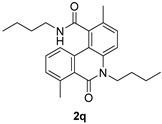	77
18	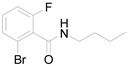	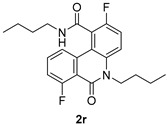	64
19	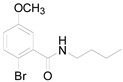	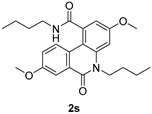	85
20	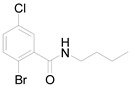	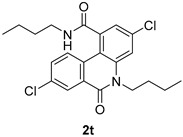	83
21	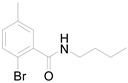	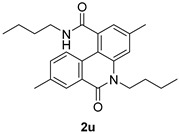	86

## Data Availability

Data are contained within the article and Supplementary Materials.

## Supplementary Materials

The following supporting information can be downloaded at: https://www.mdpi.com/article/10.3390/molecules31071176/s1, Table S1: Main raw materials and reagent specifications. ^1^H, ^13^C, and ^19^F NMR Spectra & Data, HRMS and IR Data.
