# Supplementary material for: Nickel-Catalyzed Intermolecular Cyclization of 2-Bromobenzamide: A General Strategy for Synthesizing 6(5H)-Phenanthridinone Derivatives
Source: Molecules. 2026 Apr 2;31(7):1176. doi: 10.3390/molecules31071176 (PMC13074239; doi:10.3390/molecules31071176)
Supplement: Supplementary file 1 [file molecules-31-01176-s001.zip › molecules-4164623-supplementary.pdf]

Table S1 Main raw materials and reagent specifications

Name	Chemical Formula	Purity	Manufacturer	Address
Cuprous Iodide	CuI	99%	Bide Medicine	Shanghai, China
Palladium Chloride	$PdCl_2$	99%	Bide Medicine	Shanghai, China
Palladium Acetate	$Pd(OAc)_2$	98%	Bide Medicine	Shanghai, China
Nickel Chloride Hexahydrate	$NiCl_2 \cdot 6H_2O$	98%	Adamas	Shanghai, China
Nickel di (cyclooctyl 1, 5-diene)	$Ni(COD)_2$	97%	Bide Medicine	Shanghai, China
Nickel Bromide	$NiBr_2$	96%	Bide Medicine	Shanghai, China
Nickel Acetylacetonate	$Ni(acac)_2$	97%	Bide Medicine	Shanghai, China
Zinc	Zn	99%	Bide Medicine	Shanghai, China
Triphenylphosphine	PPh_3	99%	Adamas	Shanghai, China
1, 3-bis (diphenylphosphino) propane	dppp	97%	Bide Medicine	Shanghai, China
1,1'-bi-2-naphthol	BINOL	98%	Bide Medicine	Shanghai, China
2,2'-bis(diphenylphosphino)-1,1'-binaphthyl	BINAP	97%	Bide Medicine	Shanghai, China
Tricyclohexylphosphine	PCy_3	97%	Bide Medicine	Shanghai, China
Toluene	$C_6H_5CH_3$	99%	Adamas	Shanghai, China
1, 2-dichloroethane	1,2-DCE	A.R.	Adamas	Shanghai, China
Acetonitrile	CH_3CN	99%	Adamas	Shanghai, China
Dimethyl Sulfoxide	DMSO	99%	Adamas	Shanghai, China
Dimethyl Formamide	DMF	A.R.	Bide Medicine	Shanghai, China
Potassium Acetate	KOAc	99%	Bide Medicine	Shanghai, China
Potassium Tert-Butoxide	KOtBu	98%	Bide Medicine	Shanghai, China
Sodium Hydroxide	NaOH	96%	Adamas	Shanghai, China

Triethylamine	NEt_3	96%	Adamas	Shanghai, China
Potassium Carbonate	K_2CO_3	99%	Bide Medicine	Shanghai, China
Cesium Carbonate	Cs_2CO_3	98%	Bide Medicine	Shanghai, China
2-bromobenzoyl butylamine	$C_{11}H_{14}BrNO$	99%	Adamas	Shanghai, China
Sodium Carbonate	Na_2CO_3	99%	Bide Medicine	Shanghai, China
Ethyl Acetate	$CH_3COOC_2H_5$	A.R.	Bide Medicine	Shanghai, China
Anhydrous Sodium Sulfate	Na_2SO_4	A.R.	Bide Medicine	Shanghai, China
2-Bromobenzoic Acid	$C_7H_5BrO_2$	99%	Adamas	Shanghai, China
Thionyl Chloride	$SOCl_2$	99%	Adamas	Shanghai, China
Dichloromethane	DCM	99%	Adamas	Shanghai, China

^{1}H and ^{13}C NMR spectra of compound **2a**.

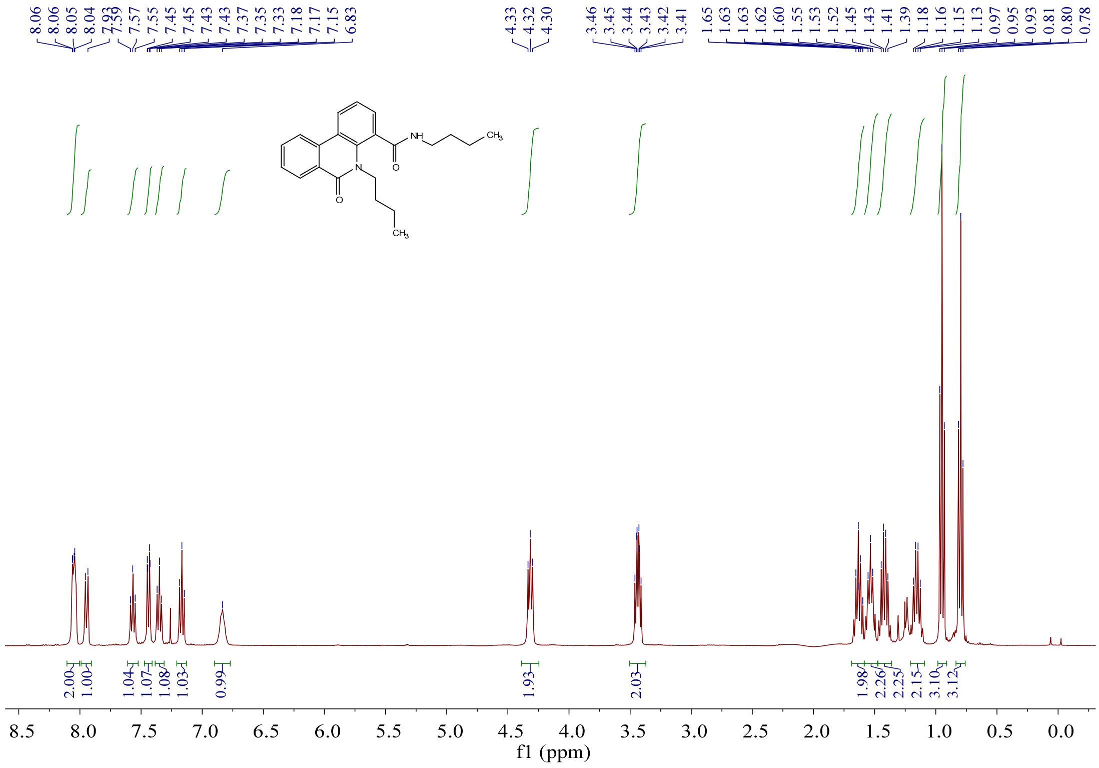

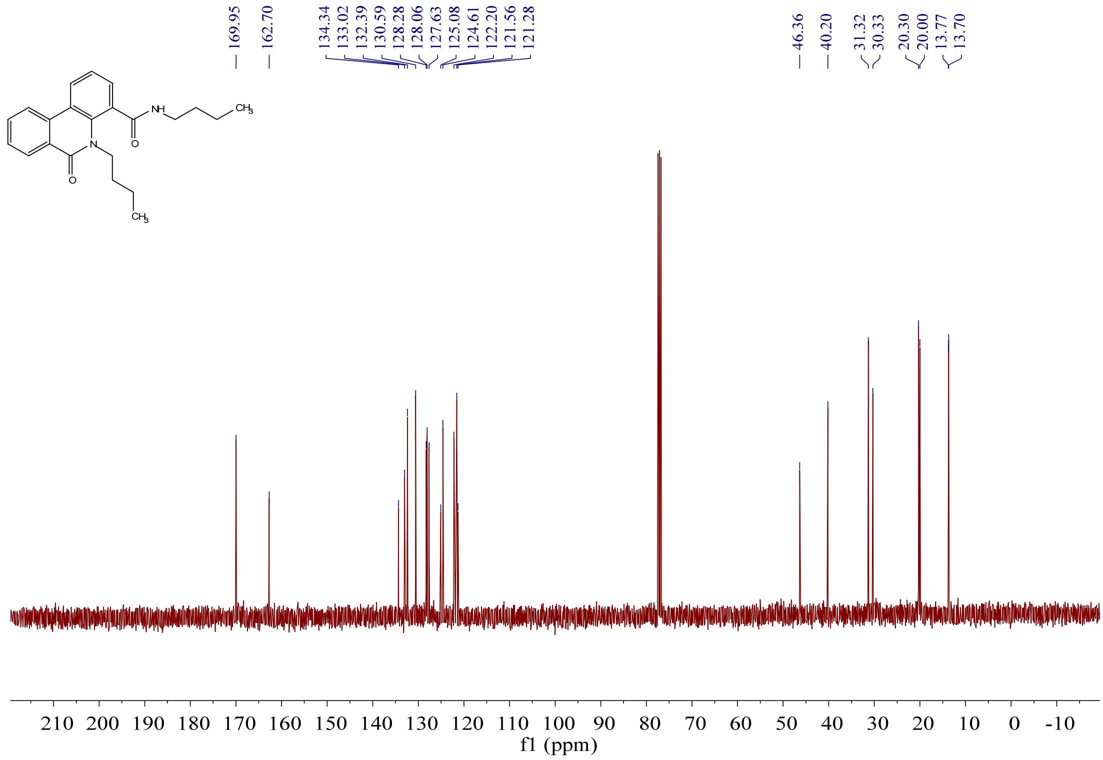

HRMS spectra of compound **2a**.

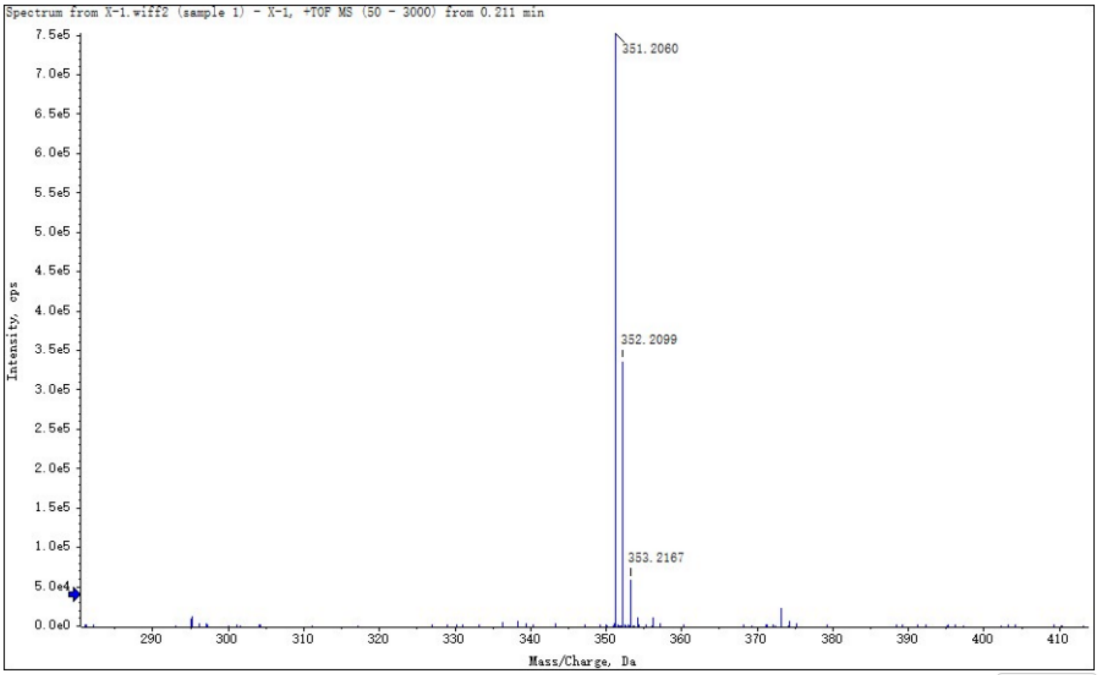

IR spectra of compound **2a**.

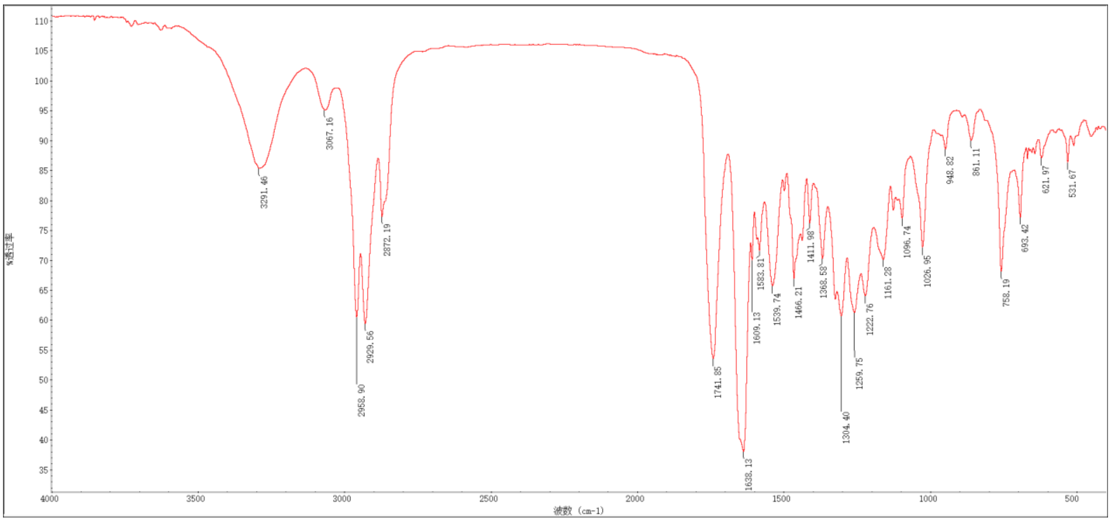

N,5-dibutyl-6-oxo-5,6-dihydrophenanthridine-4-carboxamide (2a):

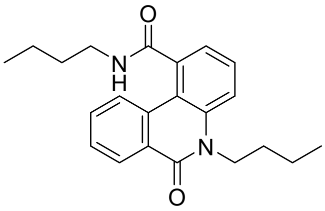

2a

White solid, yield 88%; **^{1}H NMR** (400 MHz, $CDCl_3$) δ 8.05 (dd, *J* = 6.3, 1.5 Hz, 2H), 7.94 (d, *J* = 8.2 Hz, 1H), 7.57 (t, *J* = 7.6 Hz, 1H), 7.44 (dd, *J* = 7.4, 1.3 Hz, 1H), 7.35 (t, *J* = 7.6 Hz, 1H), 7.17 (t, *J* =

7.7 Hz, 1H), 6.83 (s, 1H), 4.39 – 4.25 (m, 2H), 3.44 (dt, *J* = 7.3, 5.9 Hz, 2H), 1.69 – 1.59 (m, 2H), 1.53 (t, *J* = 7.6 Hz, 2H), 1.42 (dd, *J* = 15.1, 7.4 Hz, 2H), 1.15 (dd, *J* = 15.2, 7.5 Hz, 2H), 0.95 (t, *J* = 7.4 Hz, 3H), 0.80 (t, *J* = 7.4 Hz, 3H). **^{13}C NMR** (100 MHz, $CDCl_3$) δ 169.95, 162.70, 134.34, 133.02, 132.39, 130.59, 128.28, 128.06, 127.63, 125.08, 124.61, 122.20, 121.56, 121.28, 46.36, 40.20, 31.32, 30.33, 20.30, 20.00, 13.77, 13.70. **HRMS** (ESI-TOF) m/z: $[M+H]^+$ calcd for $C_{22}H_{27}N_2O_2$ 351.2067; found 351.2060. **IR** (KBr): 3291, 3067, 2959, 2930, 2872, 1742, 1638, 1609, 1584, 1540, 1466, 1412, 1369, 1304, 1260, 1223, 1161, 1097, 1027, 949, 861, 758, 693, 622, 532 cm^{-1}.

^{1}H and ^{13}C NMR spectra of compound **2b**.

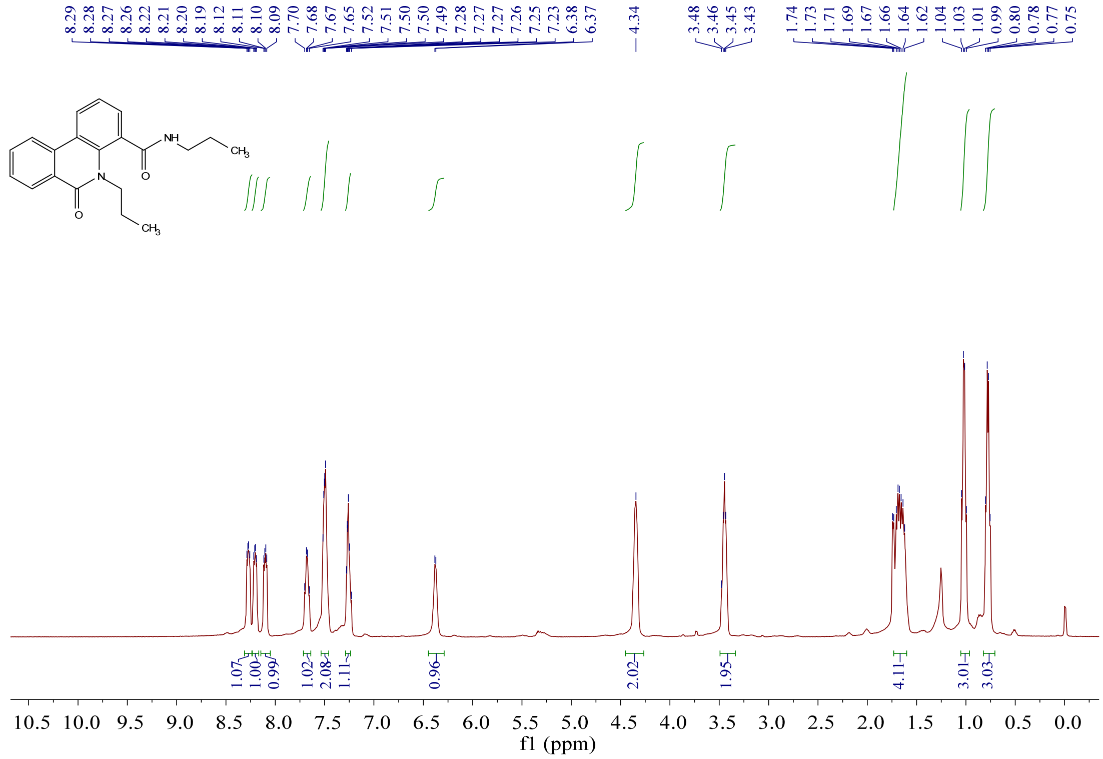

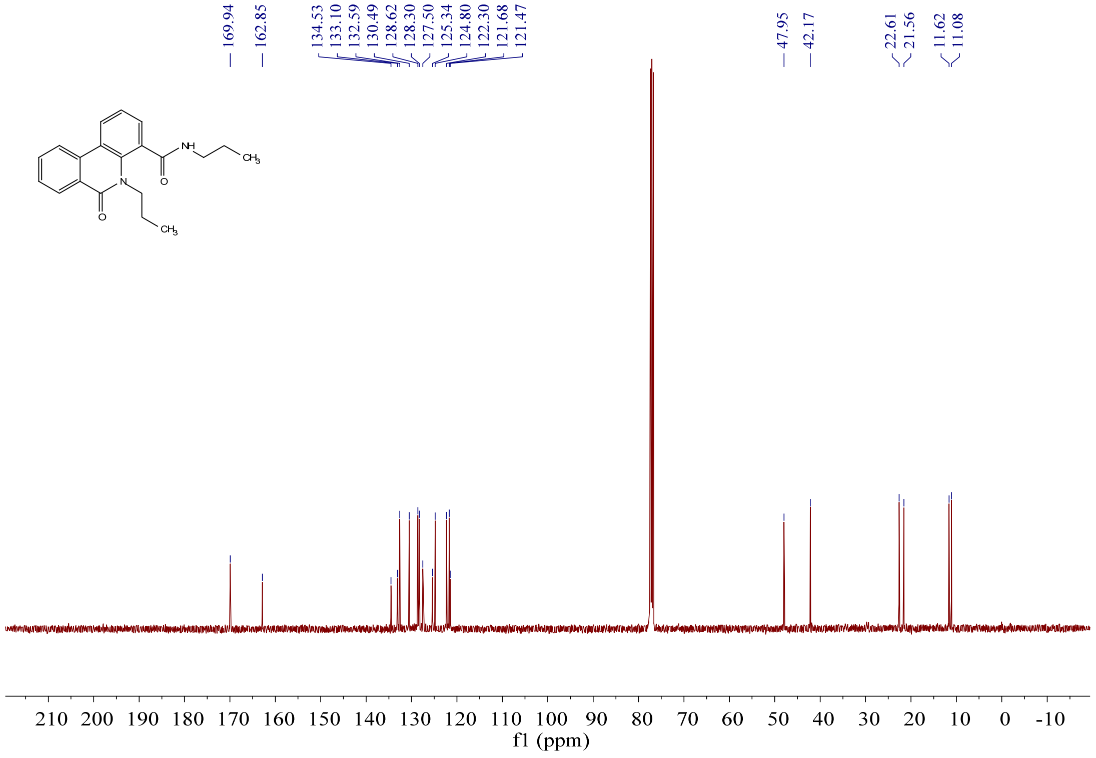

HRMS spectra of compound **2b**.

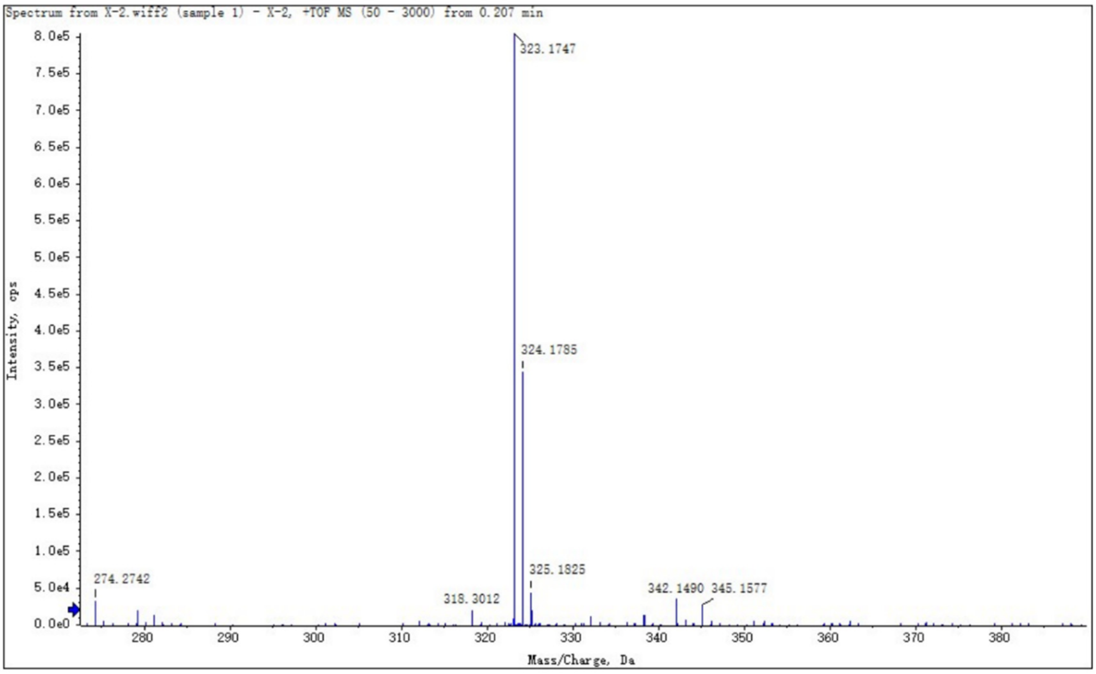

IR spectra of compound **2b**.

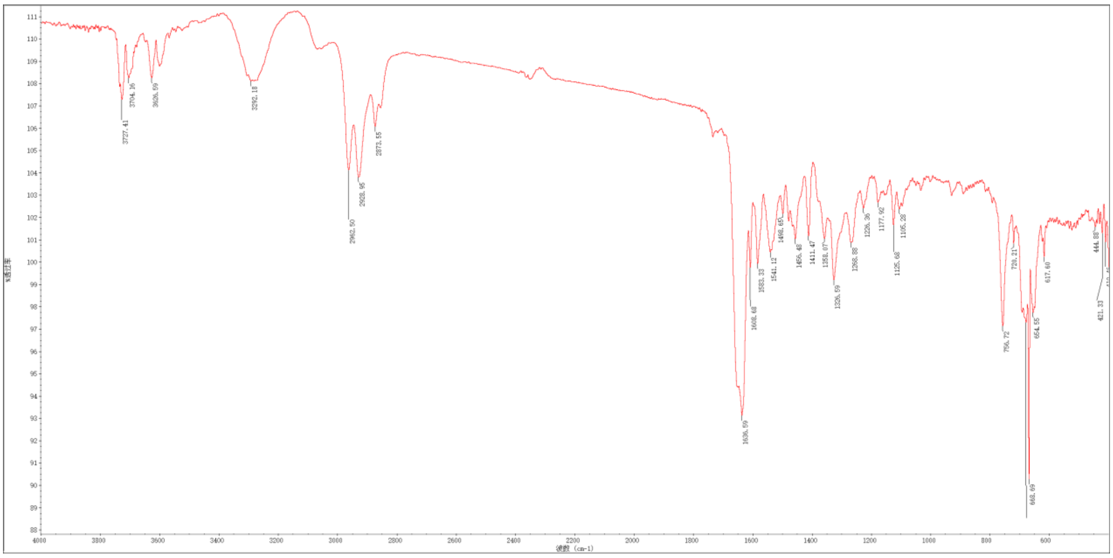

N,5-dipropyl-6-oxo-5,6-dihydrophenanthridine-4-carboxamide (2b):

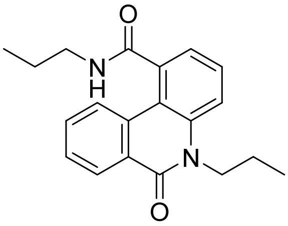

2b

White solid, yield 89%, m.p.130-132°C; **^{1}H NMR** (400 MHz, $CDCl_3$) δ 8.27 (dd, *J* = 7.0, 4.4 Hz, 1H), 8.20 (dd, *J* = 7.0, 4.3 Hz, 1H), 8.10 (dd, *J* = 7.8, 4.3 Hz, 1H), 7.68 (dd, *J* = 11.4, 7.5 Hz, 1H), 7.54 – 7.46 (m, 2H), 7.29 – 7.24 (m, 1H), 6.38 (d, *J* = 3.4 Hz, 1H), 4.34 (s, 2H), 3.46 (dd, *J* = 11.5, 6.2 Hz, 2H), 1.73 – 1.60 (m, 4H), 1.02 (dd, *J* = 12.3, 7.2 Hz, 3H), 0.78 (dd, *J* = 12.0, 6.9 Hz, 3H). **^{13}C NMR** (101 MHz, $CDCl_3$) δ 169.94, 162.85, 134.53, 133.10, 132.59, 130.49, 128.62, 128.30, 127.50, 125.34, 124.80, 122.30, 121.68, 121.47, 47.95, 42.17, 22.61, 21.56, 11.62, 11.08. **HRMS** (ESI-TOF) m/z: $[M+H]^+$ calcd for $C_{20}H_{23}N_2O_2$ 323.1754; found 323.1747. **IR** (KBr): 3727, 3704, 3627, 3292, 2963, 2929, 2874, 1637, 1609, 1583, 1541, 1499, 1456, 1411, 1358, 1327, 1269, 1226, 1178, 1126, 1105, 757, 720, 669, 618, 532 cm^{-1}.

^{1}H and ^{13}C NMR spectra of compound **2c**.

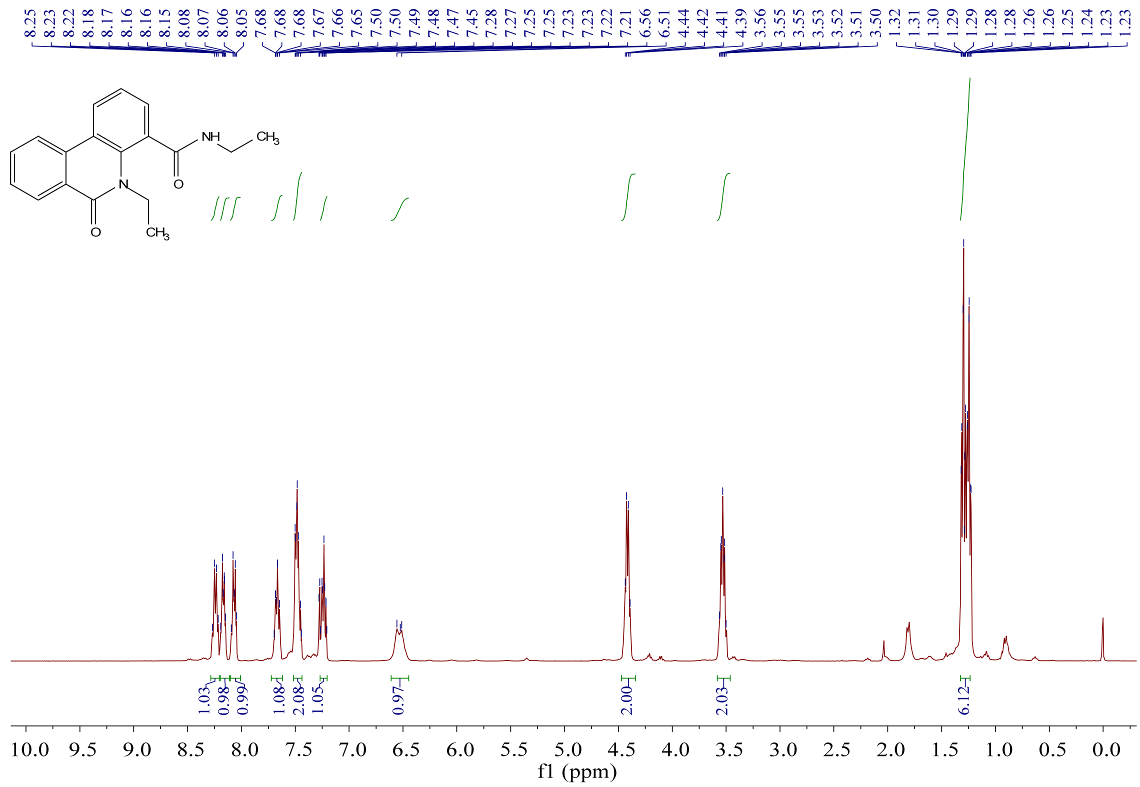

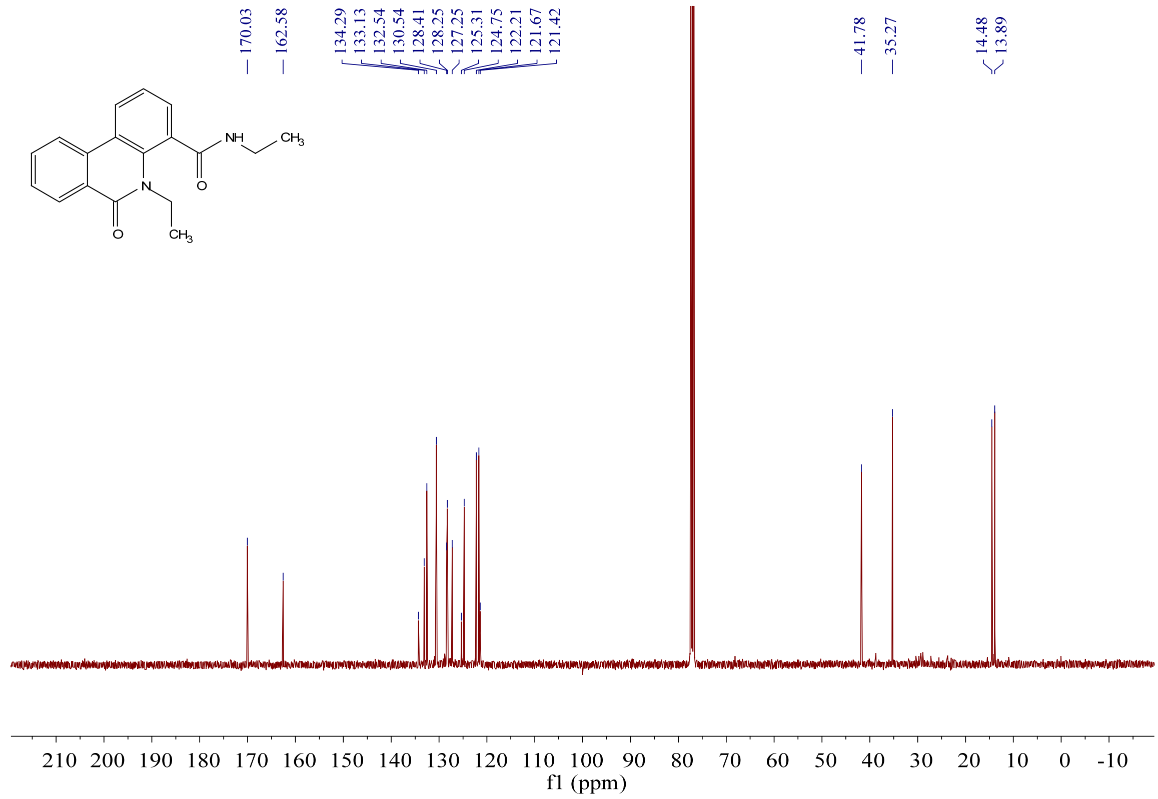

N,5-diethyl-6-oxo-5,6-dihydrophenanthridine-4-carboxamide (2c):

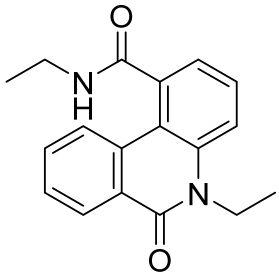

2c

White solid, yield 92%; **^{1}H NMR** (400 MHz, $CDCl_3$) δ 8.28 – 8.21 (m, 1H), 8.20 – 8.11 (m, 1H), 8.10 – 8.01 (m, 1H), 7.72 – 7.62 (m, 1H), 7.52 – 7.44 (m, 2H), 7.27 – 7.20 (m, 1H), 6.61 – 6.45 (m, 1H), 4.41 (dd, J = 12.4, 5.7 Hz, 2H), 3.58 – 3.46 (m, 2H), 1.32 – 1.23 (m, 6H). **^{13}C NMR** (101 MHz, $CDCl_3$) δ 170.03, 162.58, 134.29, 133.13, 132.54, 130.54, 128.41, 128.25, 127.25, 125.31, 124.75, 122.21, 121.67, 121.42, 41.78, 35.27, 14.48, 13.89.

^{1}H and ^{13}C NMR spectra of compound **2d**.

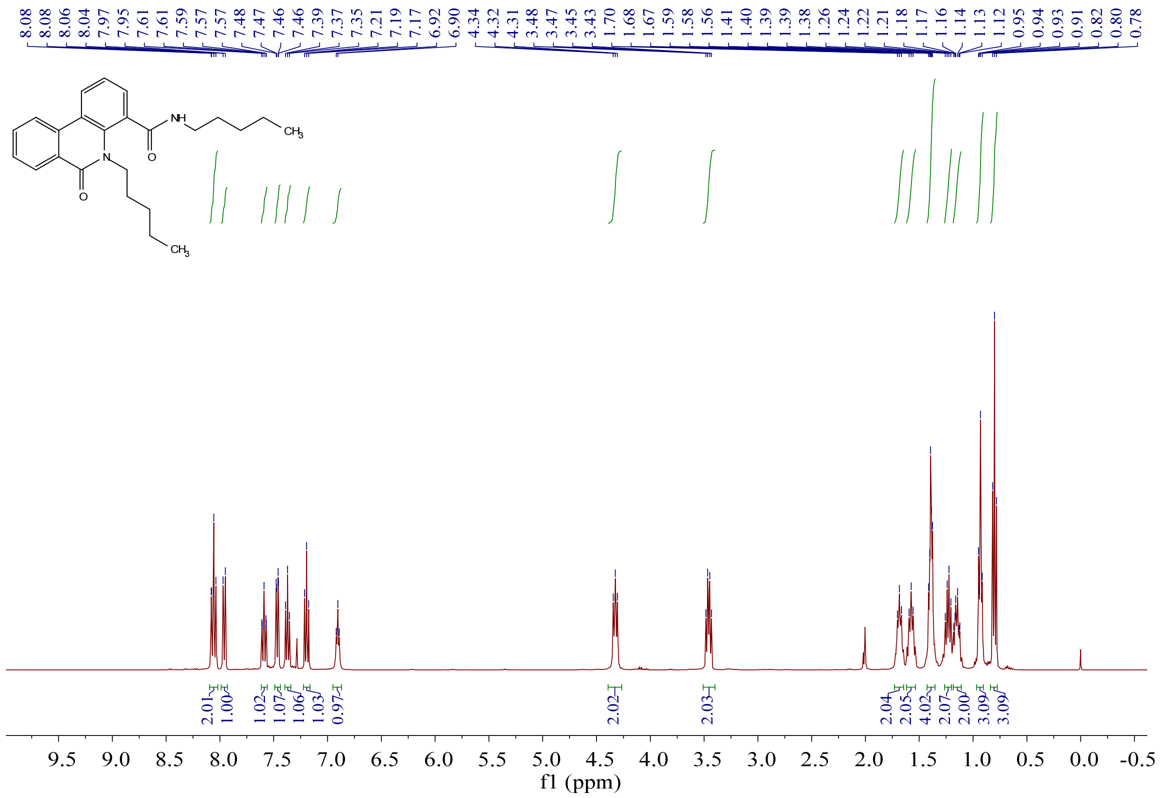

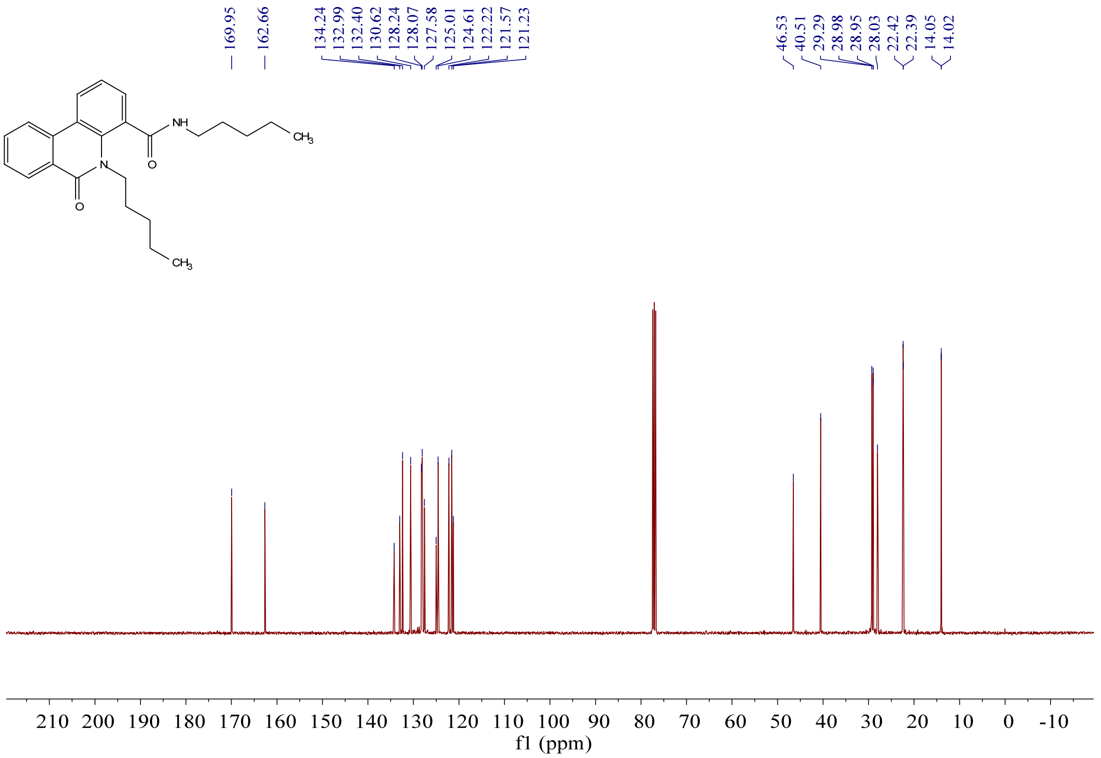

HRMS spectra of compound **2d**.

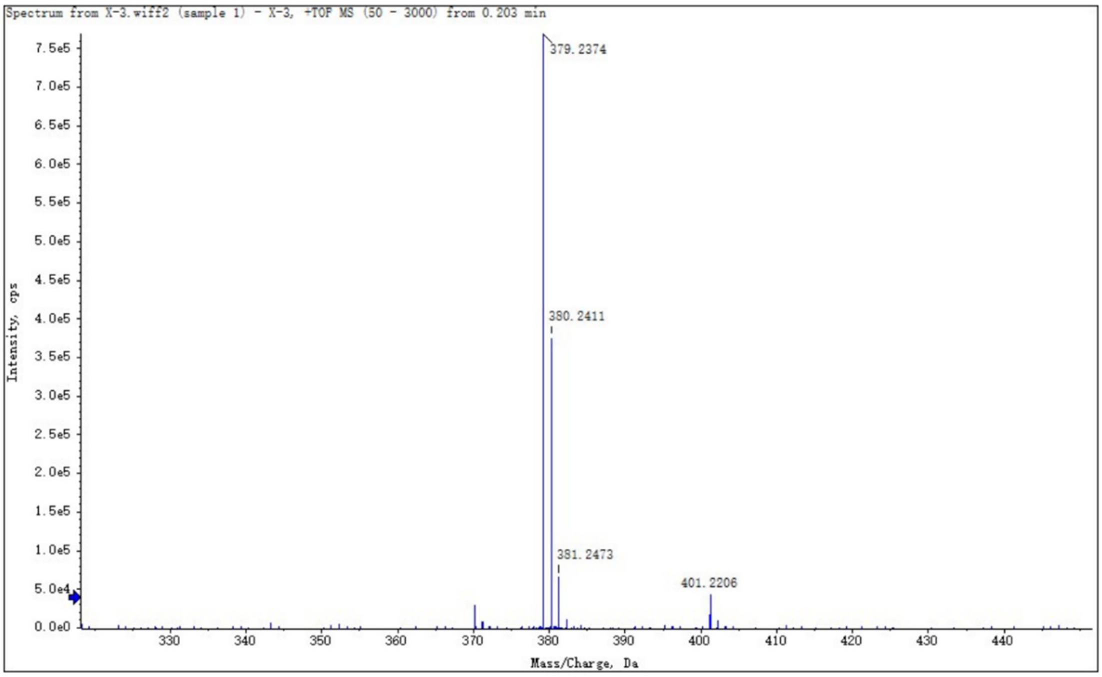

IR spectra of compound **2d**.

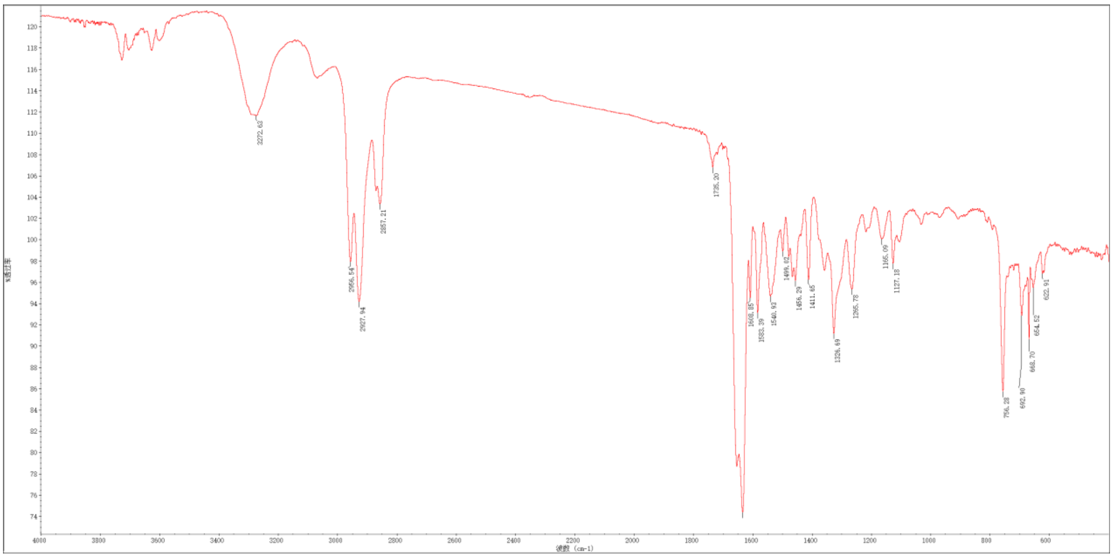

N,5-dipentyl-6-oxo-5,6-dihydrophenanthridine-4-carboxamide (2d):

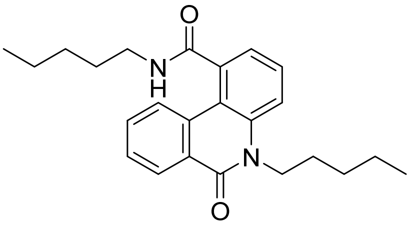

2d

White solid, yield 75%, m.p.117-119°C; **^{1}H NMR** (400 MHz, $CDCl_3$) δ 8.06 (dd, *J* = 12.7, 4.6 Hz, 2H), 7.96 (d, *J* = 8.2 Hz, 1H), 7.62 – 7.56 (m, 1H), 7.47 (dd, *J* = 7.4, 1.2 Hz, 1H), 7.37 (t, *J* = 7.5 Hz, 1H), 7.19 (t, *J* = 7.7 Hz, 1H), 6.90 (t, *J* = 5.5 Hz, 1H), 4.39 – 4.27 (m, 2H), 3.46 (dd, *J* = 14.2, 6.2 Hz, 2H), 1.73 – 1.64 (m, 2H), 1.62 – 1.53 (m, 2H), 1.43 – 1.35 (m, 4H), 1.23 (dd, *J* = 13.9, 6.8 Hz, 2H), 1.15 (td, *J* = 8.0, 4.0 Hz, 2H), 0.93 (dd, *J* = 9.3, 4.5 Hz, 3H), 0.80 (t, *J* = 7.2 Hz, 3H). **^{13}C NMR** (101 MHz, $CDCl_3$) δ 169.95, 162.66, 134.24, 132.99, 132.40, 130.62, 128.24, 128.07, 127.58, 125.01, 124.61, 122.22, 121.57, 121.23, 46.53, 40.51, 29.29, 28.98, 28.95, 28.03, 22.42, 22.39, 14.05, 14.02. **HRMS** (ESI-TOF) m/z: $[M+H]^+$ calcd for $C_{24}H_{31}N_2O_2$ 379.2380; found 379.2374. **IR** (KBr): 3273, 2957, 2928, 2857, 1735, 1609, 1583, 1541, 1499, 1456, 1412, 1327, 1266, 1165, 1127, 756, 693, 669, 655, 623, 532 cm^{-1}.

^{1}H and ^{13}C NMR spectra of compound **2e**.

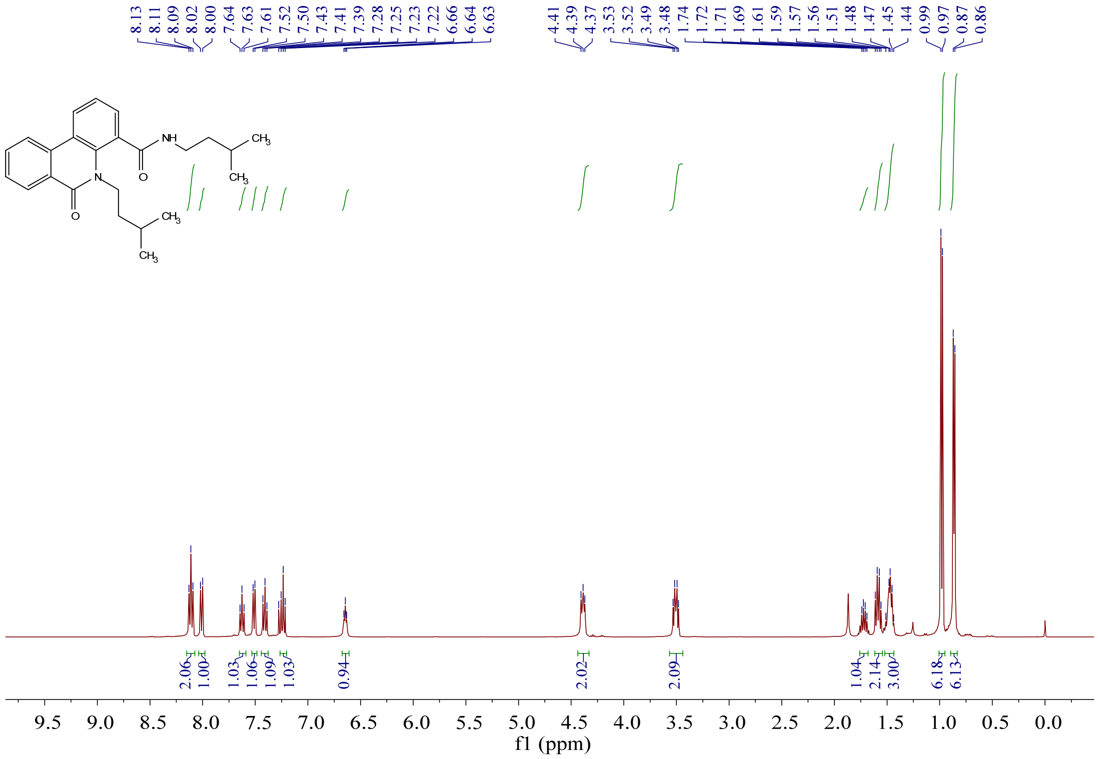

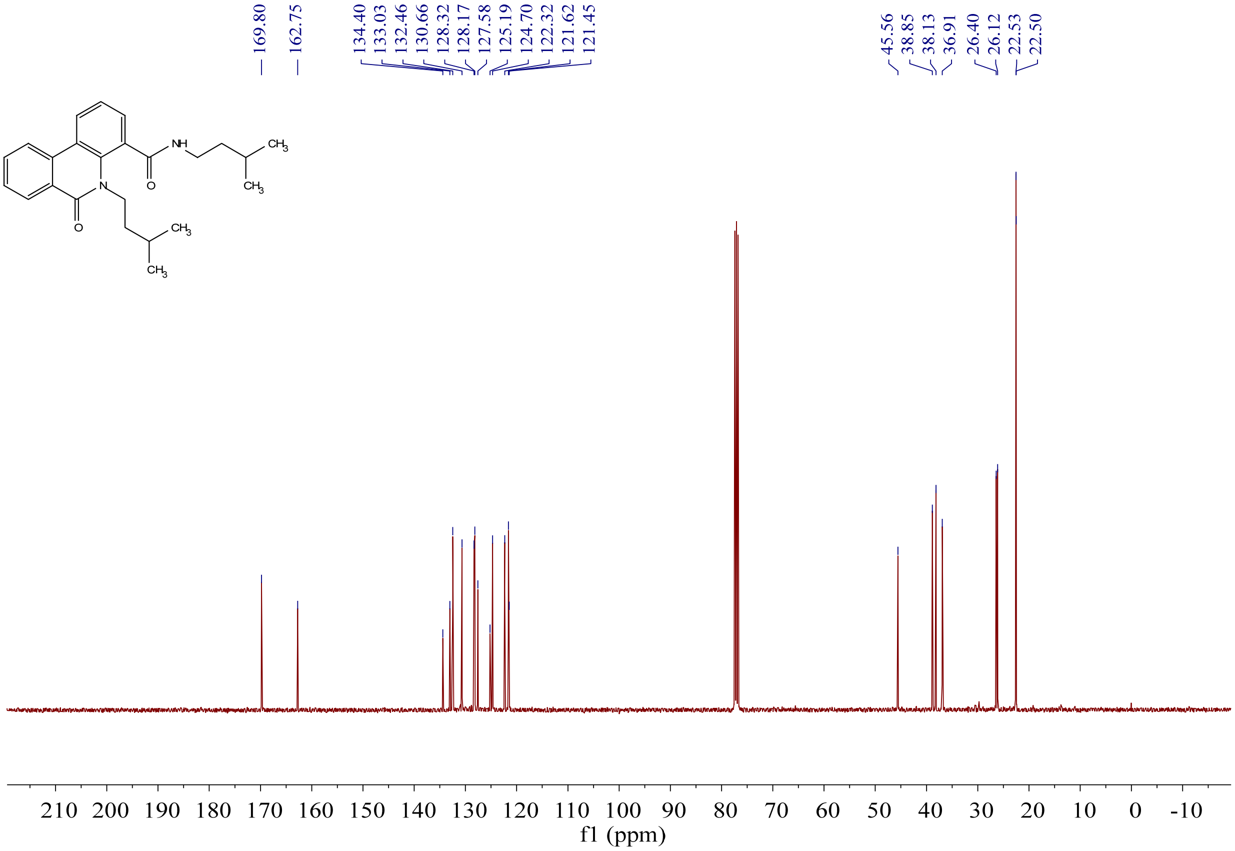

HRMS spectra of compound **2e**.

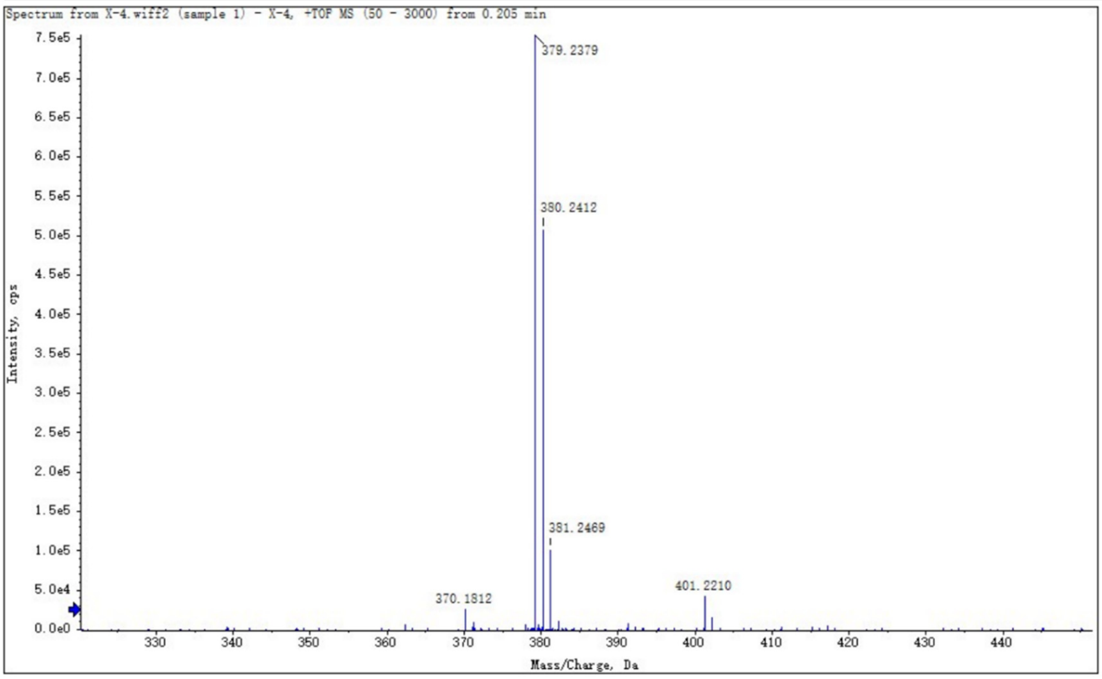

IR spectra of compound **2e**.

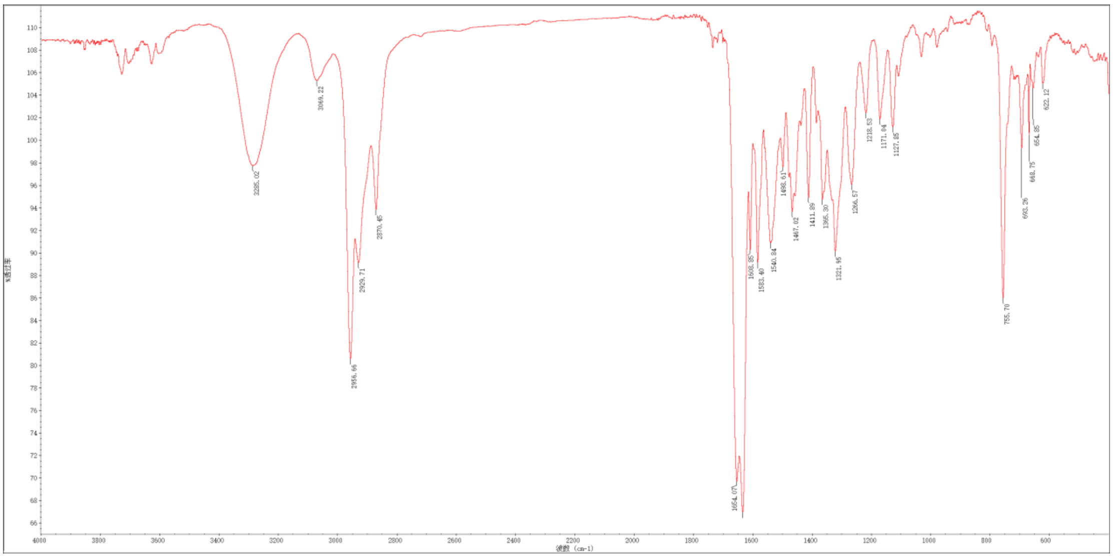

N,5-diisopentyl-6-oxo-5,6-dihydrophenanthridine-4-carboxamide (2e):

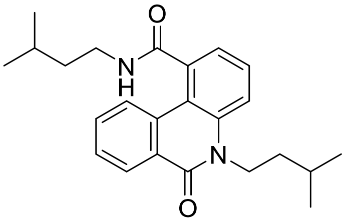

2e

White solid, yield 79%, m.p. 217-219°C; **^{1}H NMR** (400 MHz, $CDCl_3$) δ 8.11 (t, *J* = 7.4 Hz, 2H), 8.01 (d, *J* = 8.2 Hz, 1H), 7.63 (t, *J* = 7.5 Hz, 1H), 7.51 (d, *J* = 6.9 Hz, 1H), 7.41 (t, *J* = 7.6 Hz, 1H), 7.23 (t, *J* = 7.7 Hz, 1H), 6.64 (t, *J* = 5.3 Hz, 1H), 4.44 – 4.33 (m, 2H), 3.51 (dd, *J* = 14.9, 5.9 Hz, 2H), 1.72 (dd, *J* = 13.3, 6.6 Hz, 1H), 1.58 (dd, *J* = 15.1, 7.1 Hz, 2H), 1.52 – 1.44 (m, 3H), 0.98 (d, *J* = 6.6 Hz, 6H), 0.86 (d, *J* = 6.0 Hz, 6H). **^{13}C NMR** (101 MHz, $CDCl_3$) δ 169.80, 162.75, 134.40, 133.03, 132.46, 130.66, 128.32, 128.17, 127.58, 125.19, 124.70, 122.32, 121.62, 121.45, 45.56, 38.85, 38.13, 36.91, 26.40, 26.12, 22.53, 22.50. **HRMS** (ESI-TOF) m/z: $[M+H]^+$ calcd for $C_{24}H_{31}N_2O_2$ 379.2380; found 379.2379. **IR** (KBr): 3285, 3069, 2957, 2930, 2870, 1654, 1609, 1583, 1541, 1499, 1467, 1412, 1365, 1322, 1267, 1219, 1171, 1128, 756, 693, 622, 532 cm^{-1}.

^{1}H and ^{13}C NMR spectra of compound **2f**.

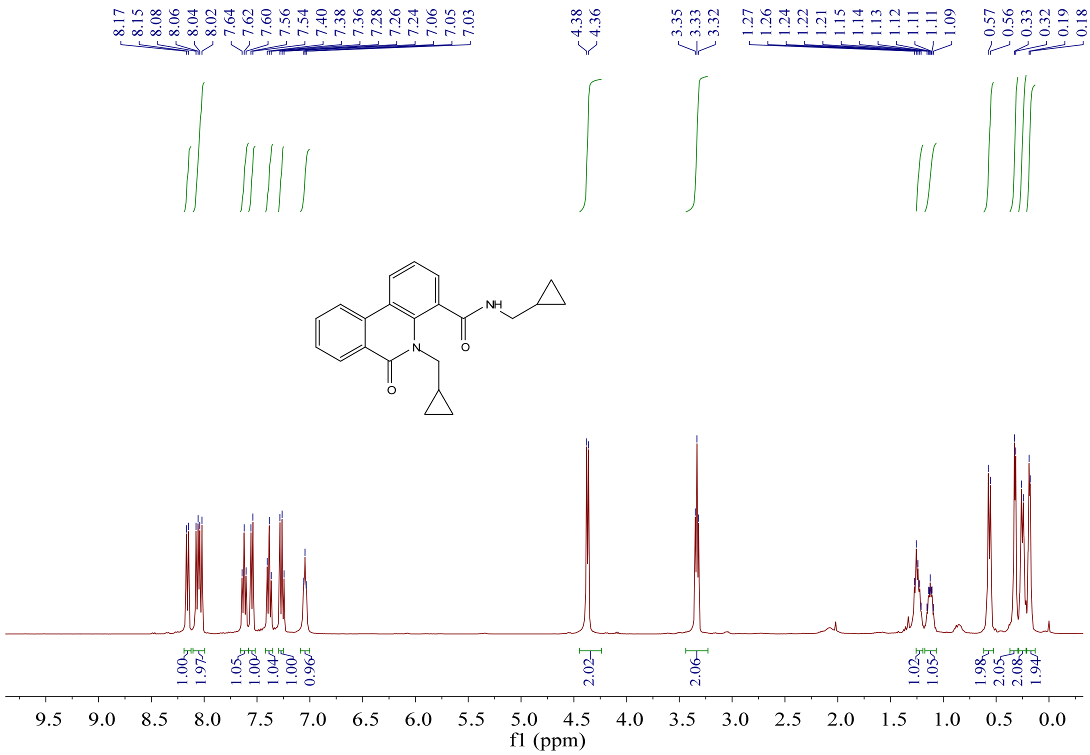

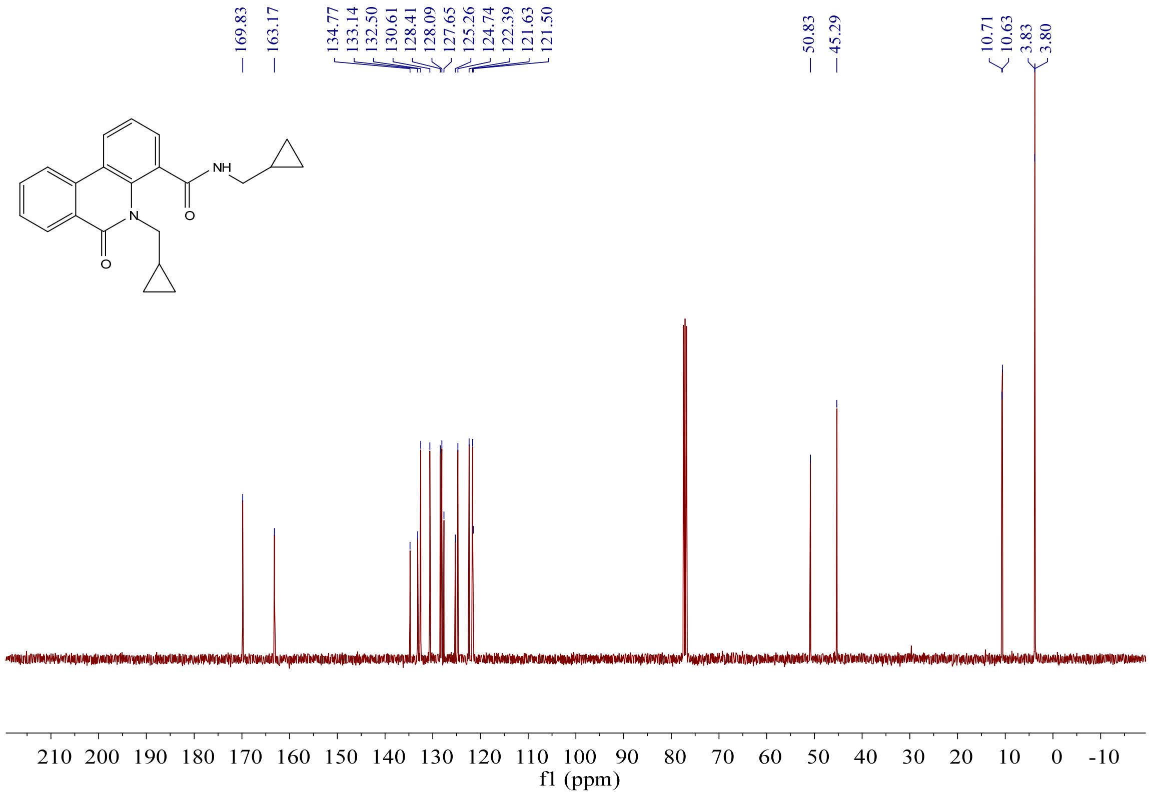

HRMS spectra of compound **2f**.

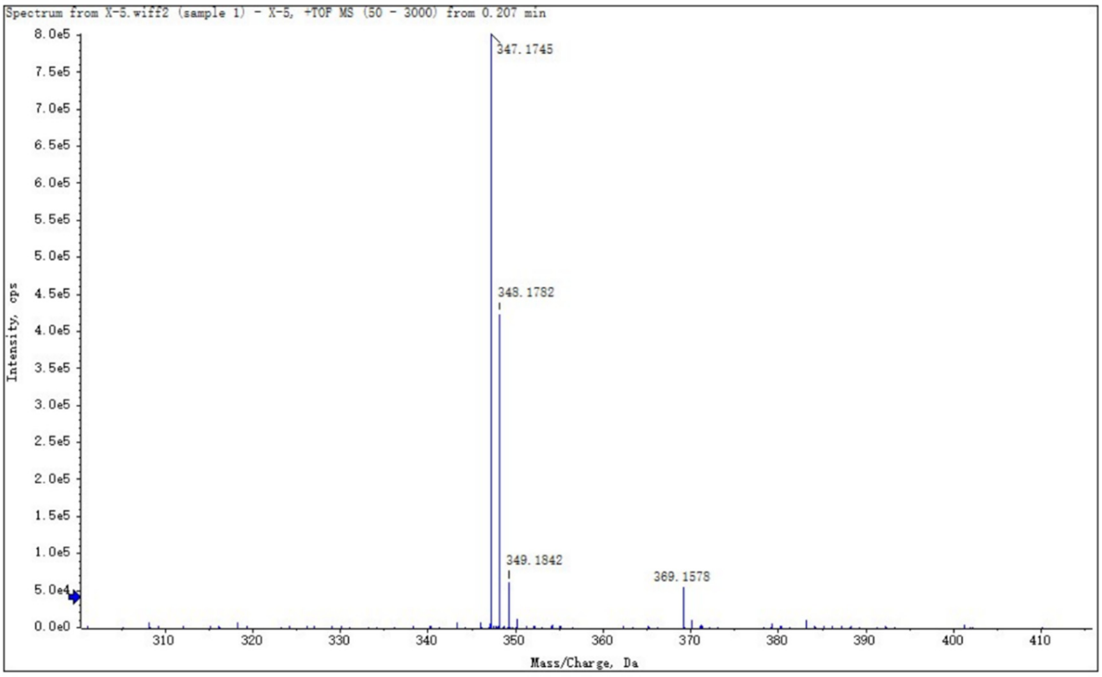

IR spectra of compound **2f**.

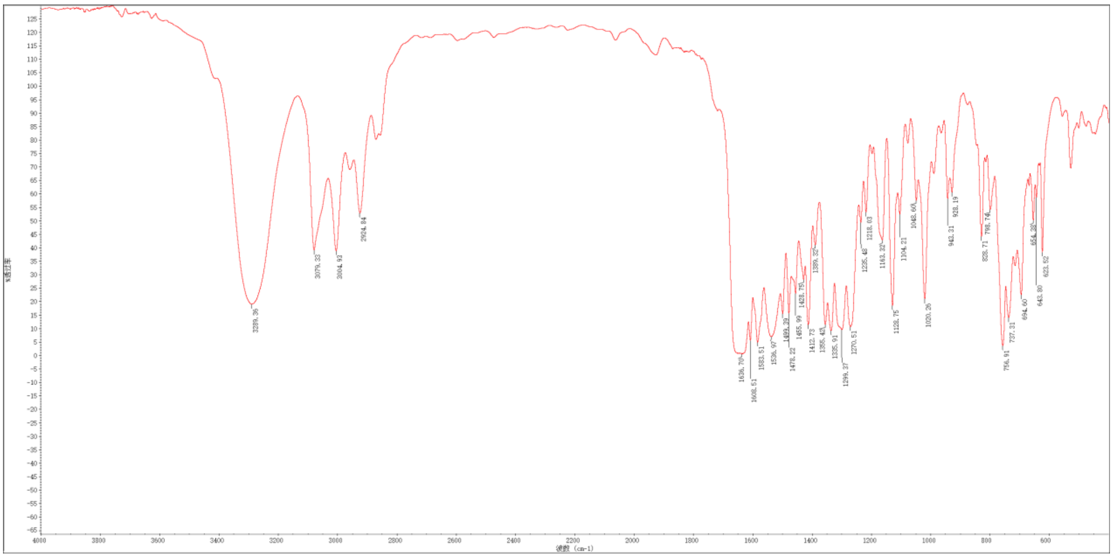

N,5-bis(cyclopropylmethyl)-6-oxo-5,6-dihydrophenanthridine-4-carboxamide (2f):

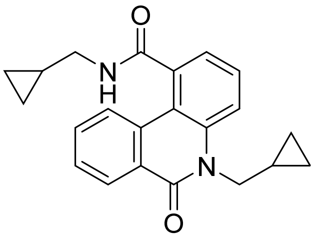

2f

White solid, yield 58%, m.p. 140-142°C; **^{1}H NMR** (400 MHz, $CDCl_3$) δ 8.16 (d, J = 8.0 Hz, 1H), 8.05

(dd, *J* = 14.1, 8.1 Hz, 2H), 7.62 (t, *J* = 7.6 Hz, 1H), 7.55 (d, *J* = 7.4 Hz, 1H), 7.38 (t, *J* = 7.5 Hz, 1H), 7.27 (d, *J* = 8.5 Hz, 1H), 7.05 (t, *J* = 4.8 Hz, 1H), 4.37 (d, *J* = 6.9 Hz, 2H), 3.33 (t, *J* = 6.3 Hz, 2H), 1.23 (dd, *J* = 12.4, 5.9 Hz, 1H), 1.18 – 1.07 (m, 1H), 0.57 (d, *J* = 7.7 Hz, 2H), 0.32 (d, *J* = 4.8 Hz, 2H), 0.25 (d, *J* = 7.8 Hz, 2H), 0.18 (d, *J* = 4.3 Hz, 2H). **^{13}C NMR** (101 MHz, $CDCl_3$) δ 169.83, 163.17, 134.77, 133.14, 132.50, 130.61, 128.41, 128.09, 127.65, 125.26, 124.74, 122.39, 121.63, 121.50, 50.83, 45.29, 10.71, 10.63, 3.83, 3.80. **HRMS** (ESI-TOF) m/z: $[M+H]^+$ calcd for $C_{22}H_{23}N_2O_2$ 347.1754; found 347.1745. **IR** (KBr): 3289, 3079, 3005, 2925, 1637, 1609, 1584, 1537, 1499, 1456, 1413, 1389, 1355, 1299, 1271, 1218, 1129, 1020, 943, 829, 757, 695, 654, 624, 532 cm^{-1}.

^{1}H and ^{13}C NMR spectra of compound **2g**.

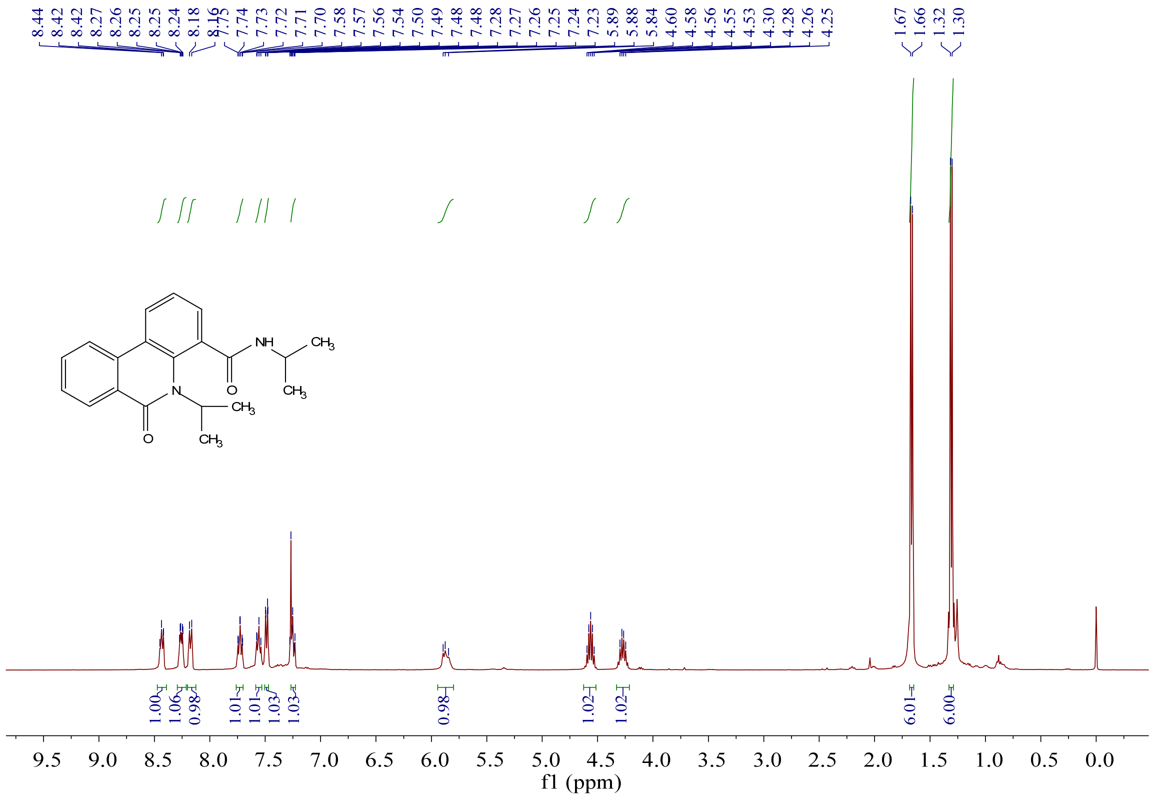

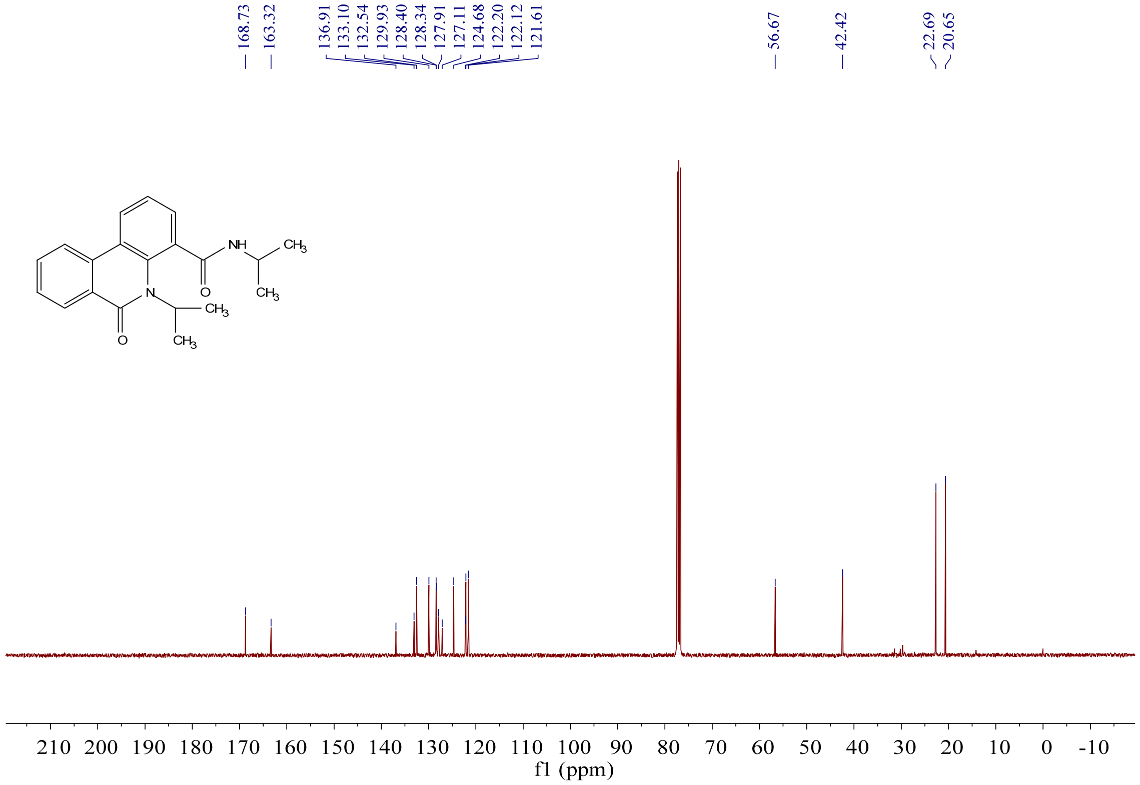

N,5-diisopropyl-6-oxo-5,6-dihydrophenanthridine-4-carboxamide (2g):

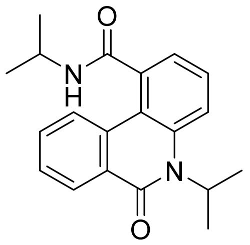

2g

White solid, yield 69%; **^{1}H NMR** (400 MHz, $CDCl_3$) δ 8.43 (dd, *J* = 9.8, 3.3 Hz, 1H), 8.29 – 8.21 (m, 1H), 8.17 (d, *J* = 8.1 Hz, 1H), 7.76 – 7.70 (m, 1H), 7.56 (dd, *J* = 11.1, 4.0 Hz, 1H), 7.49 (dd, *J* = 7.4, 1.5 Hz, 1H), 7.25 (dd, *J* = 7.7, 3.1 Hz, 1H), 5.94 – 5.80 (m, 1H), 4.56 (dt, *J* = 13.0, 6.5 Hz, 1H), 4.27 (dd, *J* = 13.7, 7.0 Hz, 1H), 1.67 (d, *J* = 6.5 Hz, 6H), 1.31 (d, *J* = 6.6 Hz, 6H). **^{13}C NMR** (101 MHz, $CDCl_3$) δ 168.73, 163.32, 136.91, 133.10, 132.54, 129.93, 128.40, 128.34, 127.91, 127.11, 124.68, 122.20, 122.12, 121.61, 56.67, 42.42, 22.69, 20.65.

^{1}H and ^{13}C NMR spectra of compound **2h**.

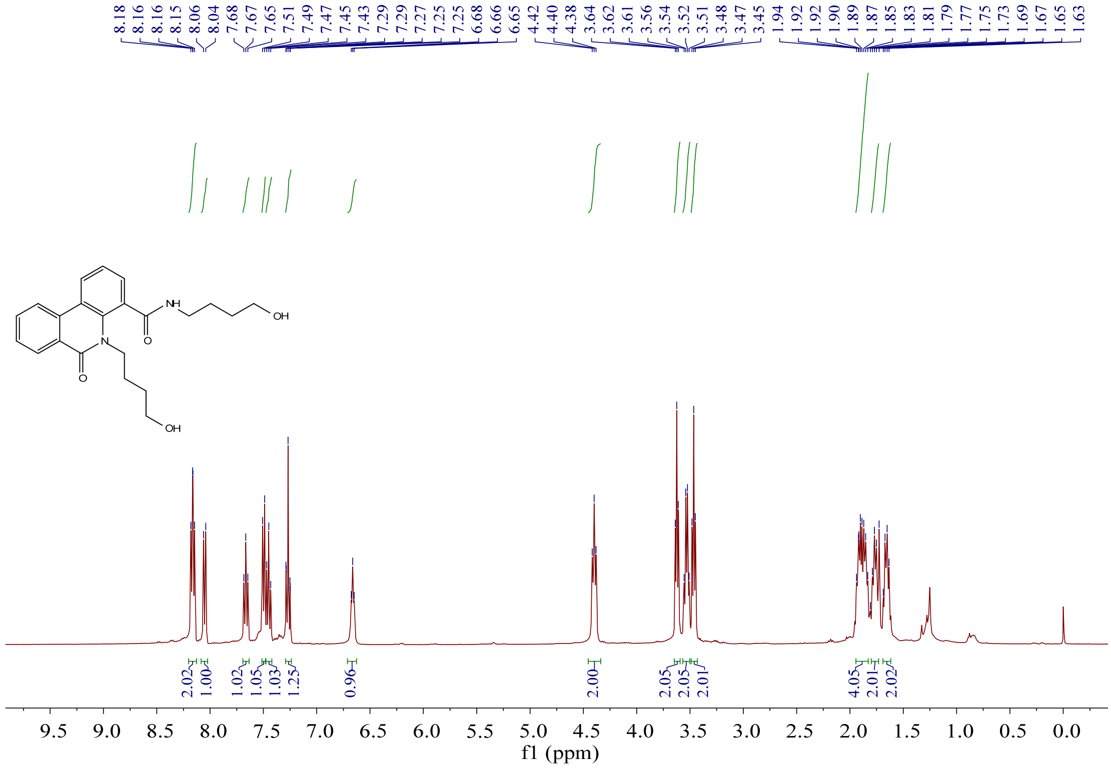

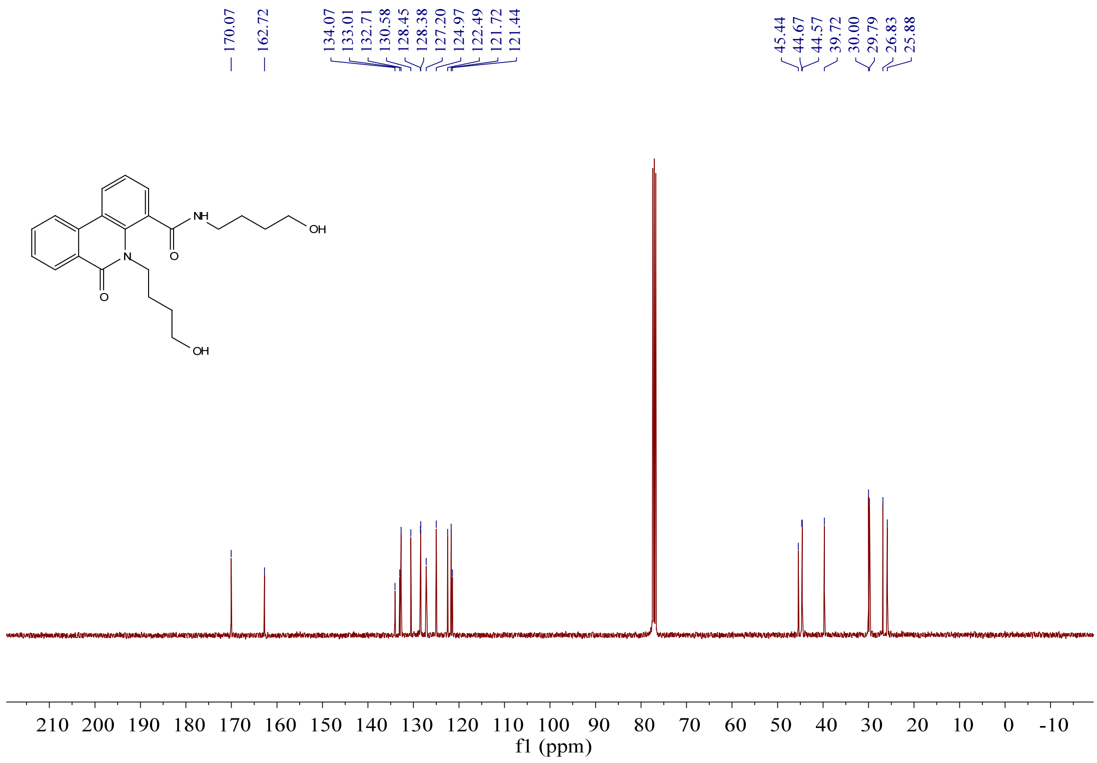

IR spectra of compound **2h**.

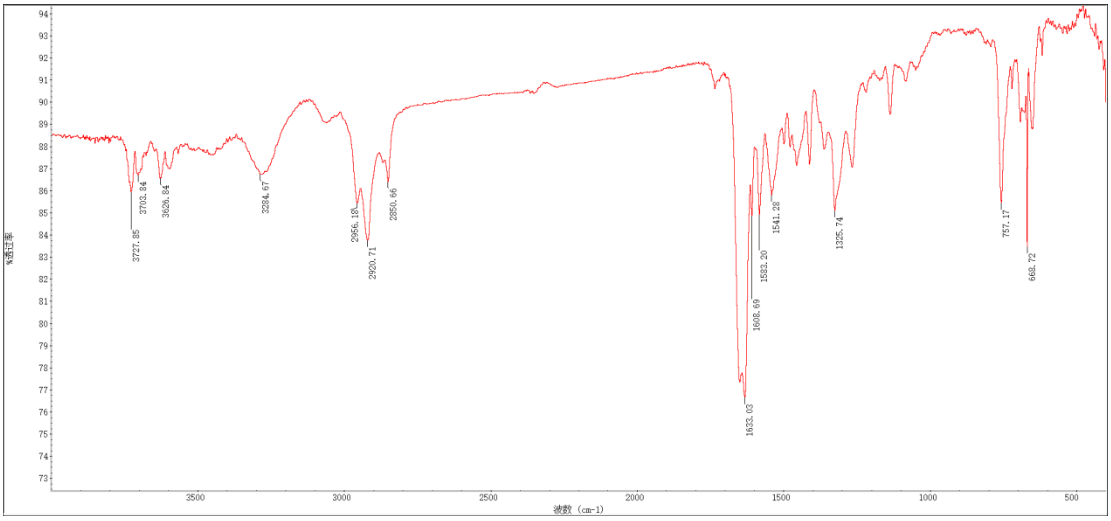

N,5-bis(4-hydroxybutyl)-6-oxo-5,6-dihydrophenanthridine-4-carboxamide (2h):

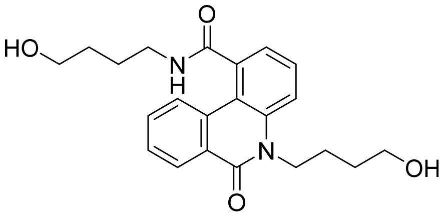

2h

White solid, yield 68%, m.p. 141-143°C; **^{1}H NMR** (400 MHz, $CDCl_3$) δ 8.16 (dd, *J* = 7.5, 6.4 Hz, 2H), 8.05 (d, *J* = 8.2 Hz, 1H), 7.67 (t, *J* = 7.7 Hz, 1H), 7.50 (d, *J* = 7.4 Hz, 1H), 7.45 (t, *J* = 7.6 Hz, 1H), 7.29 – 7.24 (m, 1H), 6.66 (t, *J* = 5.3 Hz, 1H), 4.40 (t, *J* = 7.2 Hz, 2H), 3.62 (t, *J* = 5.6 Hz, 2H), 3.53 (dd, *J* = 12.7, 6.2 Hz, 2H), 3.47 (t, *J* = 6.4 Hz, 2H), 1.89 (ddd, *J* = 21.8, 11.8, 7.4 Hz, 4H), 1.80 – 1.73 (m, 2H), 1.66 (dd, *J* = 14.5, 6.9 Hz, 2H). **^{13}C NMR** (101 MHz, $CDCl_3$) δ 170.07, 162.72, 134.07, 133.01, 132.71, 130.58, 128.45, 128.38, 127.20, 124.97, 122.49, 121.72, 121.44, 45.44, 44.67, 44.57, 39.72, 30.00, 29.79, 26.83, 25.88. **IR** (KBr): 3728, 3704, 3627, 3285, 2956, 2920, 2951, 1633, 1609, 1583, 1541, 1326, 757, 669, 532 cm^{-1}.

^{1}H and ^{13}C NMR spectra of compound **2i**.

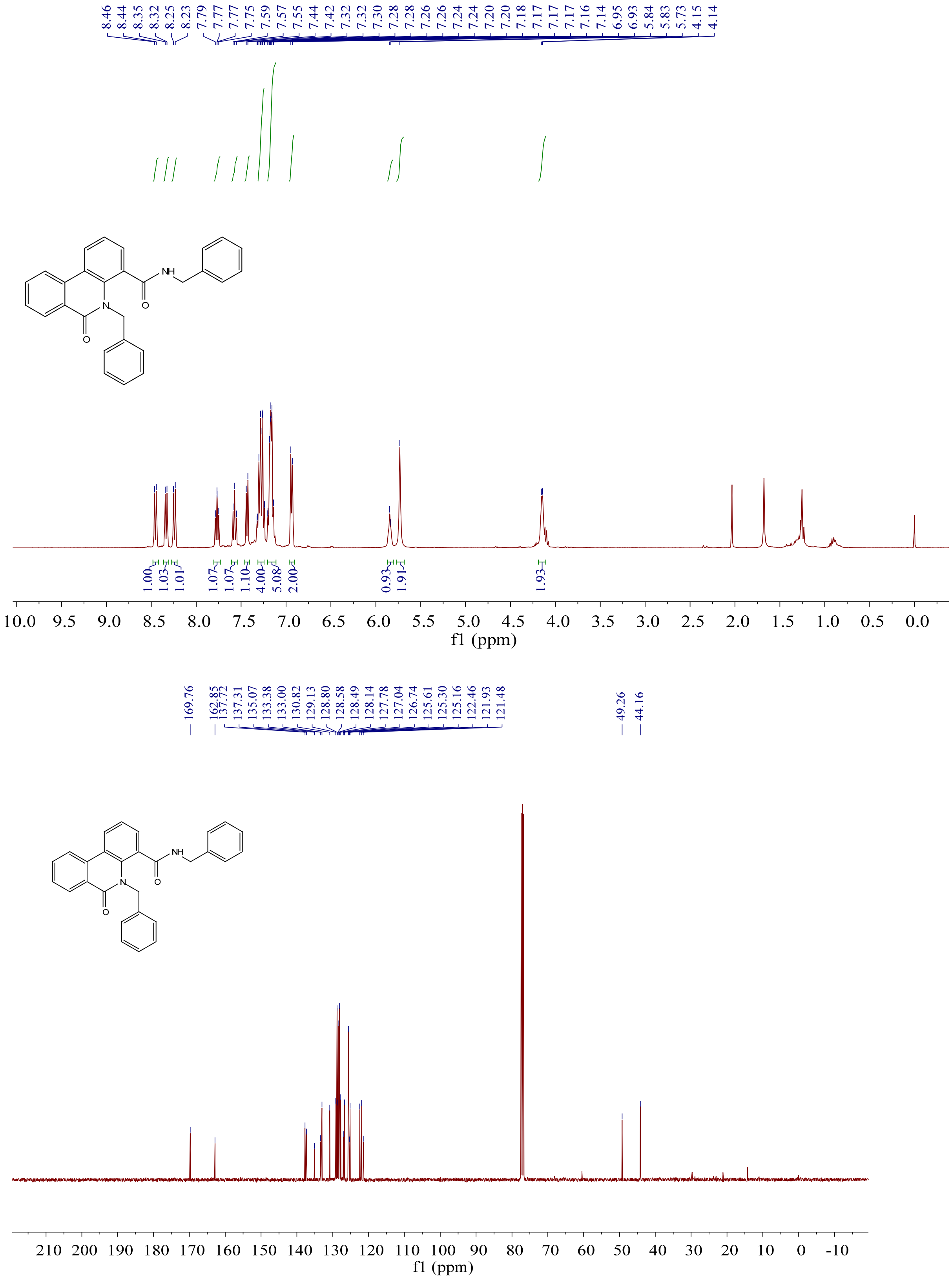

HRMS spectra of compound **2i**.

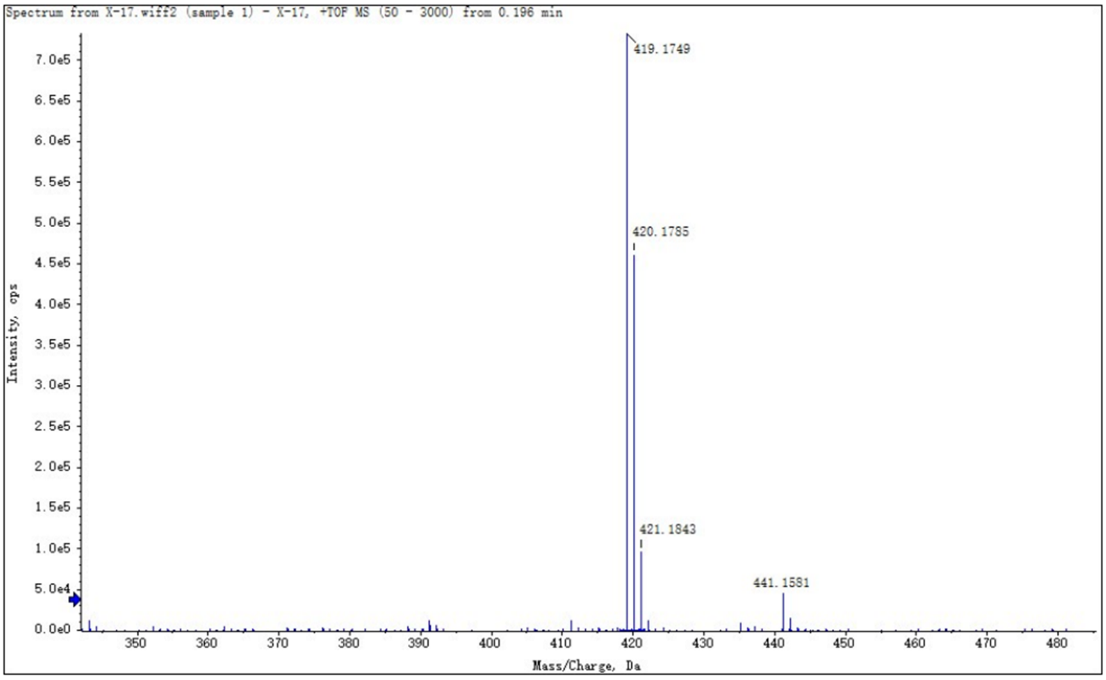

IR spectra of compound **2i**.

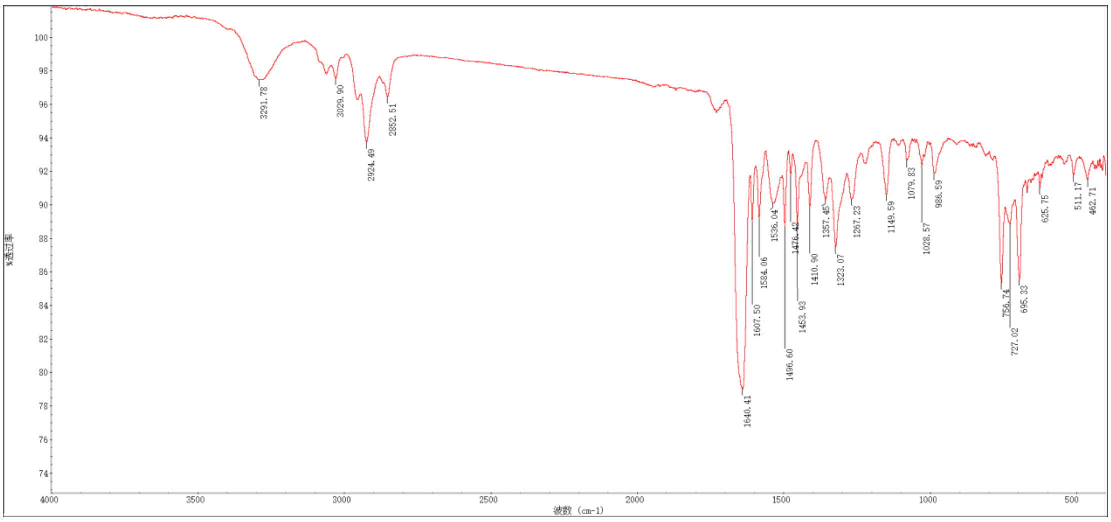

N,5-dibenzyl-6-oxo-5,6-dihydrophenanthridine-4-carboxamide (2i):

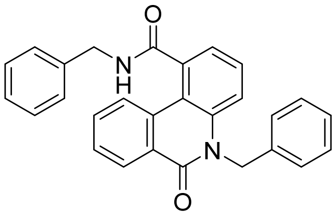

2i

White solid, yield 73%, m.p. 209-211℃; ^{1}**H NMR** (400 MHz, $CDCl_3$) δ 8.45 (d, *J* = 8.0 Hz, 1H), 8.34 (d, *J* = 8.1 Hz, 1H), 8.24 (d, *J* = 8.3 Hz, 1H), 7.77 (dd, *J* = 8.2, 7.2 Hz, 1H), 7.57 (t, *J* = 7.6 Hz, 1H),

7.43 (d, J = 7.4 Hz, 1H), 7.31 – 7.24 (m, 4H), 7.20 – 7.11 (m, 5H), 6.94 (d, J = 7.5 Hz, 2H), 5.84 (d, J = 5.1 Hz, 1H), 5.73 (s, 2H), 4.15 (d, J = 3.5 Hz, 2H). **^{13}C NMR** (101 MHz, $CDCl_3$) δ 169.76, 162.85, 137.72, 137.31, 135.07, 133.38, 133.00, 130.82, 129.13, 128.80, 128.58, 128.49, 128.14, 127.78, 127.04, 126.74, 125.61, 125.30, 125.16, 122.46, 121.93, 121.48, 49.26, 44.16. **HRMS** (ESI-TOF) m/z: $[M+H]^+$ calcd for $C_{28}H_{23}N_2O_2$ 419.1754; found 419.1749. **IR** (KBr): 3292, 3030, 2924, 2853, 1640, 1608, 1584, 1536, 1497, 1476, 1411, 1357, 1323, 1267, 1150, 1080, 1029, 987, 757, 727, 626, 532 cm^{-1}.

^{1}H and ^{13}C NMR spectra of compound **2j**.

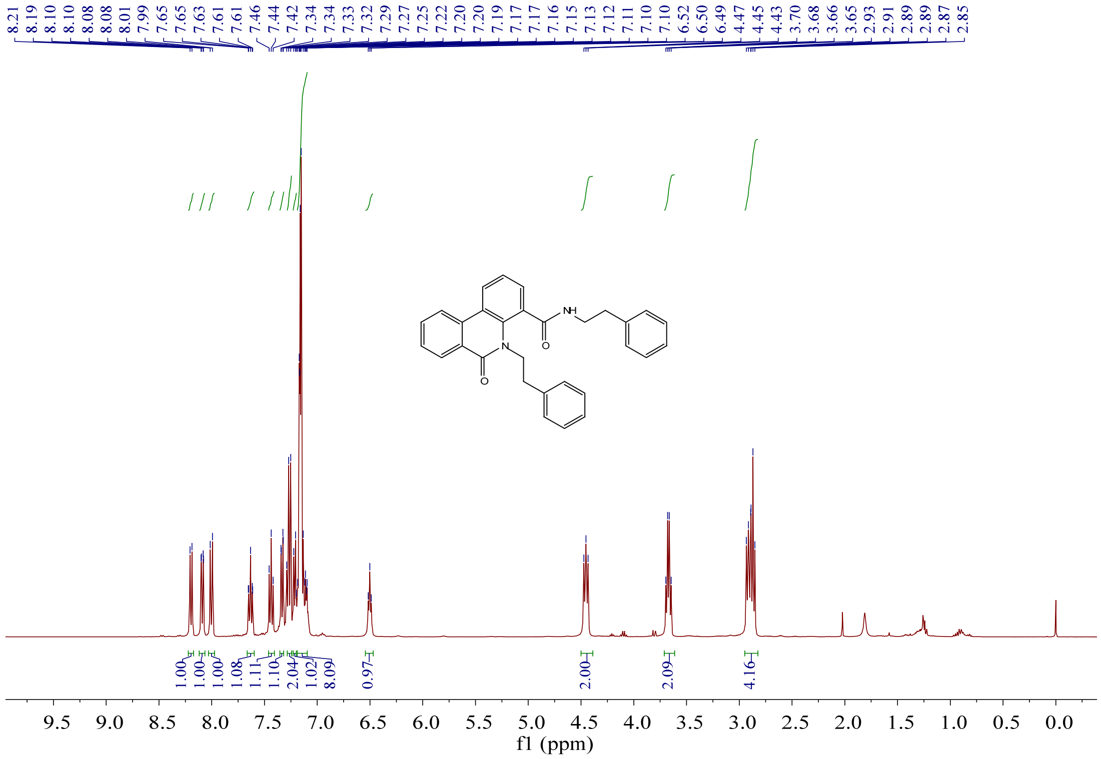

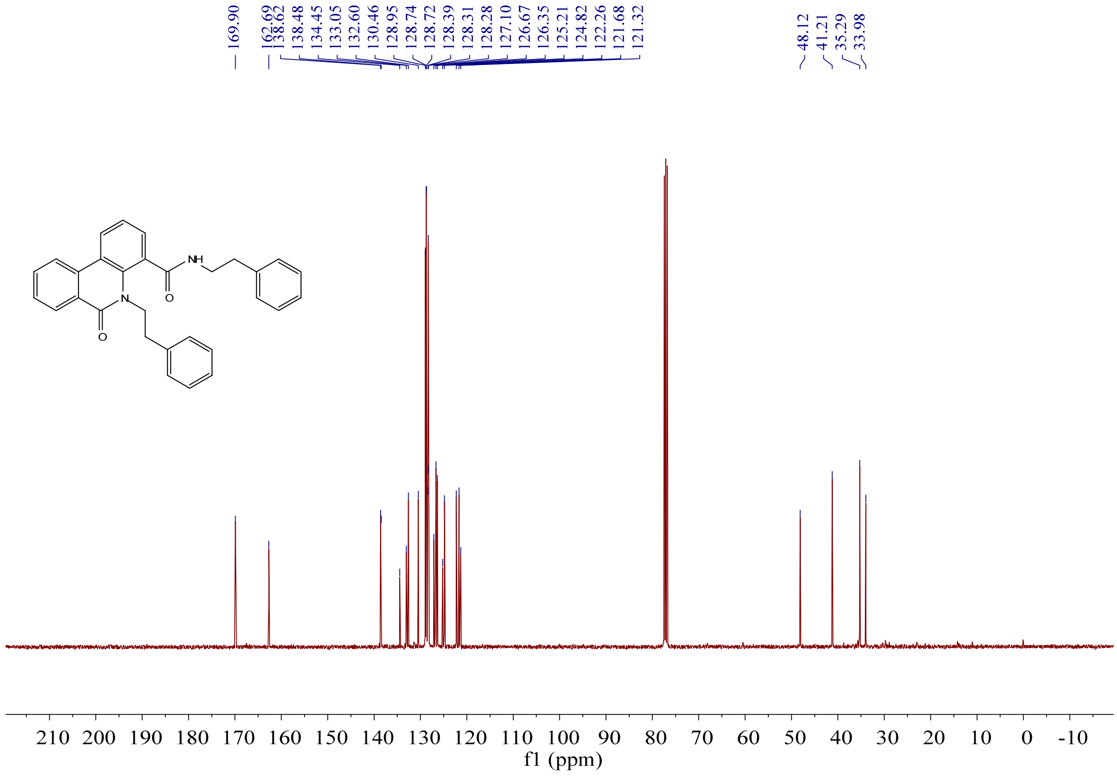

HRMS spectra of compound **2j**.

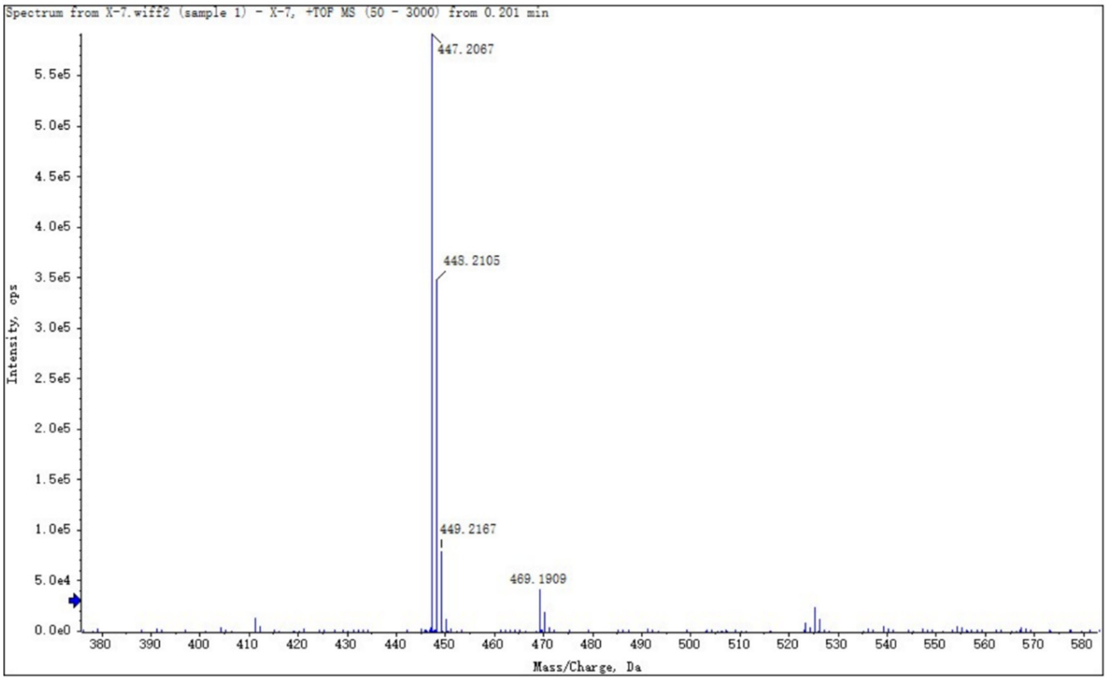

IR spectra of compound **2j**.

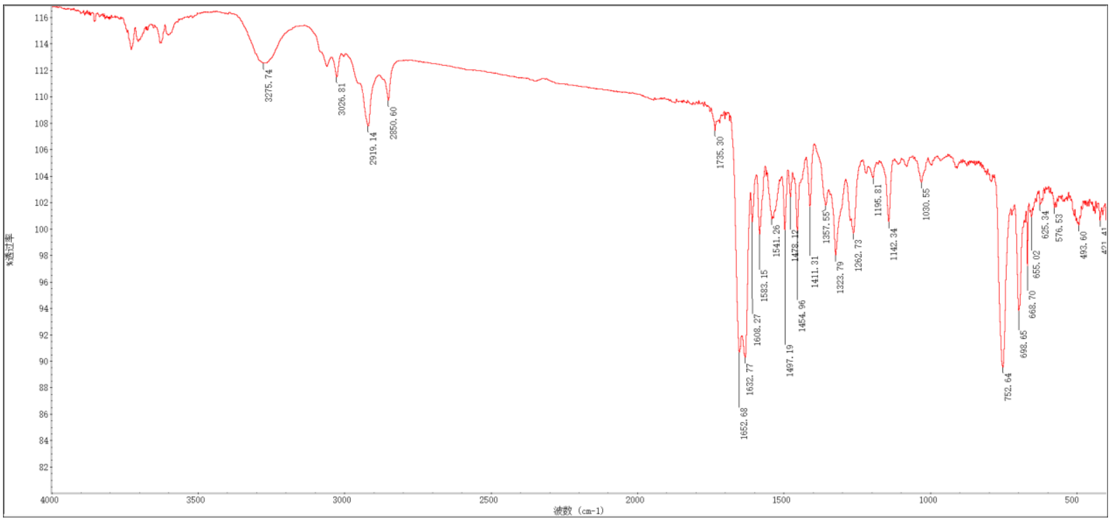

N,5-diphenethyl-6-oxo-5,6-dihydrophenanthridine-4-carboxamide (2j):

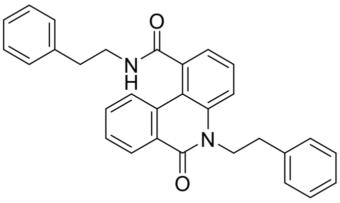

2j

White solid, yield 84%, m.p. 145-147°C; ^{1}**H NMR** (400 MHz, $CDCl_3$) δ 8.20 (d, J = 7.8 Hz, 1H), 8.09 (dd, J = 8.0, 1.1 Hz, 1H), 8.00 (d, J = 8.2 Hz, 1H), 7.66 – 7.60 (m, 1H), 7.44 (t, J = 7.5 Hz, 1H), 7.33 (dd, J = 7.4, 1.2 Hz, 1H), 7.26 (d, J = 7.8 Hz, 2H), 7.21 (d, J = 7.1 Hz, 1H), 7.19 – 7.09 (m, 8H), 6.50 (t,

J = 5.6 Hz, 1H), 4.50 – 4.39 (m, 2H), 3.67 (dd, *J* = 13.1, 7.0 Hz, 2H), 2.89 (dt, *J* = 14.6, 7.5 Hz, 4H). **^{13}C NMR** (101 MHz, $CDCl_3$) δ 169.90, 162.69, 138.62, 138.48, 134.45, 133.05, 132.60, 130.46, 128.95, 128.74, 128.72, 128.39, 128.31, 128.28, 127.10, 126.67, 126.35, 125.21, 124.82, 122.26, 121.68, 121.32, 48.12, 41.21, 35.29, 33.98. **HRMS** (ESI-TOF) m/z: $[M+H]^+$ calcd for $C_{30}H_{27}N_2O_2$ 447.2067; found 447.2067. **IR** (KBr): 3276, 3027, 2919, 2950, 1735, 1653, 1608, 1583, 1541, 1497, 1455, 1411, 1358, 1324, 1263, 1142, 1031, 753, 699, 625, 532 cm^{-1}.

^{1}H and ^{13}C NMR spectra of compound **2k**.

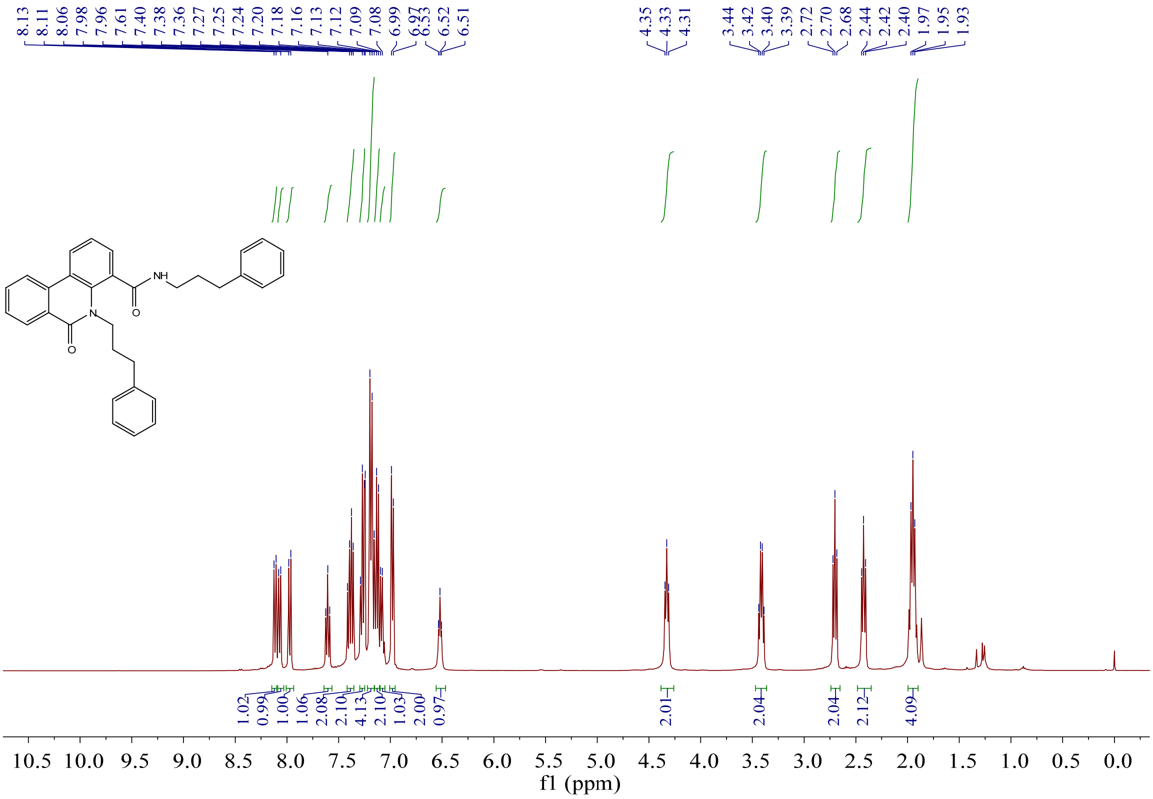

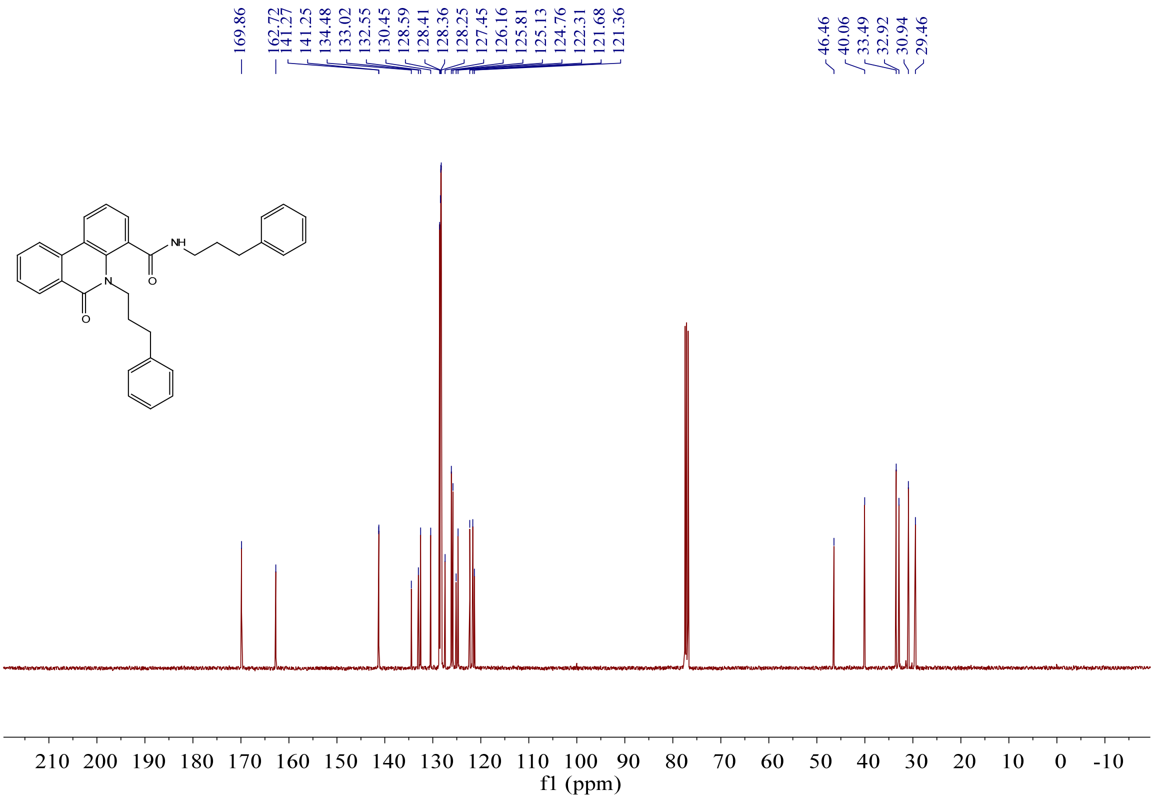

HRMS spectra of compound **2k**.

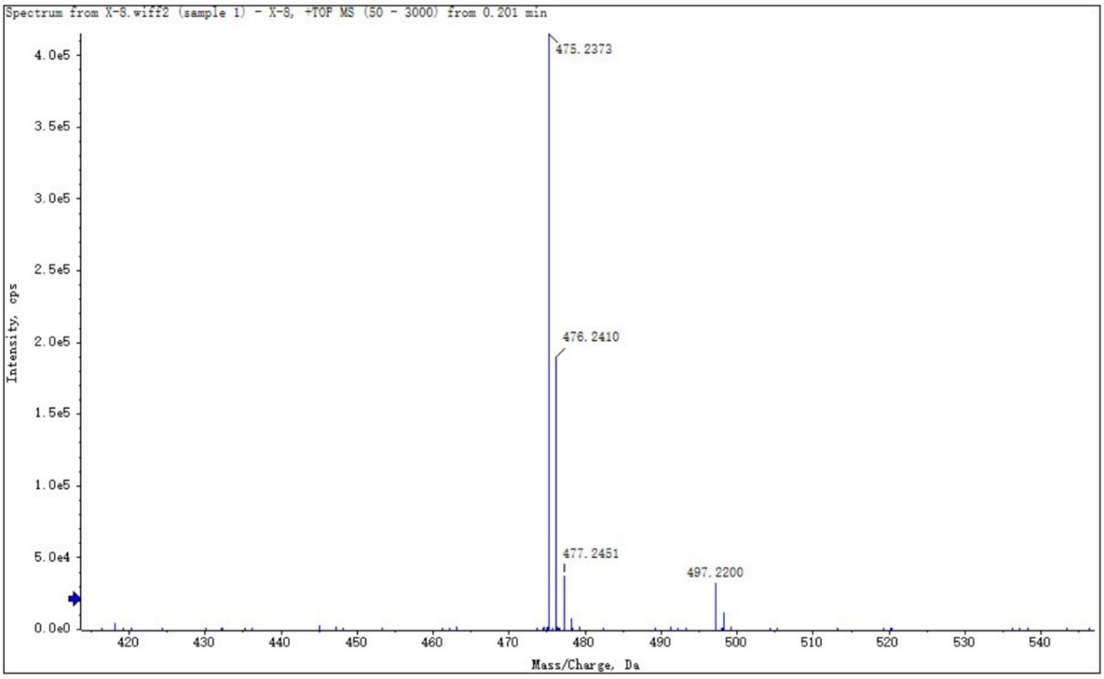

IR spectra of compound **2k**.

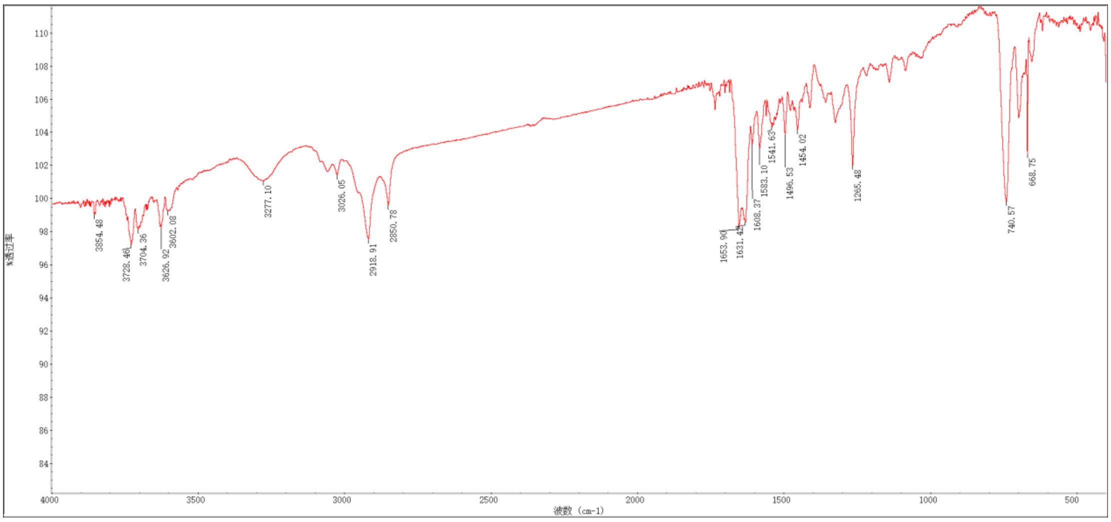

N,5-bis(3-phenylpropyl)-6-oxo-5,6-dihydrophenanthridine-4-carboxamide (2k):

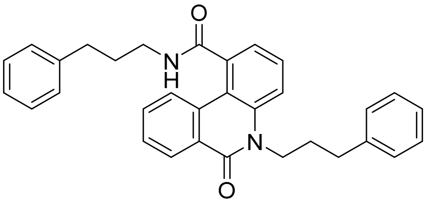

2k

White solid, yield 89%; **^{1}H NMR** (400 MHz, $CDCl_3$) δ 8.12 (d, *J* = 7.9 Hz, 1H), 8.07 (d, *J* = 7.8 Hz, 1H), 7.97 (d, *J* = 8.2 Hz, 1H), 7.61 (t, *J* = 7.6 Hz, 1H), 7.39 (dd, *J* = 14.9, 7.4 Hz, 2H), 7.30 – 7.25 (m, 2H), 7.22 – 7.16 (m, 4H), 7.12 (d, *J* = 7.5 Hz, 2H), 7.09 (d, *J* = 6.9 Hz, 1H), 6.98 (d, *J* = 7.2 Hz, 2H),

6.52 (t, *J* = 5.5 Hz, 1H), 4.33 (t, *J* = 7.3 Hz, 2H), 3.41 (dd, *J* = 13.5, 6.7 Hz, 2H), 2.70 (t, *J* = 7.6 Hz, 2H), 2.48 – 2.35 (m, 2H), 2.00 – 1.90 (m, 4H). **^{13}C NMR** (101 MHz, $CDCl_3$) δ 169.86, 162.72, 141.27, 141.25, 134.48, 133.02, 132.55, 130.45, 128.59, 128.41, 128.36, 128.25, 127.45, 126.16, 125.81, 125.13, 124.76, 122.31, 121.68, 121.36, 46.46, 40.06, 33.49, 32.92, 30.94, 29.46. **HRMS** (ESI-TOF) m/z: $[M+H]^+$ calcd for $C_{32}H_{31}N_2O_2$ 475.2380; found475.2373. **IR** (KBr): 3728, 3627, 3602, 3277, 3026, 2919, 2851, 1654, 1608, 1583, 1542, 1497, 1454, 1265, 741, 669, 532 cm^{-1}.

^{1}H and ^{13}C NMR spectra of compound **2l**.

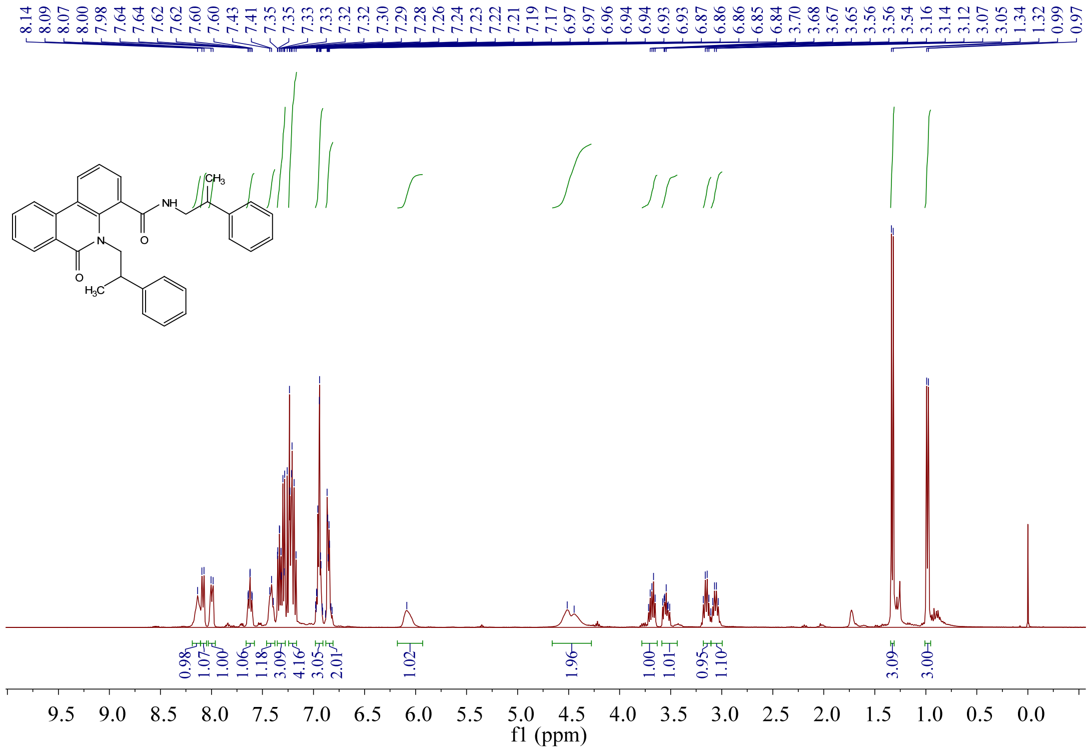

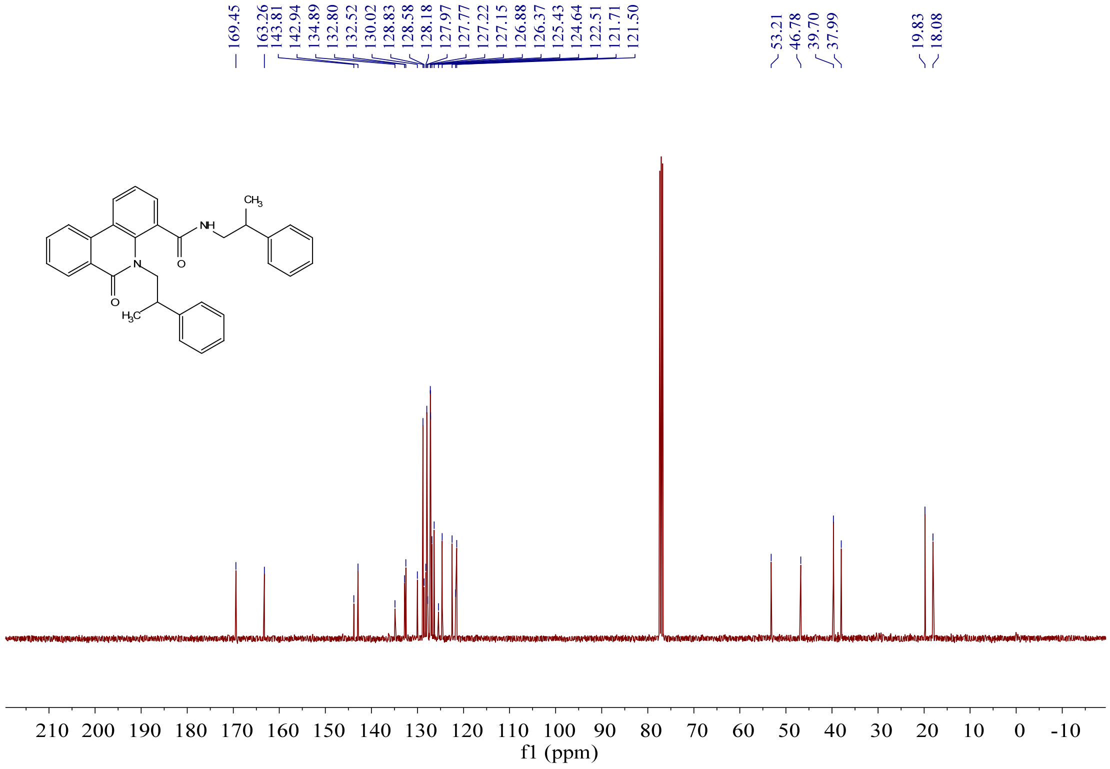

HRMS spectra of compound **2l**.

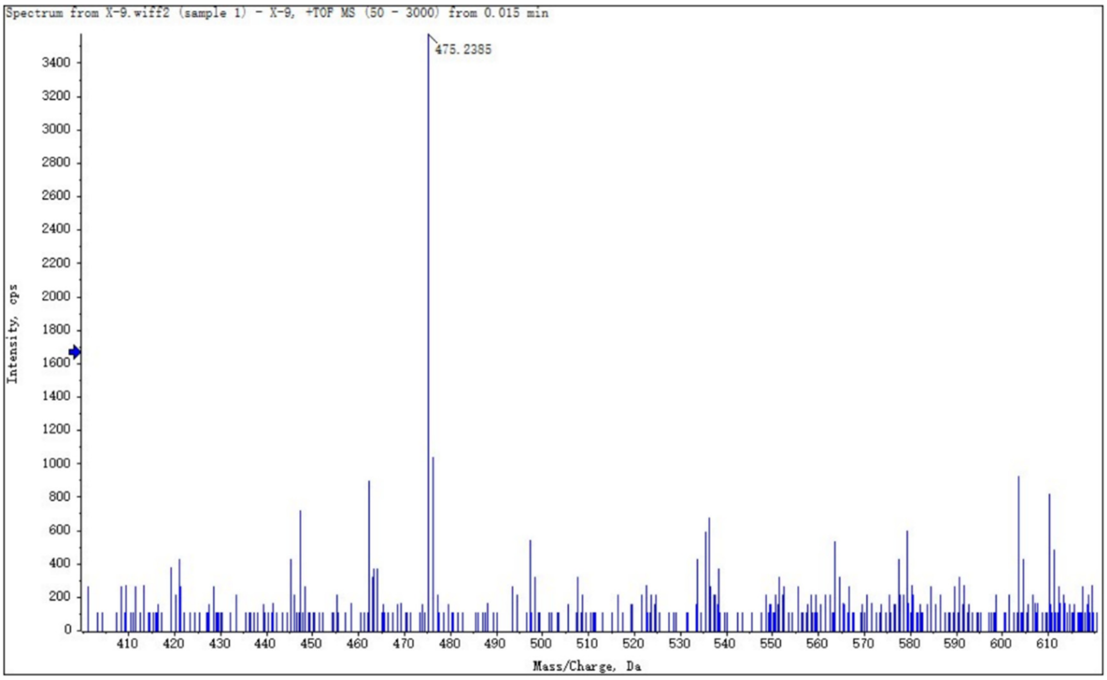

IR spectra of compound **2l**.

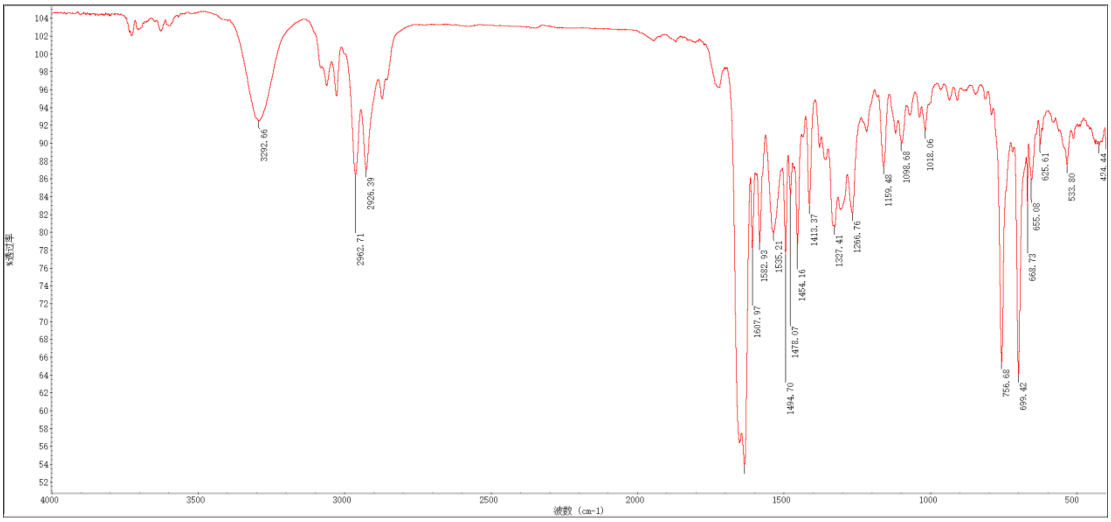

N,5-bis(2-phenylpropyl)-6-oxo-5,6-dihydrophenanthridine-4-carboxamide (2l):

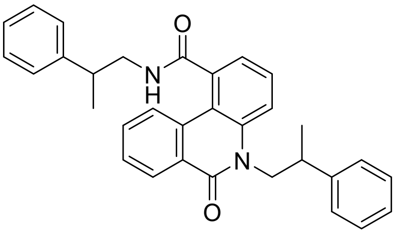

2l

White solid, yield 80%, m.p. 120-122℃; **^{1}H NMR** (400 MHz, $CDCl_3$) δ 8.14 (s, 1H), 8.08 (d, *J* = 8.1 Hz, 1H), 7.99 (d, *J* = 7.8 Hz, 1H), 7.66 – 7.58 (m, 1H), 7.41 (t, *J* = 7.0 Hz, 1H), 7.36 – 7.28 (m, 3H),

7.24 – 7.17 (m, 4H), 6.98 – 6.91 (m, 3H), 6.88 – 6.81 (m, 2H), 6.08 (s, 1H), 4.48 (d, *J* = 26.8 Hz, 2H), 3.68 (dt, *J* = 12.8, 6.2 Hz, 1H), 3.59 – 3.44 (m, 1H), 3.14 (dt, *J* = 14.8, 7.4 Hz, 1H), 3.06 (dd, *J* = 14.6, 7.2 Hz, 1H), 1.33 (d, *J* = 7.0 Hz, 3H), 0.98 (d, *J* = 6.9 Hz, 3H). **^{13}C NMR** (101 MHz, $CDCl_3$) δ 169.45, 163.26, 143.81, 142.94, 134.89, 132.80, 132.52, 130.02, 128.83, 128.58, 128.18, 127.97, 127.77, 127.22, 127.15, 126.88, 126.37, 125.43, 124.64, 122.51, 121.71, 121.50, 53.21, 46.78, 39.70, 37.99, 19.83, 18.08. **HRMS** (ESI-TOF) m/z: $[M+H]^+$ calcd for $C_{32}H_{31}N_2O_2$ 475.2380; found 475.3285. **IR** (KBr): 3293, 2963, 2926, 1608, 1583, 1535, 1495, 1478, 1413, 1327, 1267, 1159, 1099, 1018, 757, 699, 626, 532 cm^{-1}.

^{1}H and ^{13}C NMR spectra of compound **2m**.

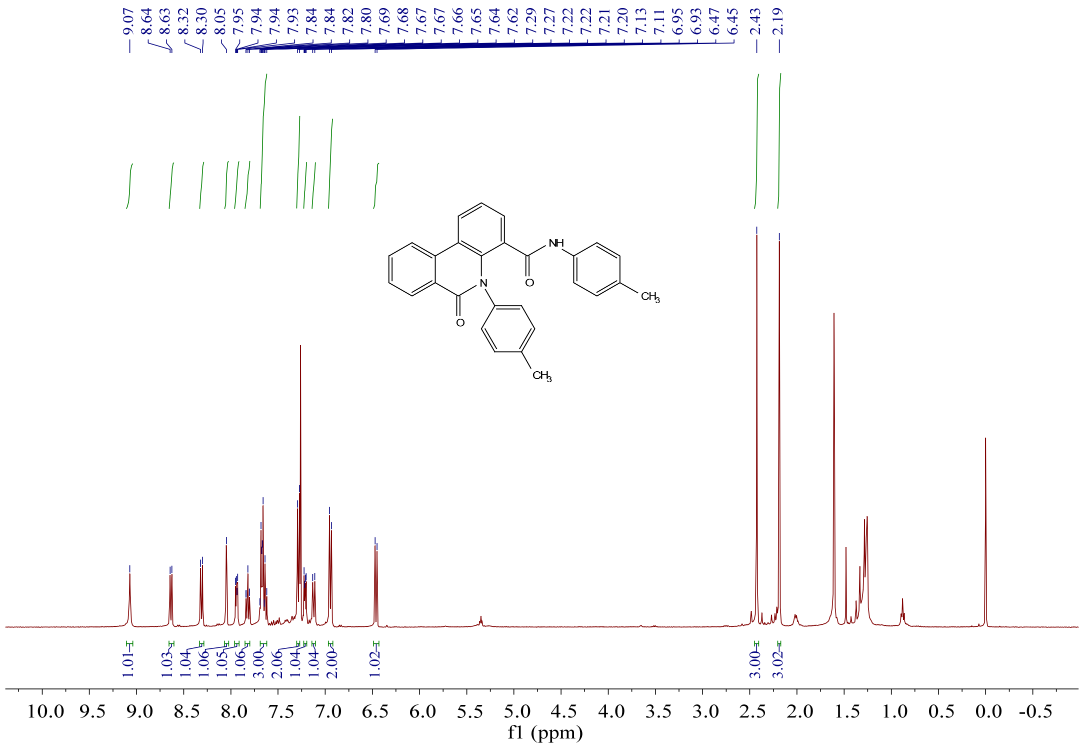

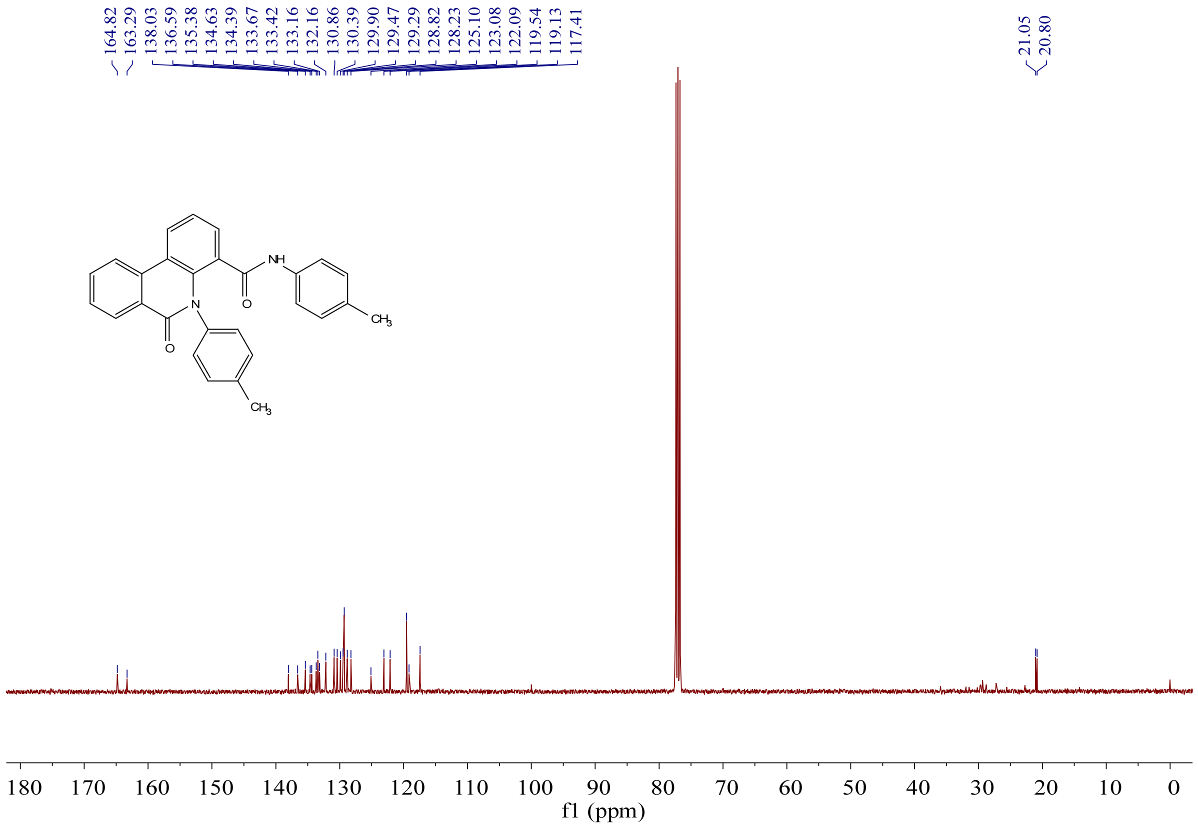

HRMS spectra of compound **2m**.

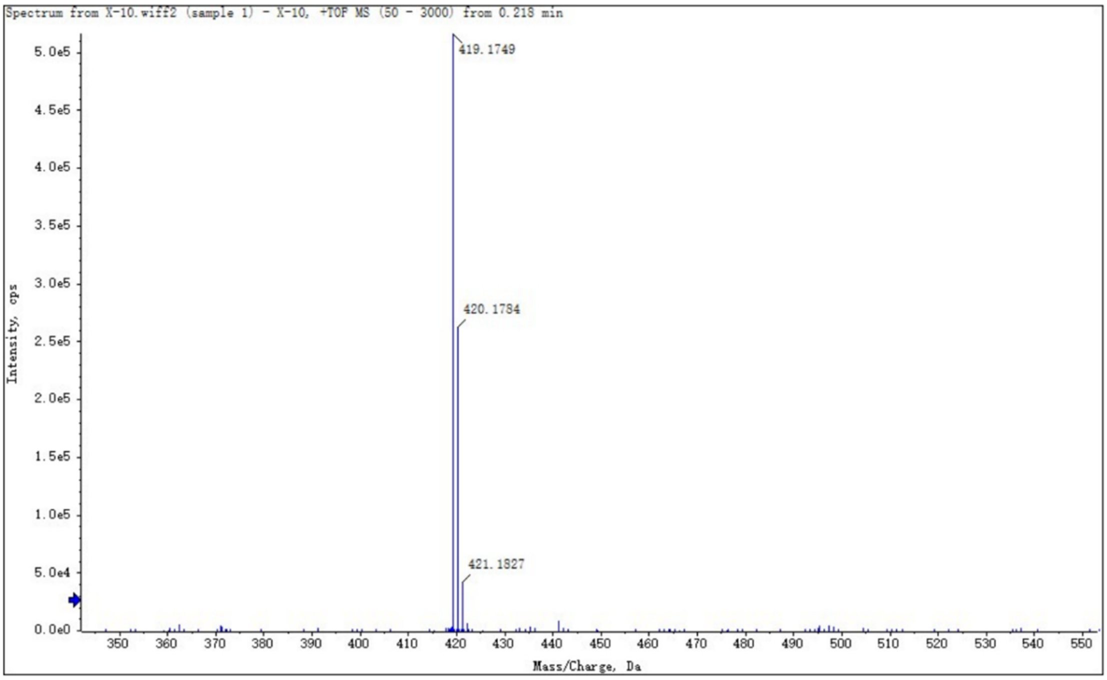

IR spectra of compound **2m**.

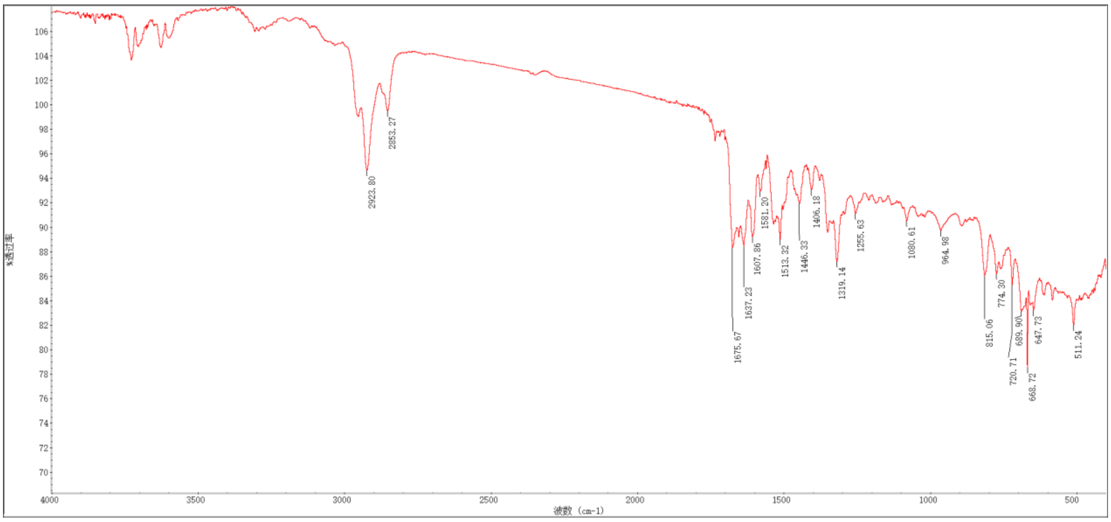

N,5-di-p-tolyl-6-oxo-5,6-dihydrophenanthridine-4-carboxamide (2m):

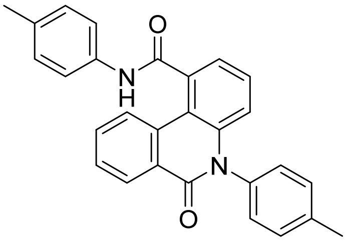

2m

White solid, yield 51%; **^{1}H NMR** (400 MHz, $CDCl_3$) δ 9.07 (s, 1H), 8.63 (d, *J* = 7.5 Hz, 1H), 8.31 (d, *J* = 8.2 Hz, 1H), 8.05 (s, 1H), 7.94 (dd, *J* = 6.0, 3.3 Hz, 1H), 7.83 (dd, *J* = 11.3, 4.1 Hz, 1H), 7.69 – 7.62

(m, 3H), 7.28 (d, *J* = 8.4 Hz, 2H), 7.21 (dd, *J* = 5.9, 3.1 Hz, 1H), 7.12 (d, *J* = 8.6 Hz, 1H), 6.94 (d, *J* = 8.3 Hz, 2H), 6.46 (d, *J* = 8.5 Hz, 1H), 2.43 (s, 3H), 2.19 (s, 3H). **^{13}C NMR** (101 MHz, $CDCl_3$) δ 164.82, 163.29, 138.03, 136.59, 135.38, 134.63, 134.39, 133.67, 133.42, 133.16, 132.16, 130.86, 130.39, 129.90, 129.47, 129.29, 128.82, 128.23, 125.10, 123.08, 122.09, 119.54, 119.13, 117.41, 21.05, 20.80. **HRMS** (ESI-TOF) m/z: $[M+H]^+$ calcd for $C_{28}H_{23}N_2O_2$ 419.1754; found 419.1749. **IR** (KBr): 2924, 2853, 1676, 1608, 1513, 1446, 1319, 1081, 815, 669, 532 cm^{-1}.

^{1}H, ^{13}C, and ^{19}F NMR spectra of compound **2n**.

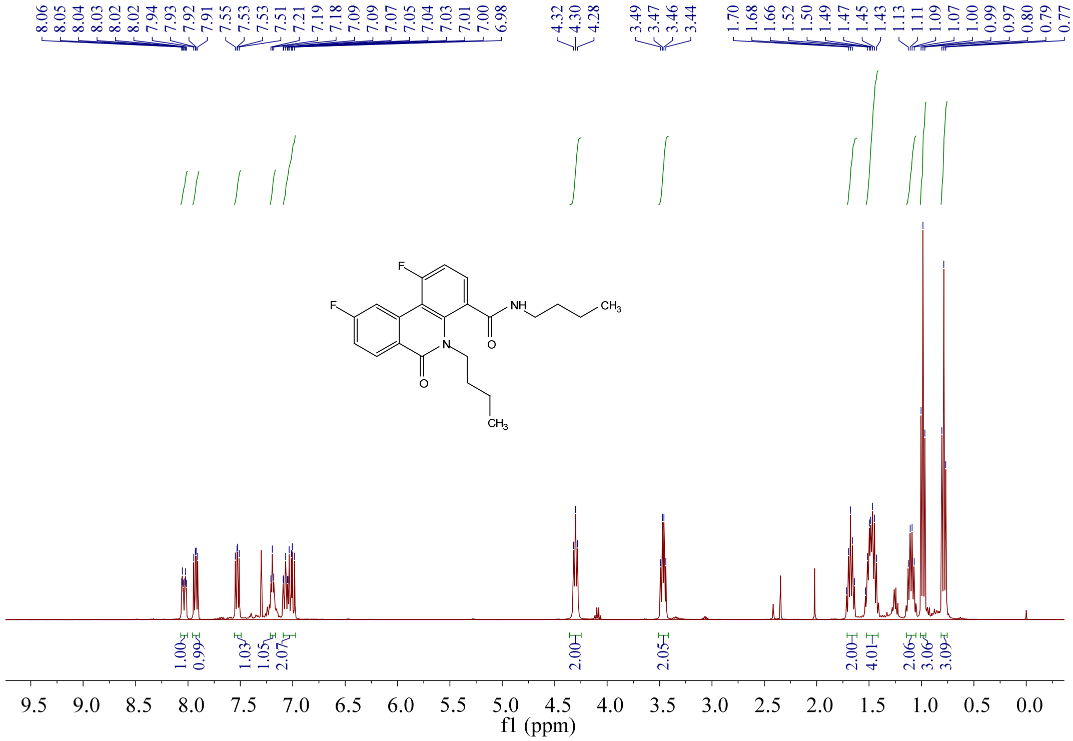

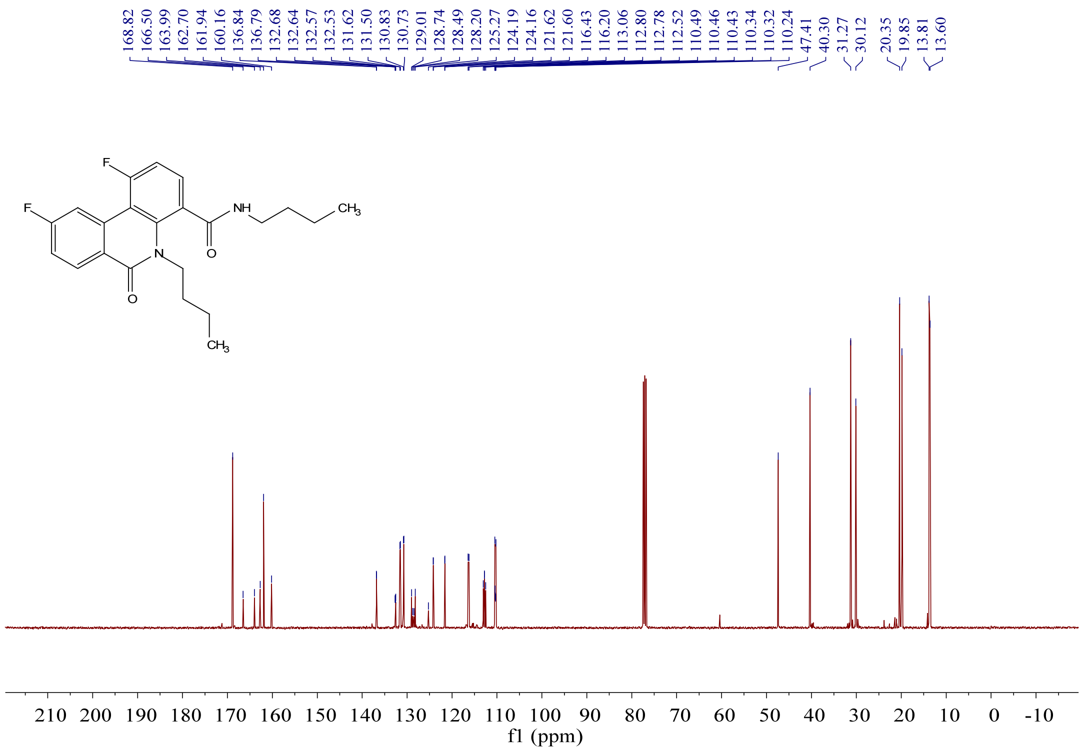

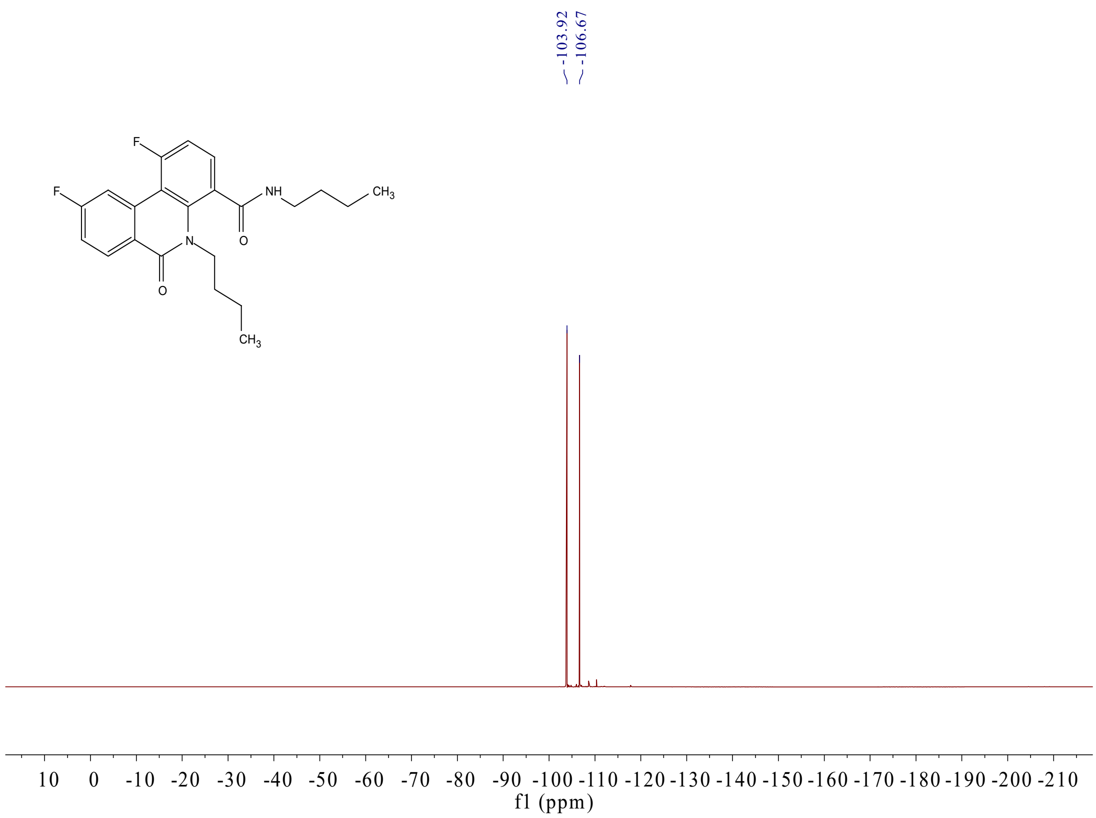

HRMS spectra of compound **2n**.

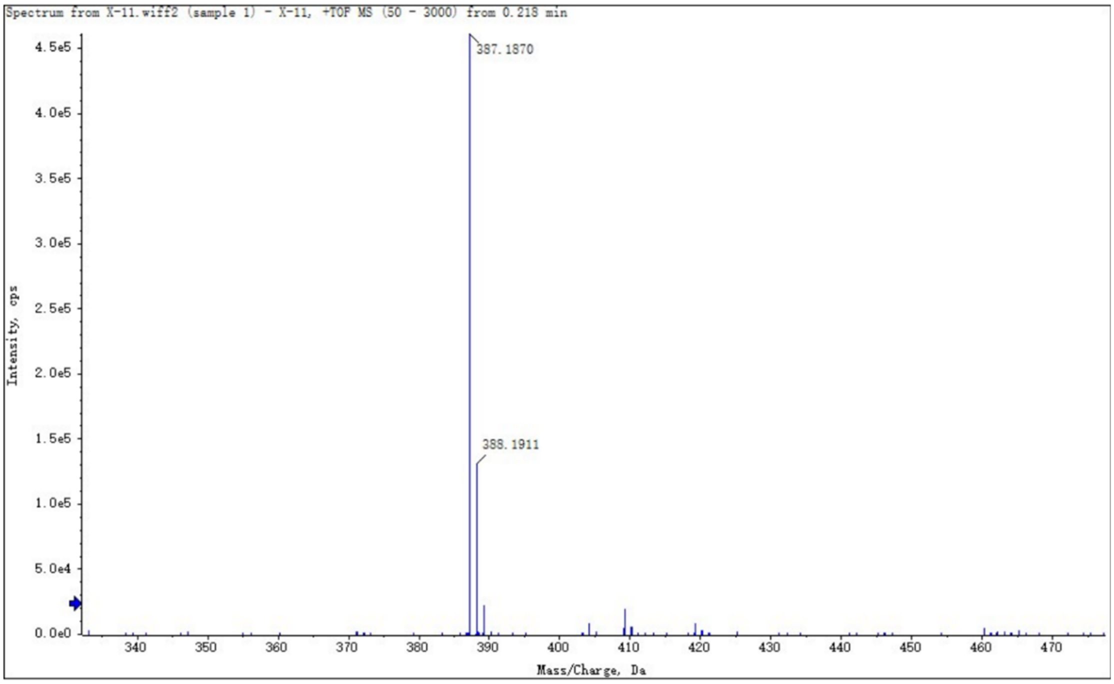

IR spectra of compound **2n**.

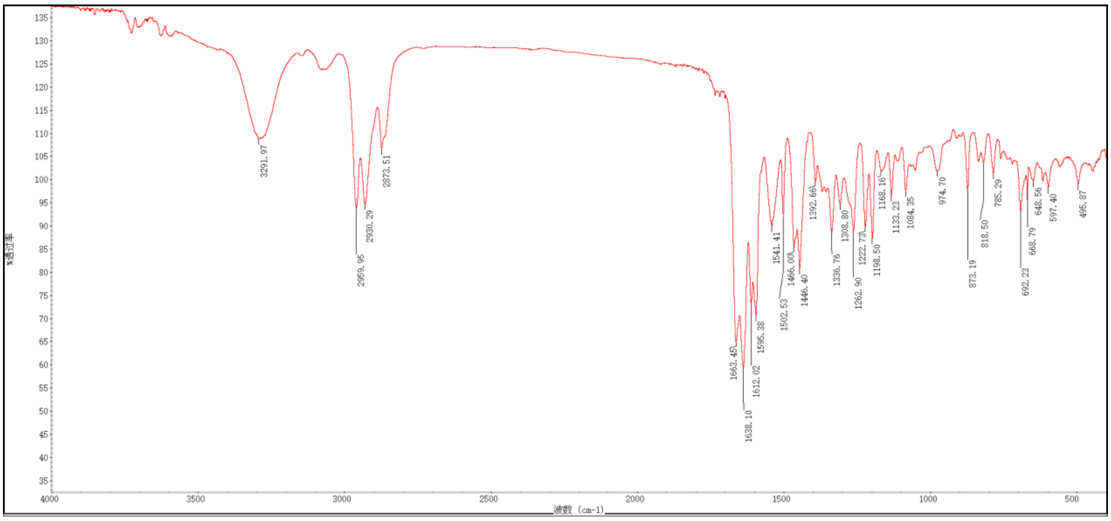

N,5-dibutyl-1,9-difluoro-6-oxo-5,6-dihydrophenanthridine-4-carboxamide (2n):

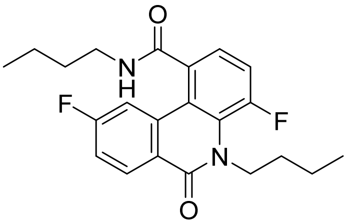

2n

White solid, yield 78%, m.p. 165-167°C; ^{1}**H NMR** (400 MHz, $CDCl_3$) δ 8.07 – 8.00 (m, 1H), 7.93 (dd, *J* = 8.8, 6.3 Hz, 1H), 7.53 (dd, *J* = 8.4, 6.1 Hz, 1H), 7.19 (t, *J* = 5.5 Hz, 1H), 7.04 (ddd, *J* = 25.0, 13.3, 7.7 Hz, 2H), 4.30 (t, *J* = 7.4 Hz, 2H), 3.46 (dd, *J* = 13.1, 7.1 Hz, 2H), 1.67 (dd, *J* = 14.9, 7.7 Hz, 2H), 1.47 (dt, *J* = 22.4, 7.5 Hz, 4H), 1.10 (dd, *J* = 15.1, 7.5 Hz, 2H), 0.99 (t, *J* = 7.4 Hz, 3H), 0.79 (t, *J* = 7.4 Hz, 3H). ^{13}**C NMR** (101 MHz, $CDCl_3$) δ 168.82, 166.50, 163.99, 162.70, 161.94, 160.16, 136.84, 136.79, 132.68, 132.64, 132.57, 132.53, 131.62, 131.50, 130.83, 130.73, 129.01, 128.74, 128.49, 128.20, 125.27, 124.19, 124.16, 121.62, 121.60, 116.43, 116.20, 113.06, 112.80, 112.78, 112.52, 110.49, 110.46, 110.43, 110.34, 110.32, 110.24, 47.41, 40.30, 31.27, 30.12, 20.35, 19.85, 13.81, 13.60. ^{19}**F NMR** (376 MHz, $CDCl_3$) δ -103.92, -106.67. **HRMS** (ESI-TOF) m/z: $[M+H]^+$ calcd for $C_{22}H_{25}F_2N_2O_2$ 387.1879; found 387.1870. **IR** (KBr): 3292, 2960, 2930, 2874, 1663, 1638, 1612, 1595, 1541, 1503, 1466, 1446, 1337, 1309, 1263, 1223, 1168, 1084, 975, 873, 785, 692, 649, 532 cm^{-1}.

^{1}H and ^{13}C NMR spectra of compound **2o**.

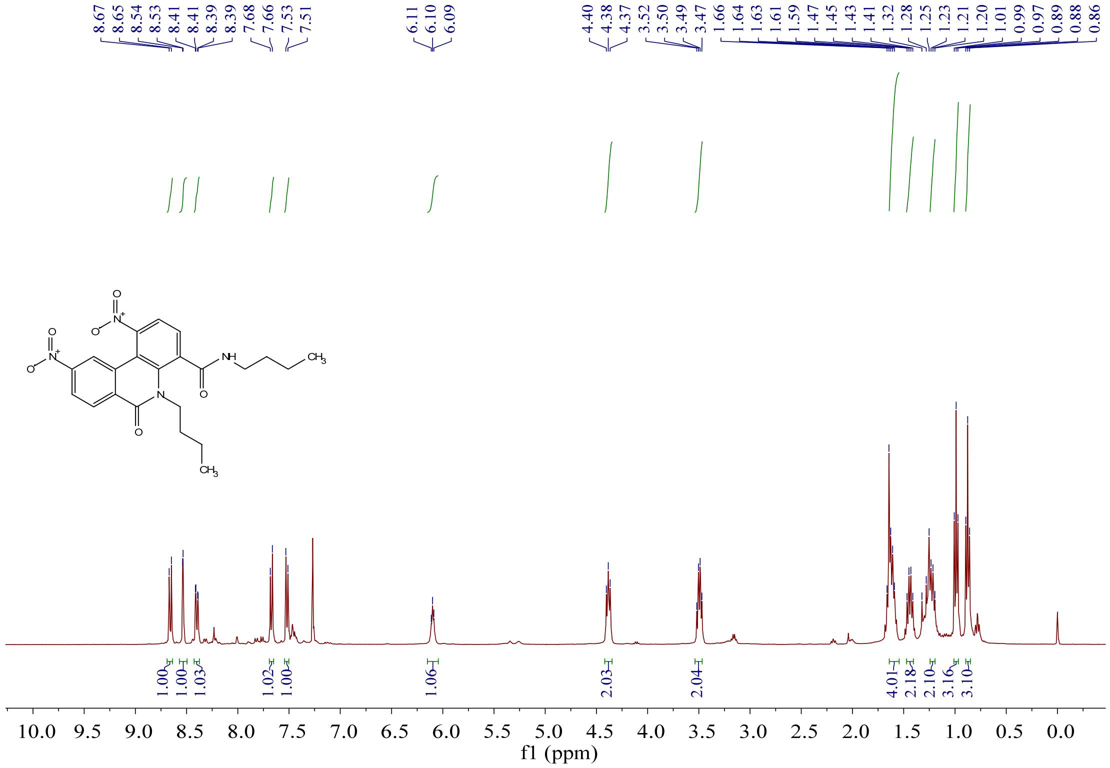

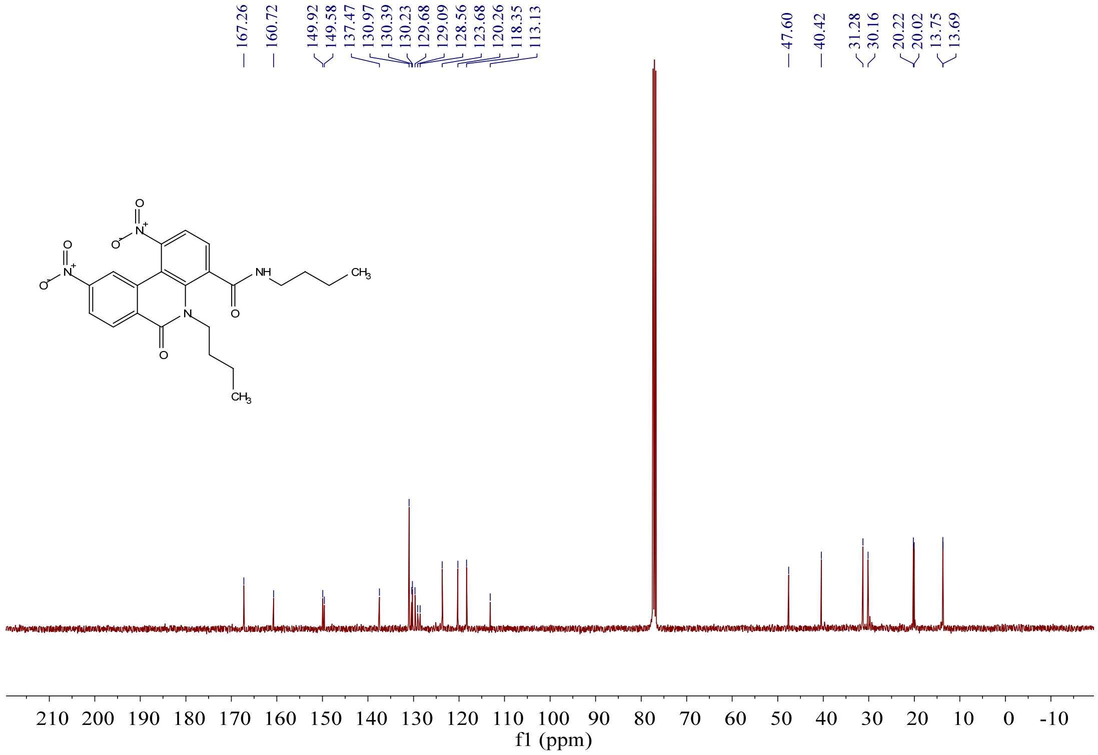

HRMS spectra of compound **2o**.

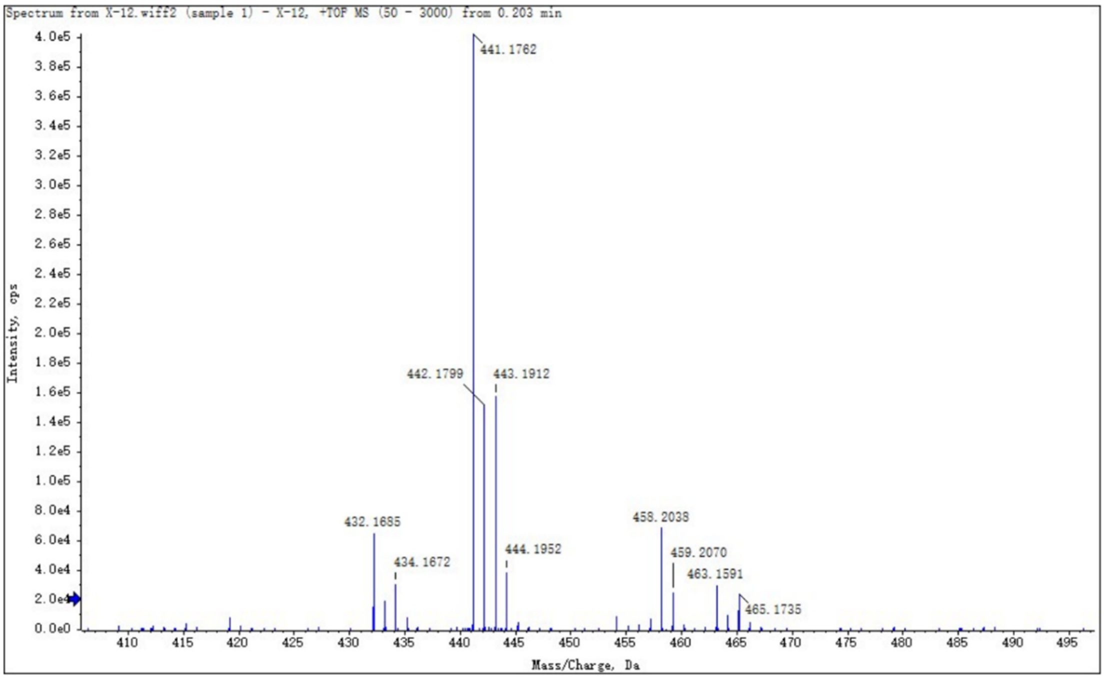

IR spectra of compound **2o**.

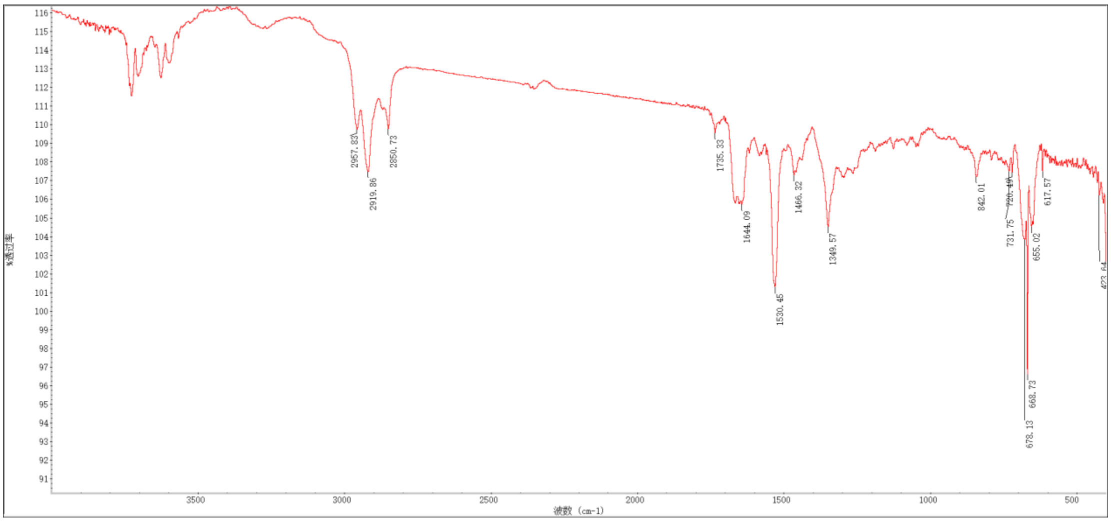

N,5-dibutyl-1,9-dinitro-6-oxo-5,6-dihydrophenanthridine-4-carboxamide (2o):

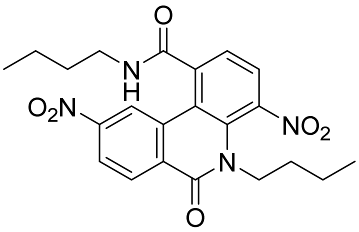

2o

White solid, yield 65%, m.p. 182-184°C; **^{1}H NMR** (400 MHz, $CDCl_3$) δ 8.66 (d, *J* = 8.7 Hz, 1H), 8.54 (d, *J* = 1.6 Hz, 1H), 8.40 (dd, *J* = 8.7, 1.8 Hz, 1H), 7.67 (d, *J* = 8.0 Hz, 1H), 7.52 (d, *J* = 8.0 Hz, 1H),

6.10 (t, *J* = 5.2 Hz, 1H), 4.42 – 4.35 (m, 2H), 3.50 (dd, *J* = 13.2, 7.0 Hz, 2H), 1.64 – 1.54 (m, 4H), 1.44 (dd, *J* = 15.0, 7.4 Hz, 2H), 1.24 – 1.19 (m, 2H), 0.99 (t, *J* = 7.3 Hz, 3H), 0.88 (t, *J* = 7.3 Hz, 3H). **^{13}C NMR** (101 MHz, $CDCl_3$) δ 167.26, 160.72, 149.92, 149.58, 137.47, 130.97, 130.39, 130.23, 129.68, 129.09, 128.56, 123.68, 120.26, 118.35, 113.13, 47.60, 40.42, 31.28, 30.16, 20.22, 20.02, 13.75, 13.69. **HRMS** (ESI-TOF) m/z: $[M + H]^+$ calcd for $C_{22}H_{25}N_4O_6$ 441.1769; found 441.1762. **IR** (KBr): 2958, 2920, 2851, 1735, 1644, 1530, 1466, 1350, 842, 732, 678, 618, 532 cm^{-1}.

^{1}H and ^{13}C NMR spectra of compound **2p**.

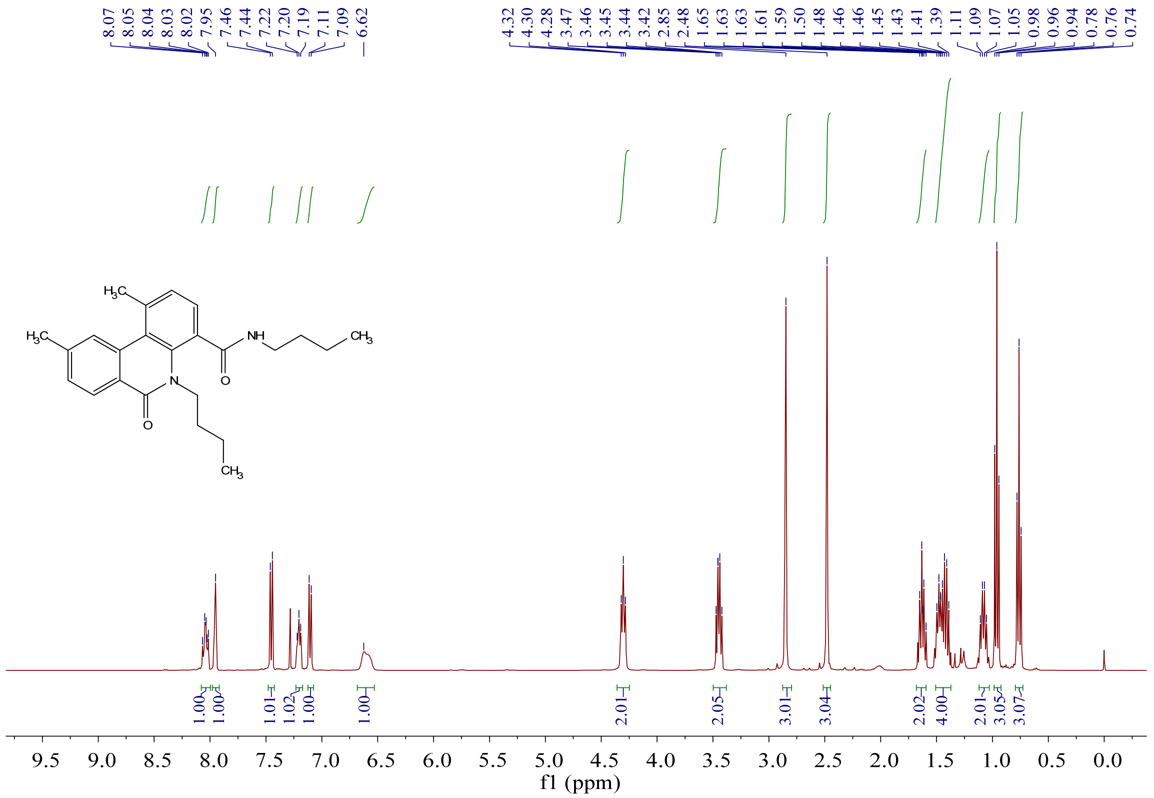

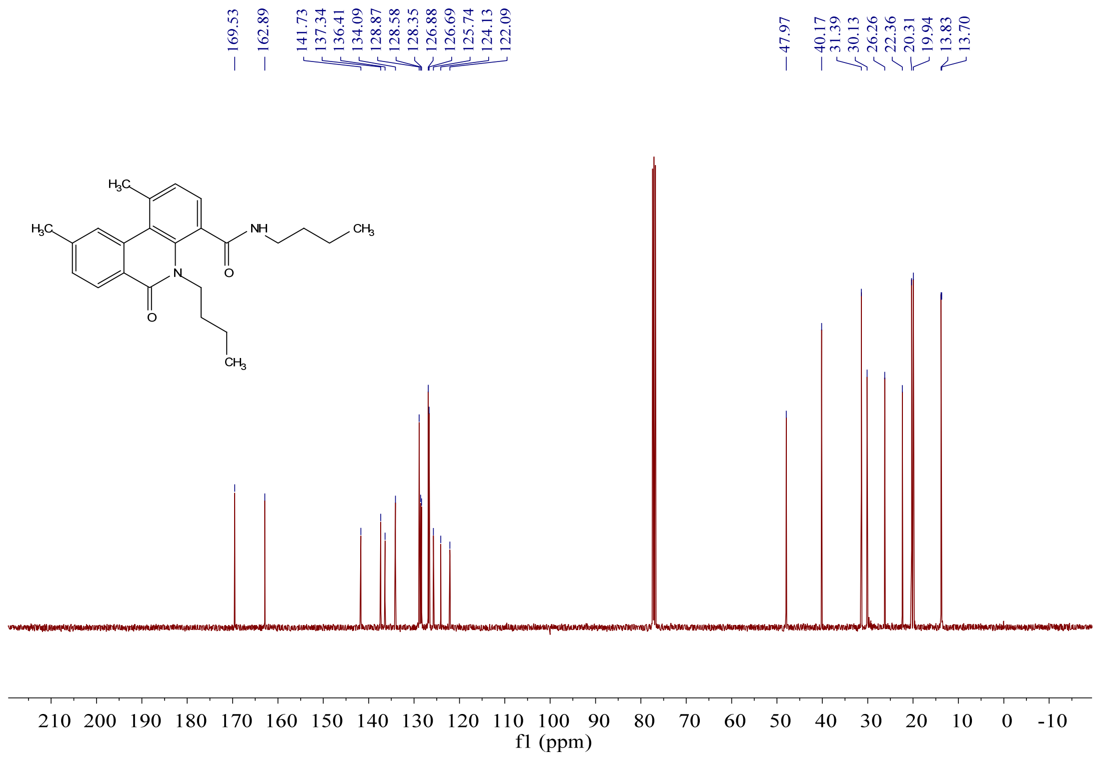

HRMS spectra of compound **2p**.

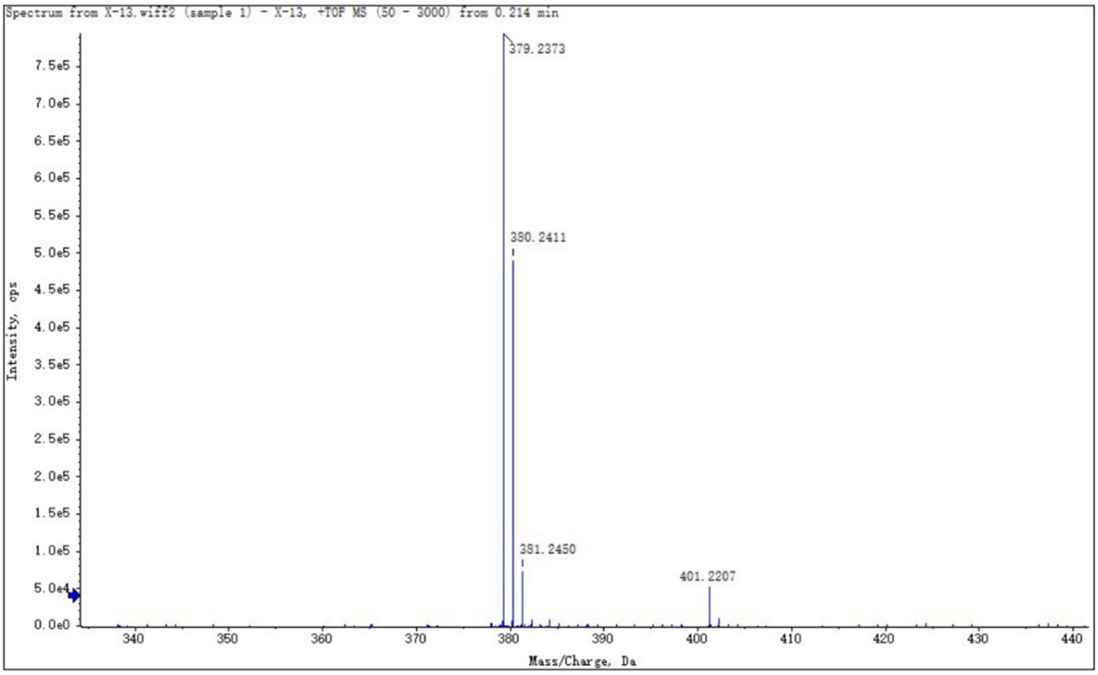

IR spectra of compound **2p**.

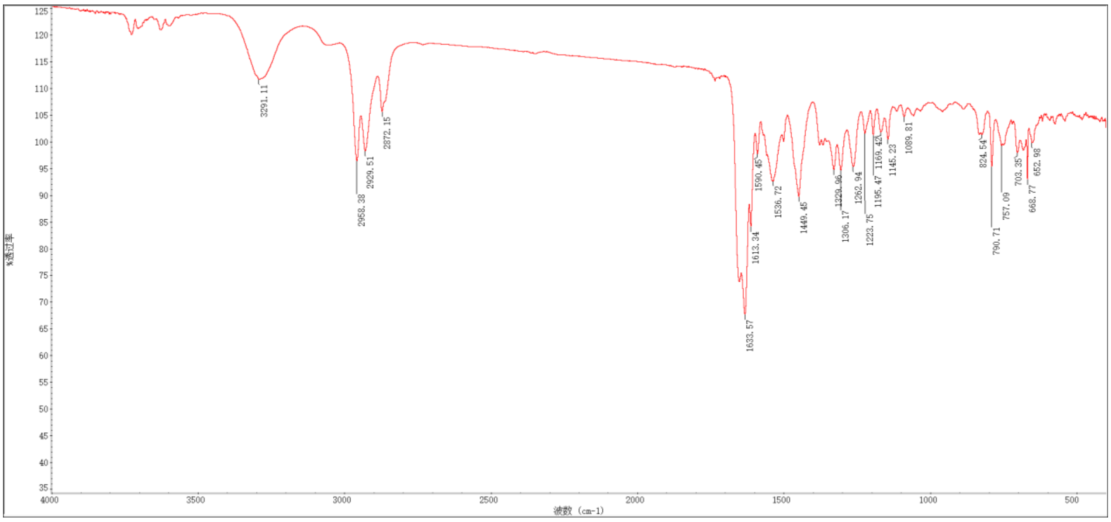

N,5-dibutyl-1,9-dimethyl-6-oxo-5,6-dihydrophenanthridine-4-carboxamide (2p):

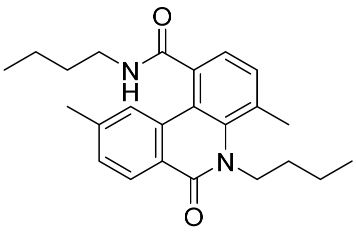

2p

White solid, yield 84%, m.p. 154-156°C; **^{1}H NMR** (400 MHz, $CDCl_3$) δ 8.08 – 8.00 (m, 1H), 7.95 (s, 1H), 7.45 (d, *J* = 7.7 Hz, 1H), 7.20 (t, *J* = 6.4 Hz, 1H), 7.10 (d, *J* = 7.8 Hz, 1H), 6.62 (s, 1H), 4.30 (t, *J*

= 7.3 Hz, 2H), 3.45 (dt, J = 13.4, 6.7 Hz, 2H), 2.85 (s, 3H), 2.48 (s, 3H), 1.68 – 1.59 (m, 2H), 1.45 (ddd, J = 22.4, 14.2, 7.5 Hz, 4H), 1.08 (dd, J = 15.1, 7.5 Hz, 2H), 0.96 (t, J = 7.3 Hz, 3H), 0.76 (t, J = 7.4 Hz, 3H). **^{13}C NMR** (101 MHz, $CDCl_3$) δ 169.53, 162.89, 141.73, 137.34, 136.41, 134.09, 128.87, 128.58, 128.35, 126.88, 126.69, 125.74, 124.13, 122.09, 47.97, 40.17, 31.39, 30.13, 26.26, 22.36, 20.31, 19.94, 13.83, 13.70. **HRMS** (ESI-TOF) m/z: $[M+H]^+$ calcd for $C_{24}H_{31}N_2O_2$ 379.2380; found 379.2373. **IR** (KBr): 3291, 2958, 2930, 2872, 1634, 1613, 1590, 1537, 1449, 1330, 1306, 1263, 1224, 1145, 1090, 791, 703, 653, 532 cm^{-1}.

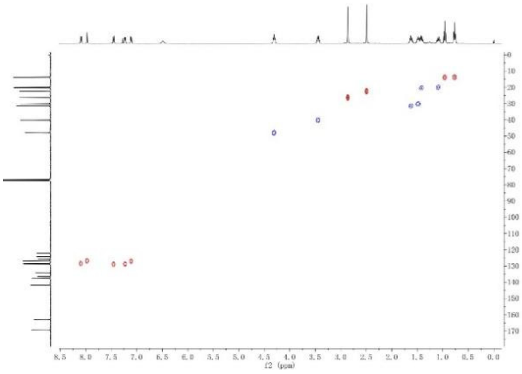

Full 2D NMR HSQC

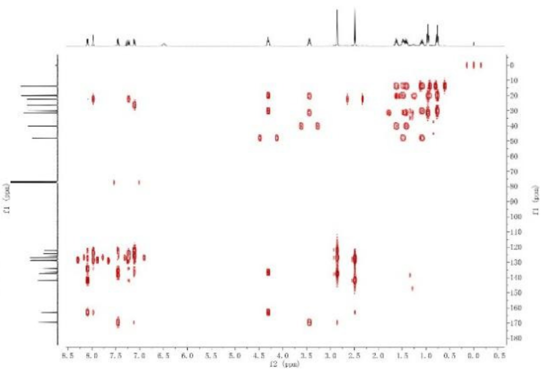

Full 2D NMR HMBC

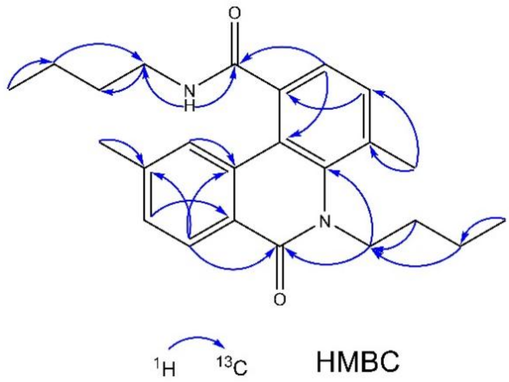

Structural Analysis

^{1}H and ^{13}C NMR spectra of compound **2q**.

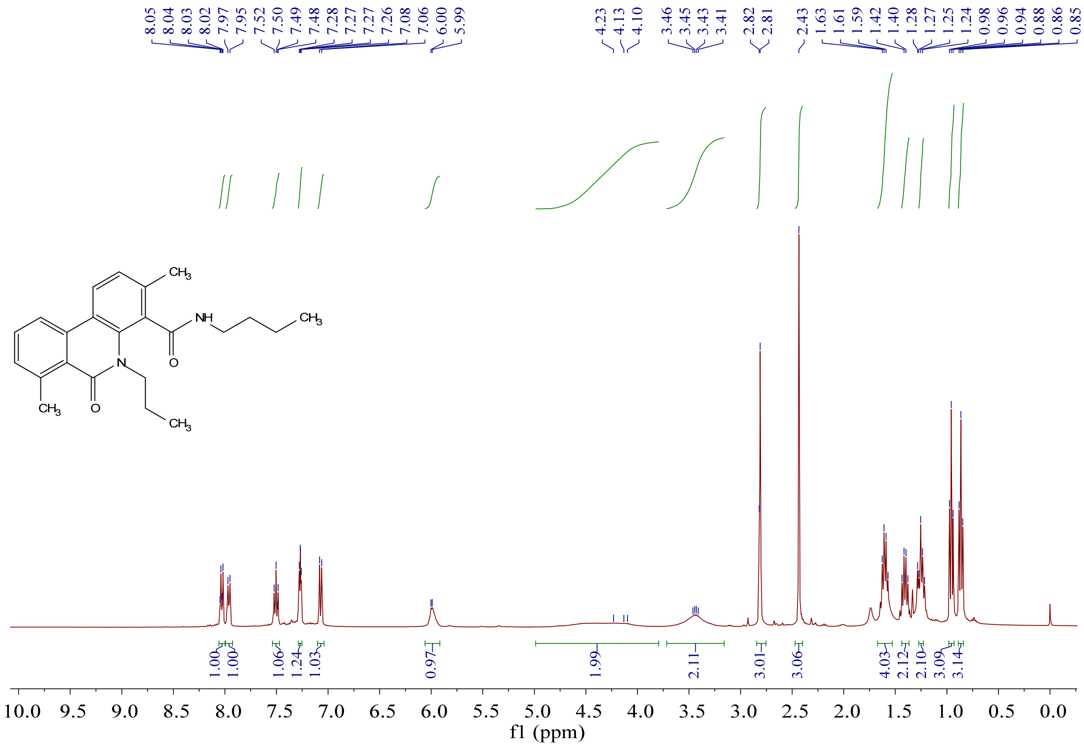

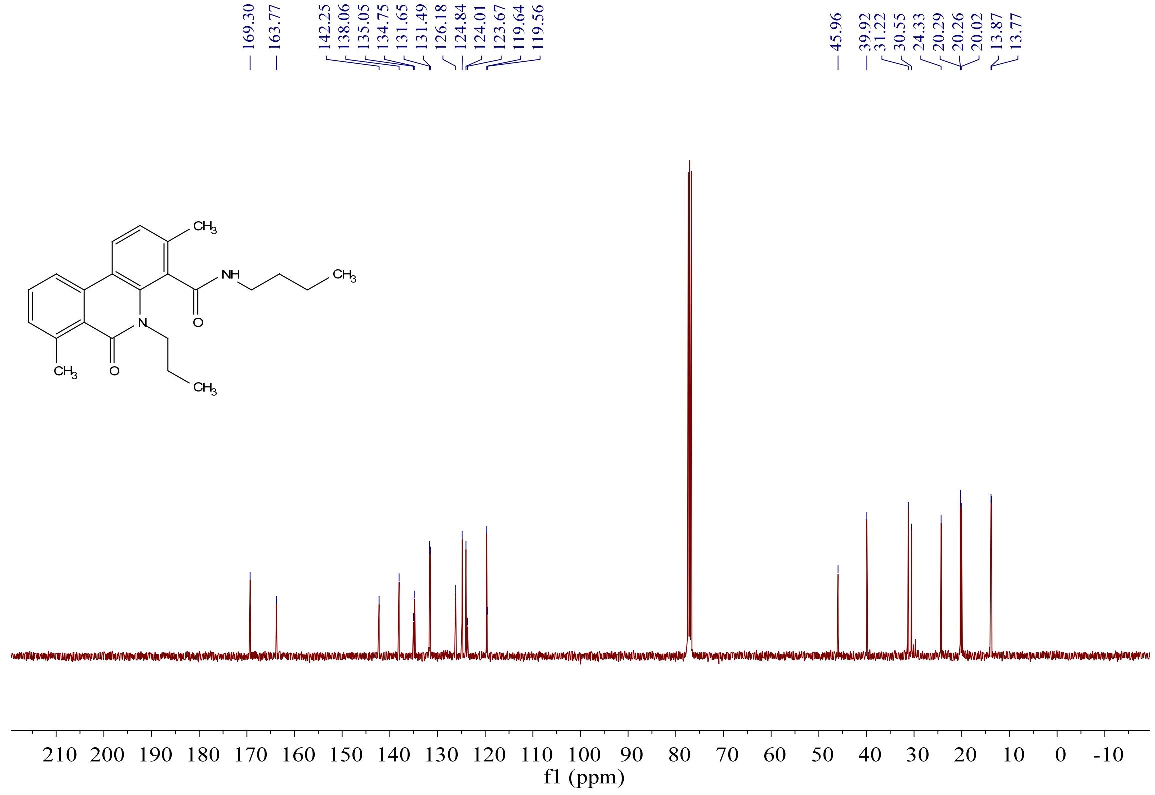

HRMS spectra of compound **2q**.

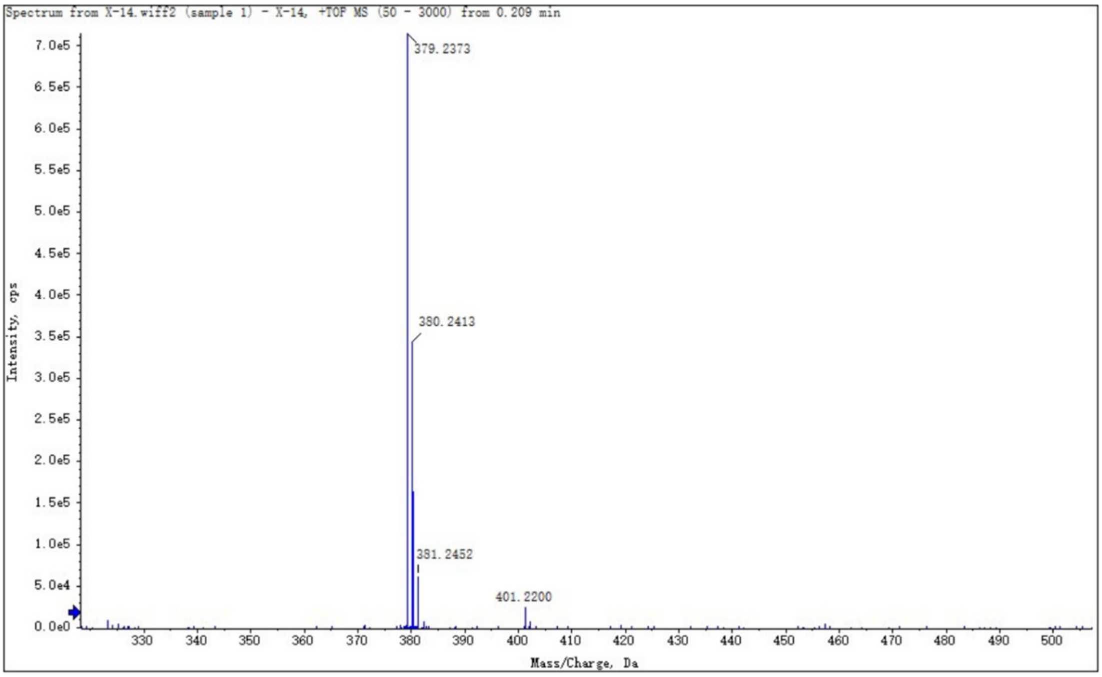

IR spectra of compound **2q**.

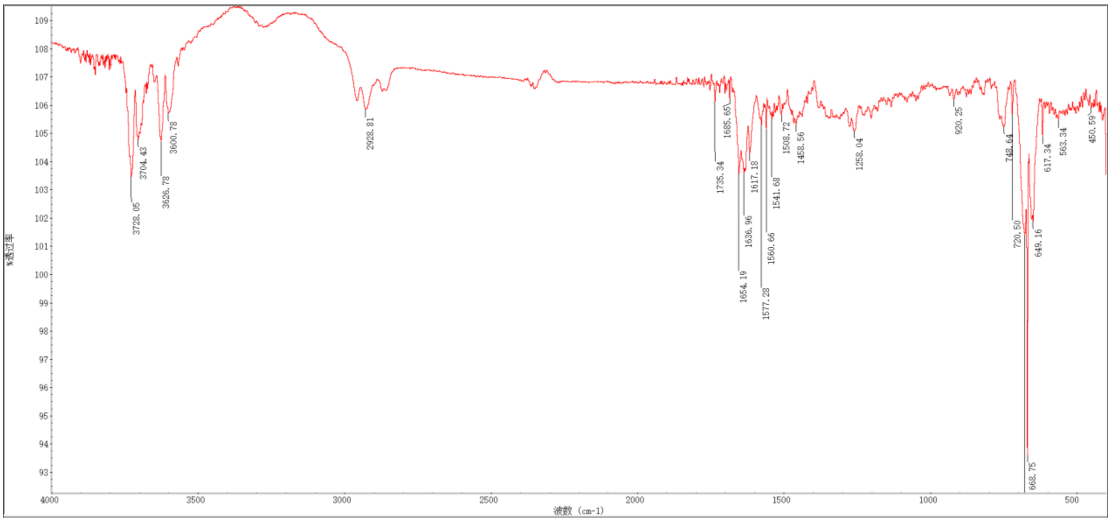

N,5-dibutyl-3,7-dimethyl-6-oxo-5,6-dihydrophenanthridine-4-carboxamide (2q):

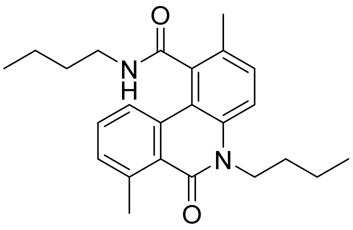

2q

White solid, yield 77%; ^{1}**H NMR** (400 MHz, $CDCl_3$) δ 8.03 (dd, J = 8.2, 3.5 Hz, 1H), 7.96 (d, J = 8.2 Hz, 1H), 7.50 (dd, J = 10.4, 5.1 Hz, 1H), 7.27 (dd, J = 4.2, 3.1 Hz, 1H), 7.07 (d, J = 8.2 Hz, 1H), 5.99

(d, J = 5.2 Hz, 1H), 4.99 – 3.79 (m, 2H), 3.44 (dd, J = 13.4, 6.8 Hz, 2H), 2.81 (d, J = 3.7 Hz, 3H), 2.43 (s, 3H), 1.60 (dd, J = 14.9, 7.4 Hz, 4H), 1.41 (dd, J = 15.0, 7.5 Hz, 2H), 1.24 (d, J = 7.2 Hz, 2H), 0.96 (t, J = 7.3 Hz, 3H), 0.86 (t, J = 7.3 Hz, 3H). **^{13}C NMR** (101 MHz, $CDCl_3$) δ 169.30, 163.77, 142.25, 138.06, 135.05, 134.75, 131.65, 131.49, 126.18, 124.84, 124.01, 123.67, 119.64, 119.56, 45.96, 39.92, 31.22, 30.55, 24.33, 20.29, 20.26, 20.02, 13.87, 13.77. **HRMS** (ESI-TOF) m/z: $[M+H]^+$ calcd for $C_{24}H_{31}N_2O_2$ 379.2380; found 379.2373. **IR** (KBr): 3728, 3627, 2929, 1654, 1637, 1617, 1577, 1542, 1258, 920, 749, 721, 669, 617, 532 cm^{-1}.

^{1}H, ^{13}C, and ^{19}F NMR spectra of compound **2r**.

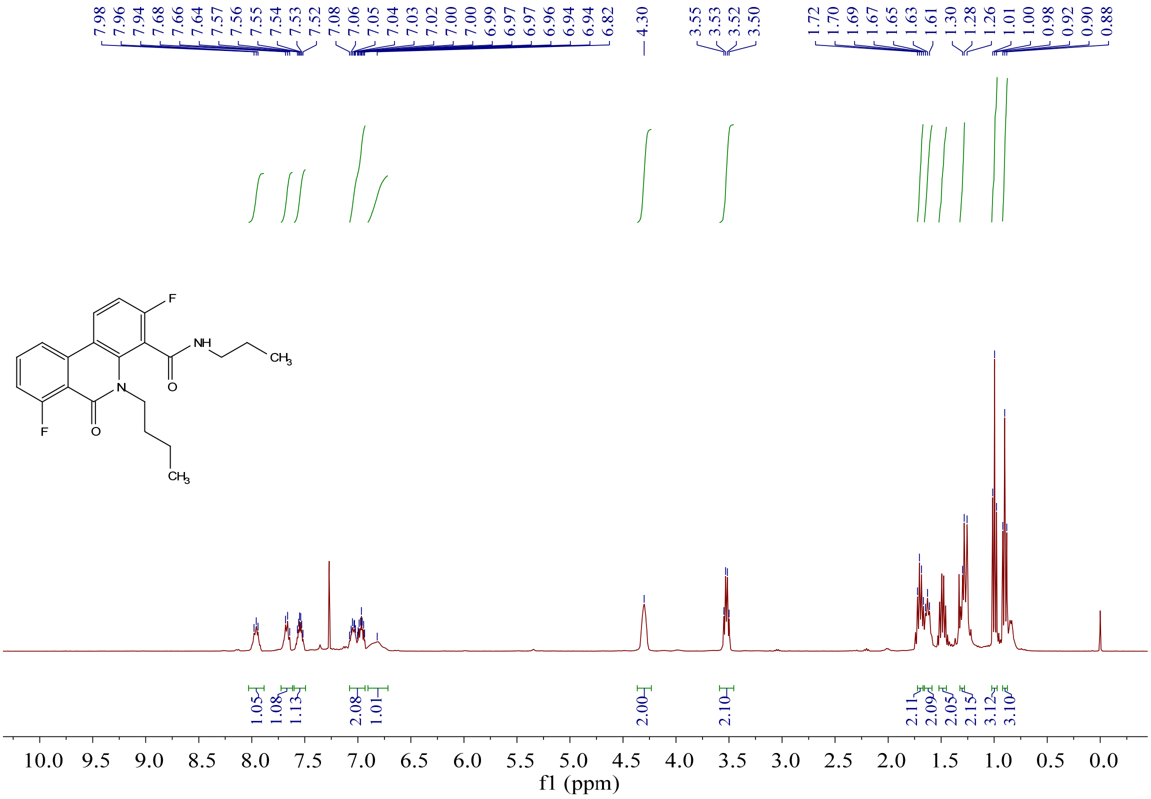

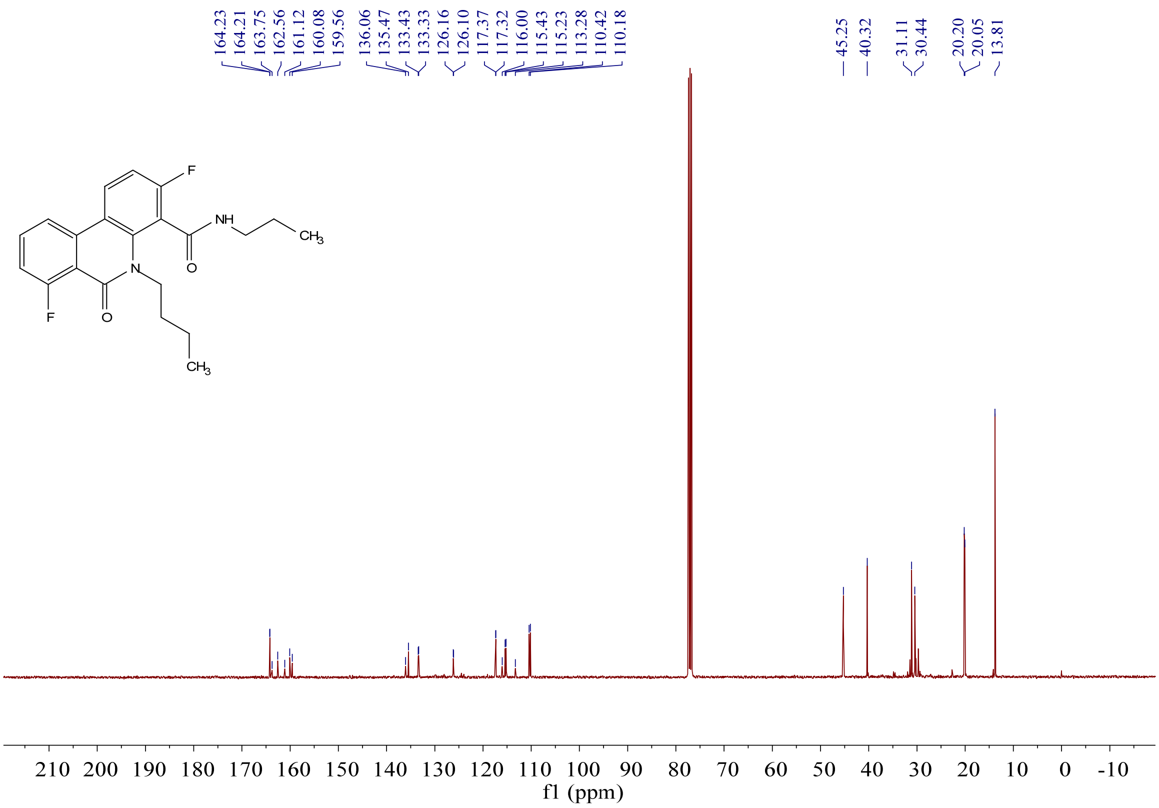

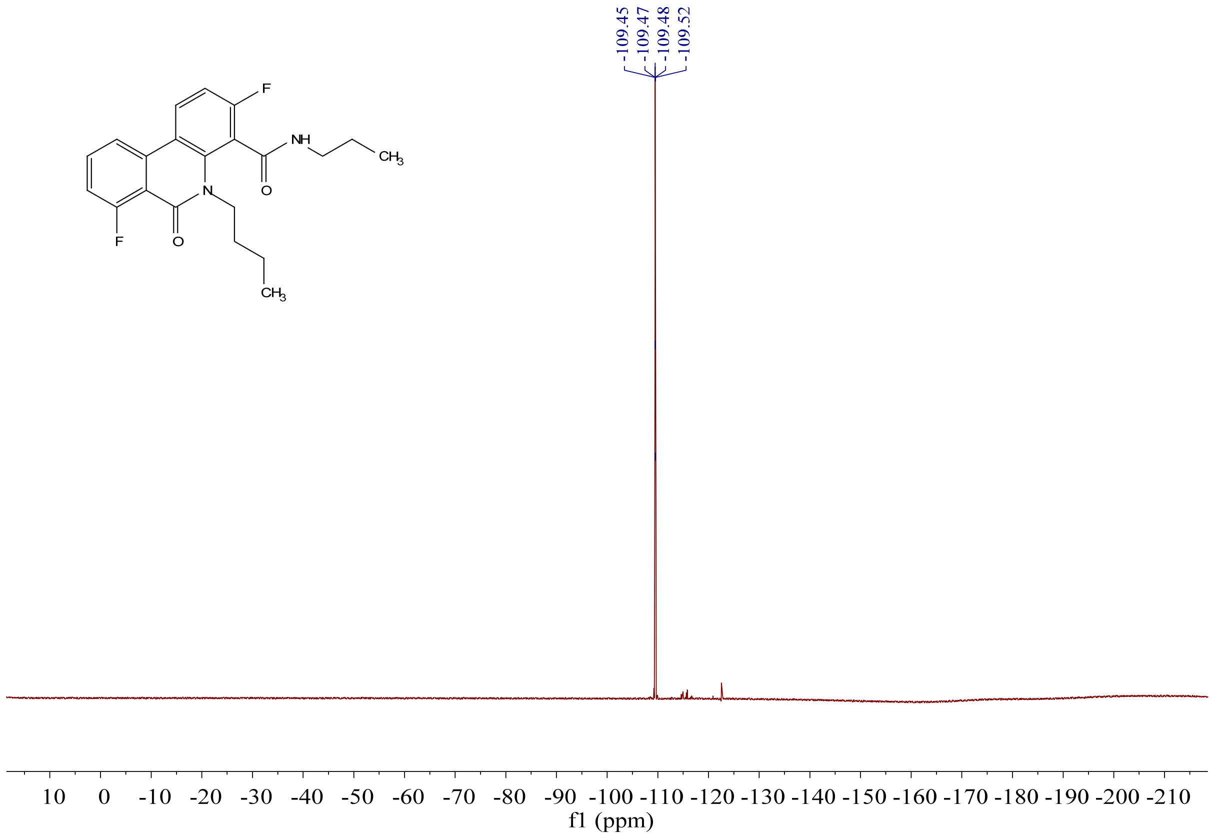

N,5-dibutyl-3,7-difluoro-6-oxo-5,6-dihydrophenanthridine-4-carboxamide (2r):

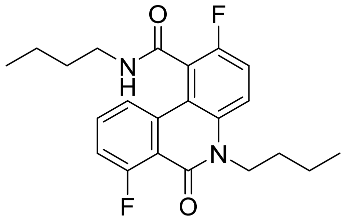

2r

White solid, yield 64%; **^{1}H NMR** (400 MHz, $CDCl_3$) δ 8.03 – 7.88 (m, 1H), 7.72 – 7.61 (m, 1H), 7.54 (dt, J = 8.2, 4.6 Hz, 1H), 7.08 – 6.93 (m, 2H), 6.82 (s, 1H), 4.30 (s, 2H), 3.52 (dd, J = 13.3, 6.8 Hz, 2H), 1.72 – 1.67 (m, 2H), 1.66 – 1.59 (m, 2H), 1.49 (dd, J = 15.1, 7.5 Hz, 2H), 1.29 (d, J = 5.7 Hz, 2H), 1.00 (t, J = 7.4 Hz, 3H), 0.90 (t, J = 7.4 Hz, 3H). **^{13}C NMR** (101 MHz, $CDCl_3$) δ 164.23, 164.21, 163.75, 162.56, 161.12, 160.08, 159.56, 136.06, 135.47, 133.43, 133.33, 126.16, 126.10, 117.37, 117.32, 116.00, 115.43, 115.23, 113.28, 110.42, 110.18, 45.25, 40.32, 31.11, 30.44, 20.20, 20.05, 13.81. **^{19}F NMR** (376 MHz, $CDCl_3$) δ -109.45, -109.47, -109.48, -109.52.

^{1}H and ^{13}C NMR spectra of compound **2s**.

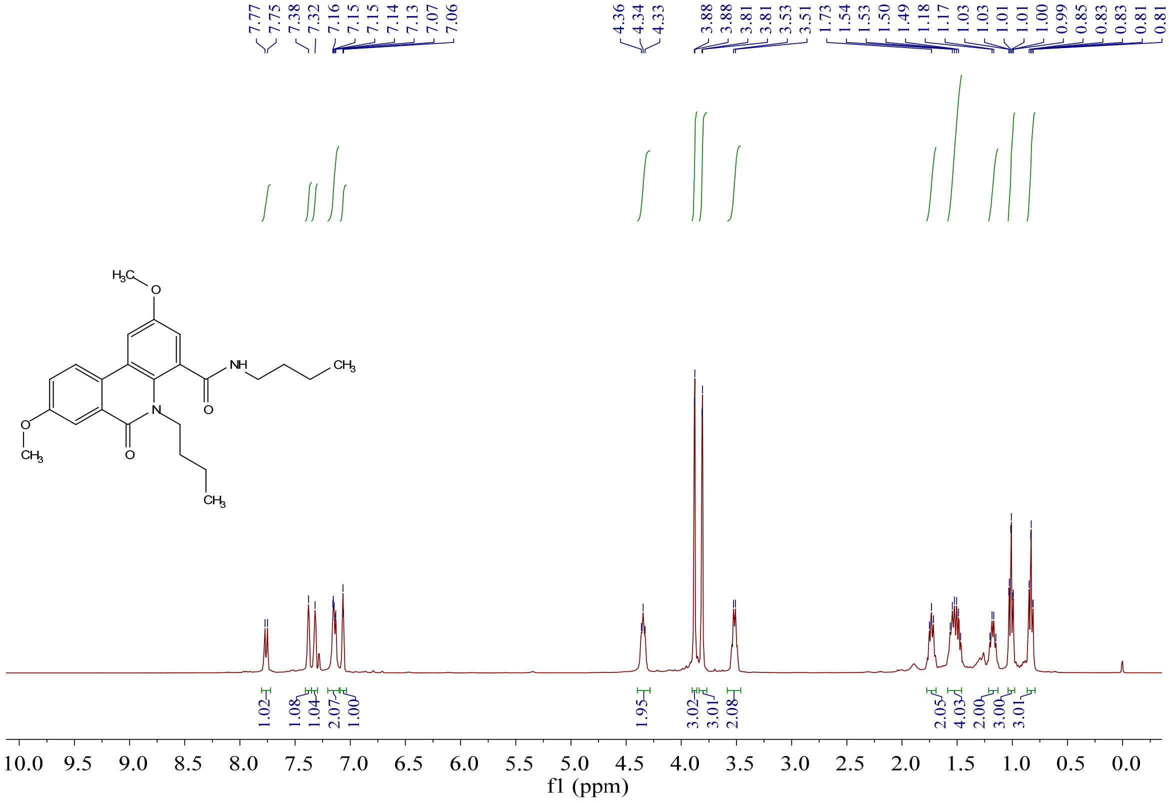

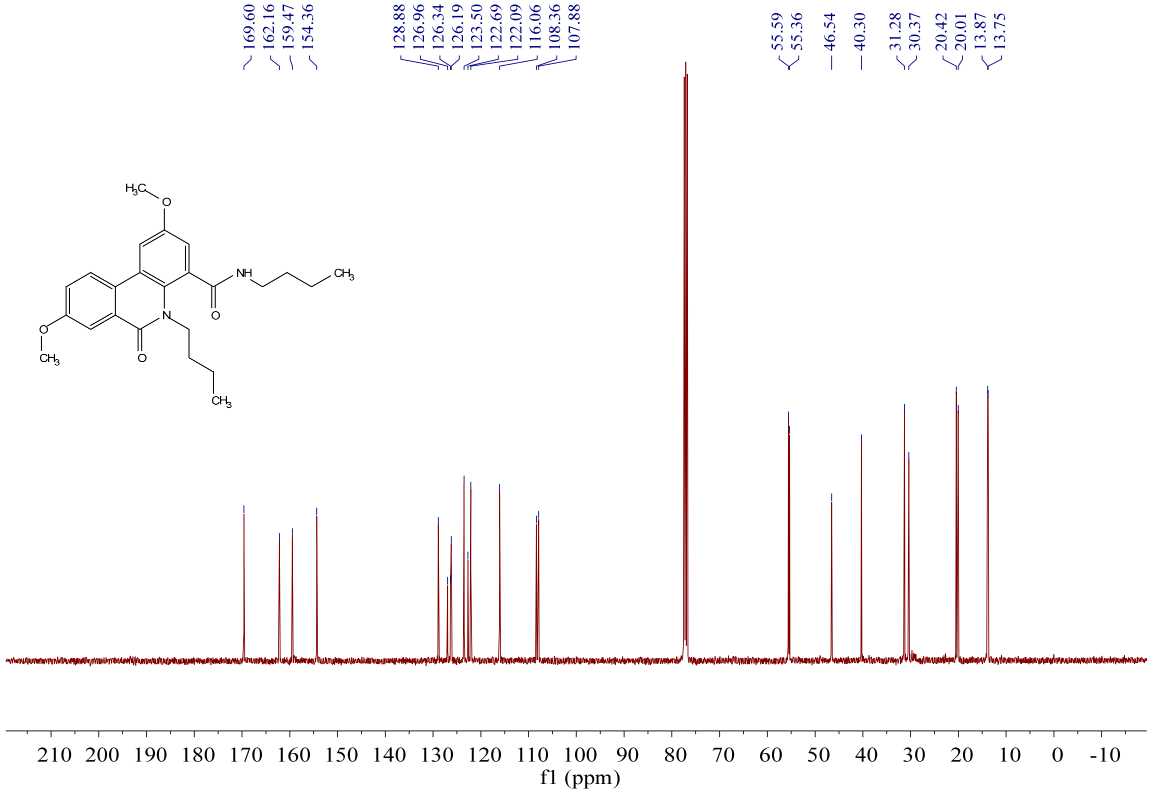

HRMS spectra of compound **2s**.

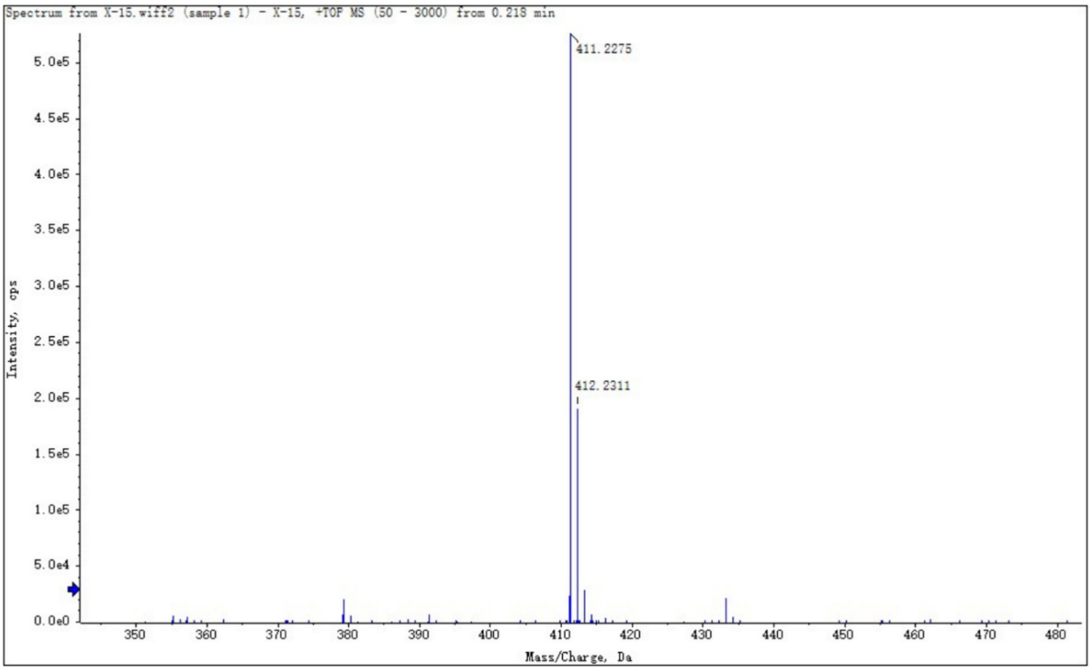

IR spectra of compound **2s**.

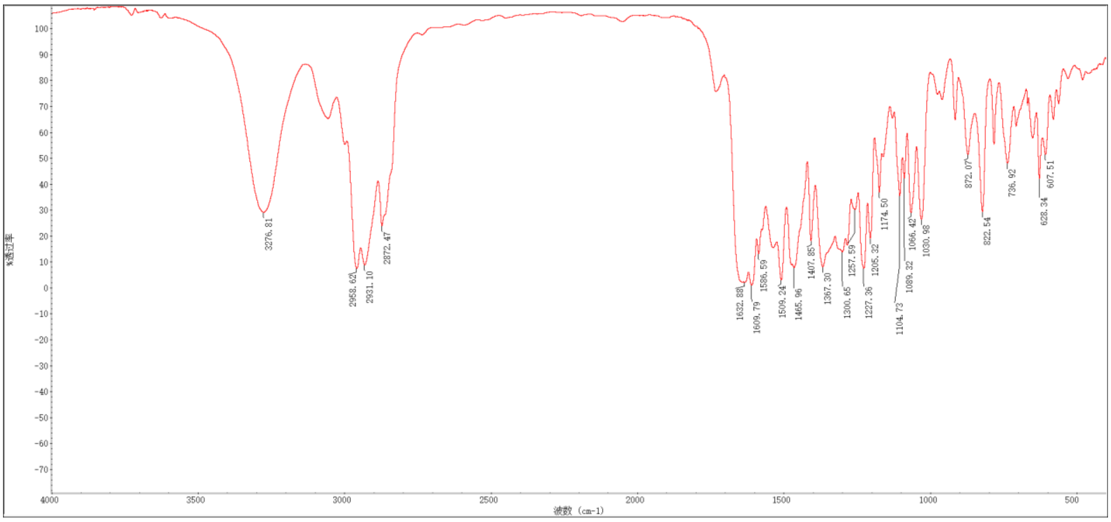

N,5-dibutyl-2,8-dimethoxy-6-oxo-5,6-dihydrophenanthridine-4-carboxamide (2s):

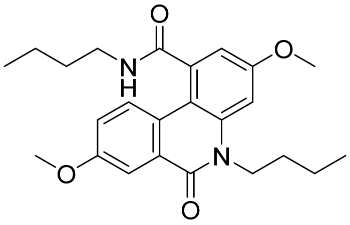

2s

White solid, yield 85%; **^{1}H NMR** (400 MHz, $CDCl_3$) δ 7.76 (d, *J* = 8.9 Hz, 1H), 7.38 (s, 1H), 7.32 (s, 1H), 7.20 – 7.10 (m, 2H), 7.06 (d, *J* = 2.4 Hz, 1H), 4.34 (t, *J* = 6.7 Hz, 2H), 3.88 (d, *J* = 1.8 Hz, 3H),

3.81 (d, *J* = 1.5 Hz, 3H), 3.52 (d, *J* = 6.9 Hz, 2H), 1.78 – 1.69 (m, 2H), 1.52 (dt, *J* = 22.6, 7.2 Hz, 4H), 1.18 (dd, *J* = 14.0, 7.1 Hz, 2H), 1.01 (td, *J* = 7.3, 1.9 Hz, 3H), 0.86 – 0.79 (m, 3H). **^{13}C NMR** (101 MHz, $CDCl_3$) δ 169.60, 162.16, 159.47, 154.36, 128.88, 126.96, 126.34, 126.19, 123.50, 122.69, 122.09, 116.06, 108.36, 107.88, 55.59, 55.36, 46.54, 40.30, 31.28, 30.37, 20.42, 20.01, 13.87, 13.75. **HRMS** (ESI-TOF) m/z: $[M+H]^+$ calcd for $C_{24}H_{31}N_2O_4$ 411.2279; found 411.2275. **IR** (KBr): 3277, 2959, 2931, 2872, 1633, 1610, 1587, 1509, 1466, 1408, 1367, 1301, 1258, 1227, 1175, 1089, 1031, 872, 823, 737, 628, 608, 532 cm^{-1}.

^{1}H and ^{13}C NMR spectra of compound **2t**.

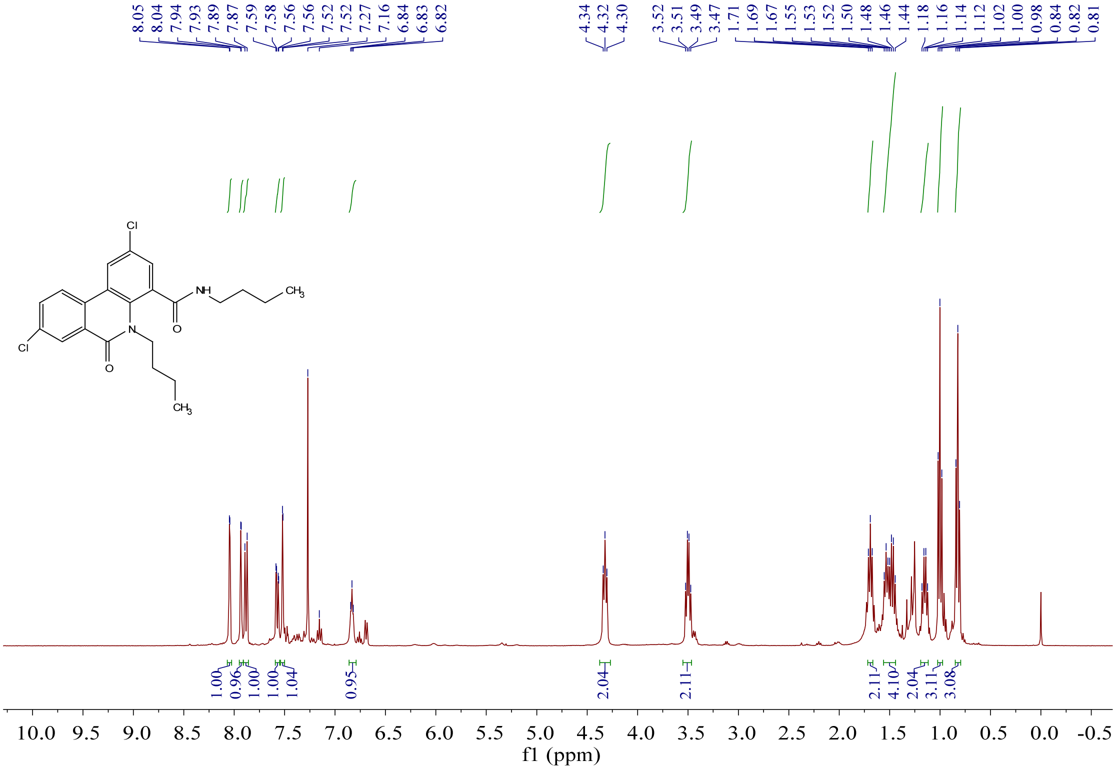

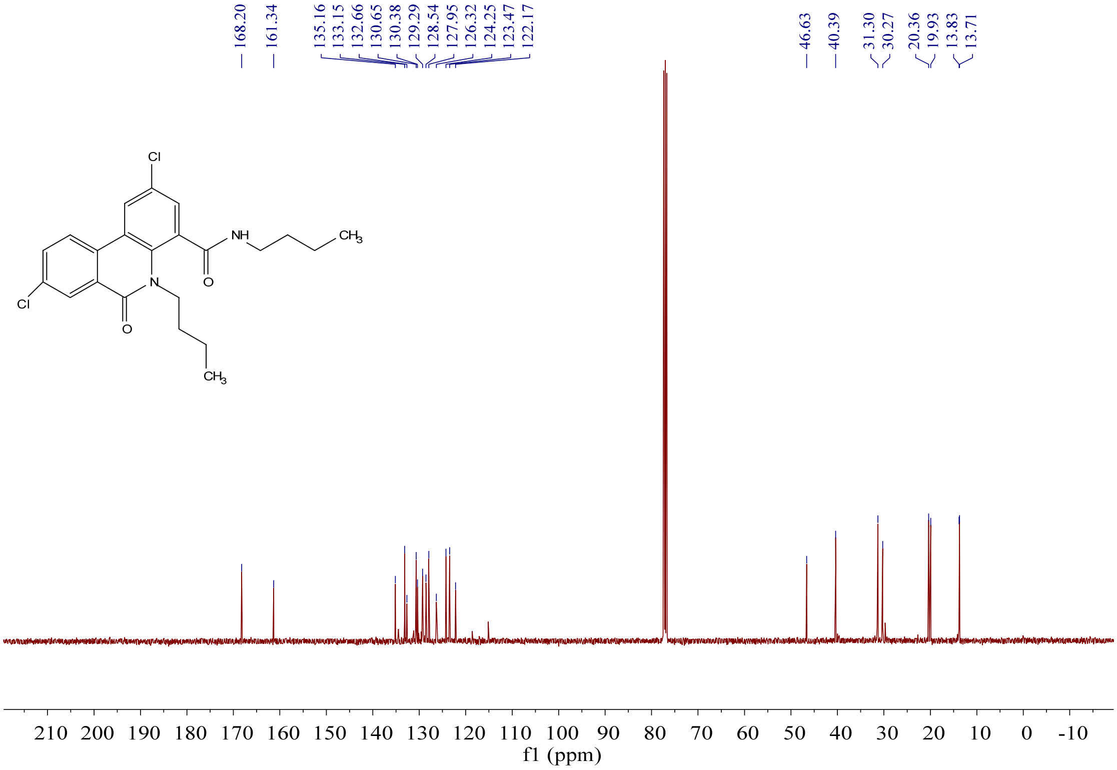

HRMS spectra of compound **2t**.

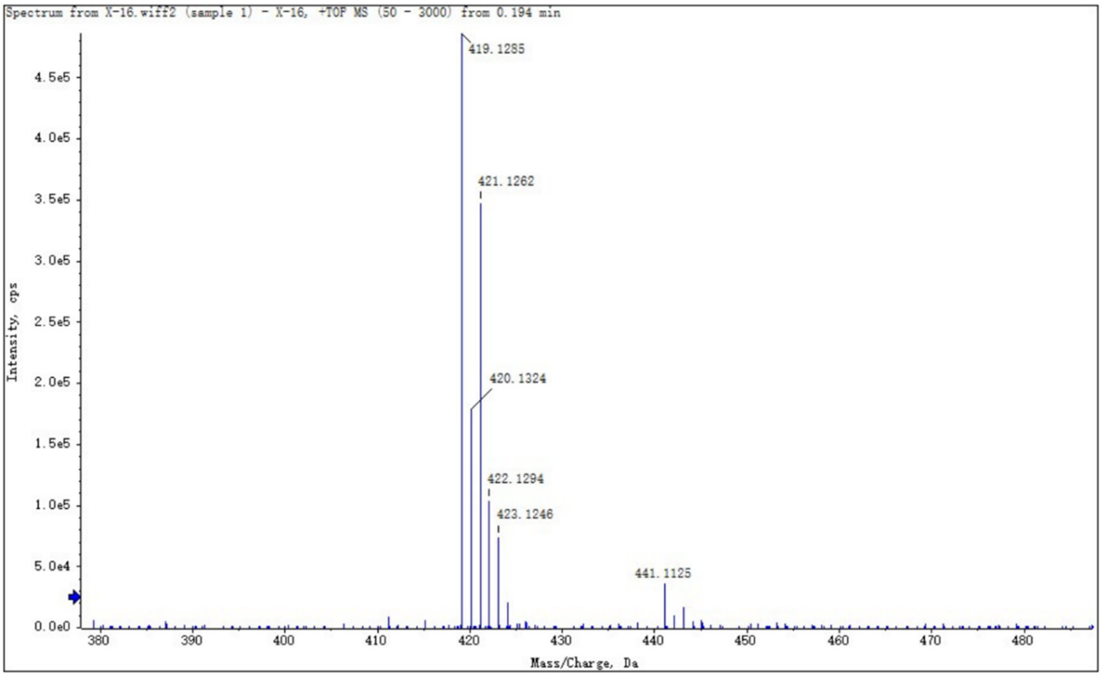

N,5-dibutyl-2,8-dichloro-6-oxo-5,6-dihydrophenanthridine-4-carboxamide (2t):

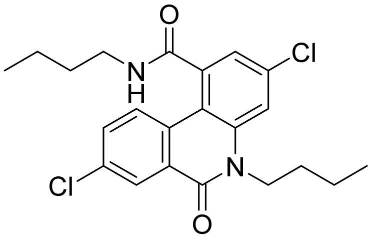

2t

White solid, yield 83%; **^{1}H NMR** (400 MHz, $CDCl_3$) δ 8.05 (d, *J* = 2.2 Hz, 1H), 7.93 (d, *J* = 1.9 Hz, 1H), 7.88 (d, *J* = 8.8 Hz, 1H), 7.57 (dd, *J* = 8.7, 2.1 Hz, 1H), 7.52 (d, *J* = 2.2 Hz, 1H), 6.83 (t, *J* = 5.0 Hz, 1H), 4.32 (t, *J* = 7.4 Hz, 2H), 3.50 (dd, *J* = 13.3, 6.9 Hz, 2H), 1.72 – 1.67 (m, 2H), 1.56 – 1.44 (m, 4H), 1.15 (dd, *J* = 15.1, 7.5 Hz, 2H), 1.00 (t, *J* = 7.4 Hz, 3H), 0.82 (t, *J* = 7.3 Hz, 3H). **^{13}C NMR** (101 MHz, $CDCl_3$) δ 168.20, 161.34, 135.16, 133.15, 132.66, 130.65, 130.38, 129.29, 128.54, 127.95, 126.32, 124.25, 123.47, 122.17, 46.63, 40.39, 31.30, 30.27, 20.36, 19.93, 13.83, 13.71. **HRMS** (ESI-TOF) m/z: $[M+H]^+$ calcd for $C_{22}H_{25}Cl_2N_2O_2$ 419.1288; found 419.1285.

^1H and ^{13}C NMR spectra of compound **2u**.

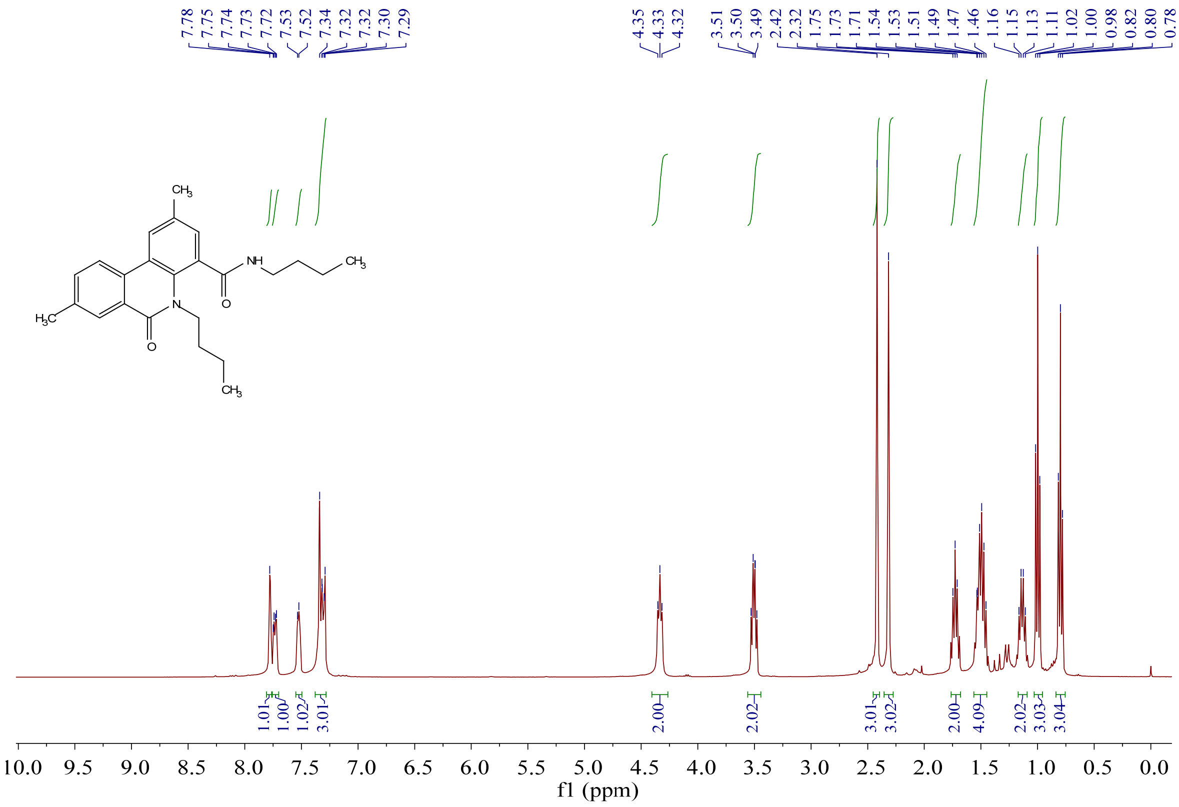

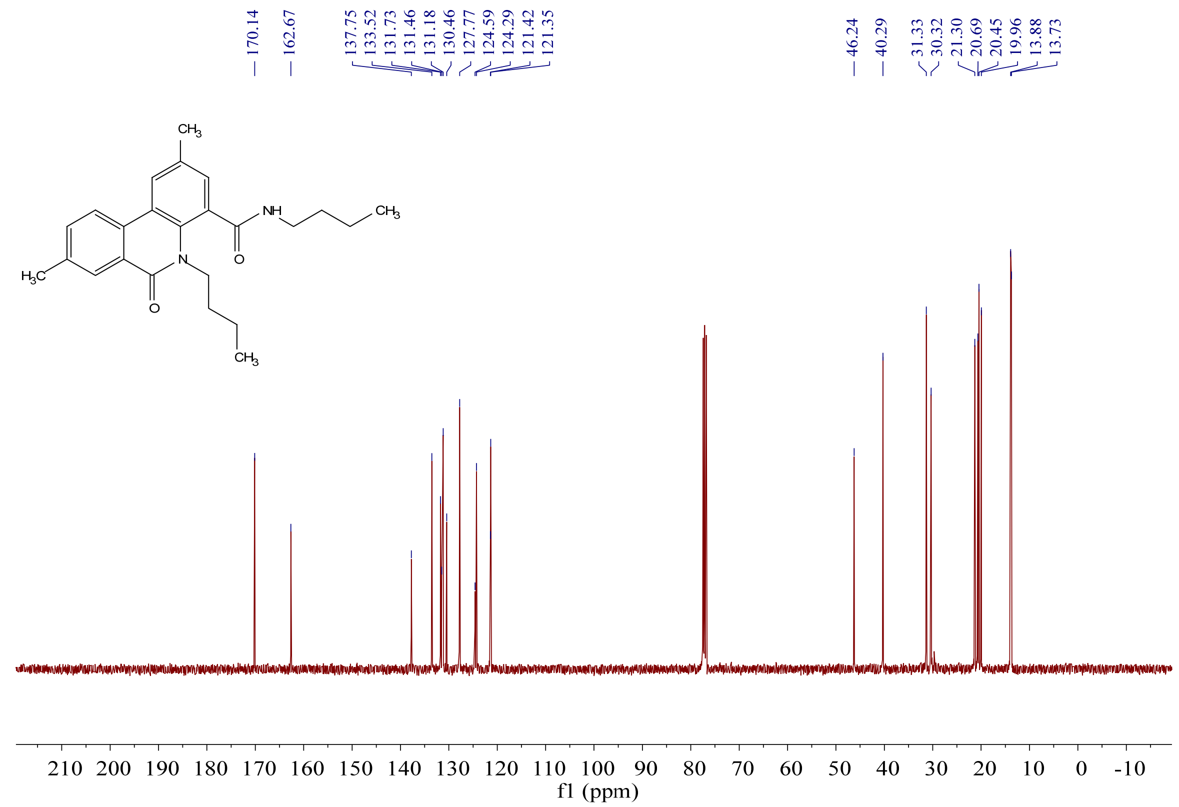

N,5-dibutyl-2,8-dimethyl-6-oxo-5,6-dihydrophenanthridine-4-carboxamide (2u):

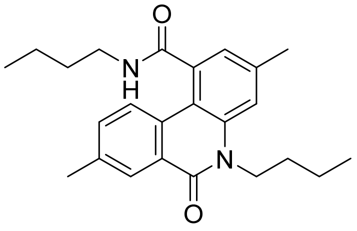

2u

White solid, yield 86%， m.p. 125-127°C; ^{1}**H NMR** (400 MHz, $CDCl_3$) δ 7.78 (s, 1H), 7.73 (dd, *J* = 8.1, 3.1 Hz, 1H), 7.53 (d, *J* = 4.6 Hz, 1H), 7.38 – 7.28 (m, 3H), 4.33 (t, *J* = 7.2 Hz, 2H), 3.56 – 3.44 (m, 2H), 2.42 (s, 3H), 2.32 (s, 3H), 1.73 (t, *J* = 7.5 Hz, 2H), 1.56 – 1.45 (m, 4H), 1.14 (dd, *J* = 15.2, 7.5 Hz, 2H), 1.00 (t, *J* = 7.4 Hz, 3H), 0.80 (t, *J* = 7.4 Hz, 3H). ^{13}**C NMR** (101 MHz, $CDCl_3$) δ 170.14, 162.67, 137.75, 133.52, 131.73, 131.46, 131.18, 130.46, 127.77, 124.59, 124.29, 121.42, 121.35, 46.24, 40.29, 31.33, 30.32, 21.30, 20.69, 20.45, 19.96, 13.88, 13.73.
